# Point-of-care lung ultrasound predicts hyperferritinemia and hospitalization, but not elevated troponin in SARS-CoV-2 viral pneumonitis in children

**DOI:** 10.1038/s41598-024-55590-9

**Published:** 2024-03-11

**Authors:** Paul Walsh, Andrea Hankins, Heejung Bang

**Affiliations:** 1 Pediatric Emergency Medicine, Sutter Medical Center Sacramento, 2825 Capitol Avenue, Sacramento, CA USA; 2 Sutter Institute for Medical Research, 2801 L Street, Sacramento, CA USA; 3 Division of Biostatistics, Department of Public Health Sciences, University of California Davis, 1 Shields Ave, Davis, CA USA; 4 Sutter Health Center for Health Systems Research, Sutter Health, Walnut Creek, CA USA

**Keywords:** Paediatric research, SARS-CoV-2

## Abstract

SARS-CoV-2 often causes viral pneumonitis, hyperferritinemia, elevations in D-dimer, lactate dehydrogenase (LDH), transaminases, troponin, CRP, and other inflammatory markers. Lung ultrasound is increasingly used to diagnose and stratify viral pneumonitis severity. We retrospectively reviewed 427 visits in patients aged 14 days to 21 years who had had a point-of-care lung ultrasound in our pediatric emergency department from 30/November/2019 to 14/August/2021. Lung ultrasounds were categorized using a 6-point ordinal scale. Lung ultrasound abnormalities predicted increased hospitalization with a threshold effect. Increasingly abnormal laboratory values were associated with decreased discharge from the ED and increased admission to the ward and ICU. Among patients SARS-CoV-2 positive patients ferritin, LDH, and transaminases, but not CRP or troponin were significantly associated with abnormalities on lung ultrasound and also with threshold effects. This effect was not demonstrated in SARS-CoV-2 negative patients. D-Dimer, CRP, and troponin were sometimes elevated even when the lung ultrasound was normal.

## Introduction

Lung ultrasound has been validated for the diagnosis of viral, including SARS-CoV-2, pneumonitis using a CT gold standard in adults and older children^1–8^ and has also been shown to predict which patients are more likely to deteriorate^9–11^. See *Buonsenso and Vetrugno 2022* for a review of this^12^.

Certain blood tests have also been shown to herald increased morbidity or mortality in SARS-CoV-2 infection e.g. Ferritin^13–16^, LDH^17–19^, ALT, AST^17,20^, D-dimer^21,22^, CRP^17^, ESR^17^, Procalcitonin^17^, BNP^17^, and possibly WBC^17^. Elevated troponin implies potentially significant myocarditis^23,24^. Associations between increasingly abnormal laboratory values and lung ultrasound severity have been noted in adults^25^ with SARS-CoV-2 infection.

This raises the question: Can lung ultrasound be used to screen which children need blood testing? Such an approach is inherently attractive in the pediatric emergency department (ED); bedside lung ultrasound can be performed rapidly and is minimally invasive. Conversely, obtaining blood tests in children imposes substantial costs in terms of staff time and emotional distress to both the child and parents. Avoiding blood tests allows for faster discharge and, with the large numbers of children typically seen in a pediatric ED is mathematically the equivalent of increasing the number of ED beds. From a health system perspective, this is an important possibility to explore.

We asked three questions: Among children presenting to the pediatric ED for potential SARS-CoV-2 infection:

1. Does the severity of lung ultrasound abnormalities predict clinical outcomes (measured as ED discharge versus hospitalization)?
2. Do abnormal blood tests predict clinical outcomes in children (measured as ED discharge versus admission to the ward or admission to the pediatric intensive care unit (PICU)?
3. Can the lung ultrasound be used to determine which children need blood tests?

Answering these questions is complicated by the fact that SARS-CoV-2 testing results were not available during the patients’ initial evaluation when lung ultrasounds are performed, and further challenged by selection bias; namely, the decision to obtain blood tests on a patient may also be influenced by the ultrasound findings. Inevitably some of the children in whom the emergency physicians suspect SARS-CoV-2 will have a different virus; but treatment and discharge decisions must be made before the virus can be confirmed. Consequently, we must simultaneously address the potential for incorporation bias from knowledge of the point-of-care lung ultrasound findings, and also not assume future knowledge (virus test results) not available to the emergency physician.

However, such an analysis is inherently unsatisfying to intensive care and ward physicians who will have access to lung ultrasound results, blood results, and viral test results; these physicians may see little value in studies based on noisier undifferentiated samples diluted by other viruses. We present two sets of analyses, one with all-comers (representing pediatric emergency medicine outpatient practice) and one with SARS-CoV-2 positive tests (representing inpatient practice) results to satisfy both groups of physicians.

## Results

### Patients

We included 427 patient visits in our analysis. The median patient age was 6 years and 229/427 (54%) were male. Thirty-eight, (9%) patients were admitted and 84 (20%) had at least one blood test performed. Nasal turbinate or nasopharyngeal nucleic acid-based testing for SARS-CoV2 testing was performed on 398 visits and was positive in 108 (27%). Patient characteristics are shown in Table 1. Another virus was identified in 98 visits, most commonly rhinovirus (73), another coronavirus (10), or a parainfluenza virus (7). Most lung ultrasounds were normal or showed only mild disease. Lung ultrasound findings are summarized in Table 2.

**Table 1:** Patient characteristics for all comers, those who were subsequently SARS-Cov-2 positive and negative by nucleic acid testing on nasal or nasal pharyngeal swab, and those in whom SARS-CoV-2 testing was not available or was indeterminate.

Factor	Level/Unit	All comers	SARS-Cov-2 Positive (*)	SARS-Cov-2 Negative (*)	SARS-Cov-2 Unknown(**)
		N (%)	N (%)	N (%)	N (%)
		427 (100%)	118/216	98/216	211/211
Male		229 (54%)	56 (47)	60 (61)	113 (54)
Age, median (IQR)	(years)	6.2 (1.7, 13.9)	12.6 (3.7, 17.0)	2.7 (0.9, 6.0)	6.8 (2.2 12.9)
Age category	Neonate < 1 month	10 (2%)	6 (6%)	2 (2)	2 (1)
	Infant 1 to 12 month	58 (14%)	13 (11%)	24 (24)	21 (10)
	Preschooler 1–5 years	140 (33%)	19 (16%)	47 (48)	74 (35)
	School-age 6–12 years	88 (21%)	18 (15%)	16(16)	54 (26)
	Teenager 13–18	84 (20%)	43 (36%)	8 (8)	33 (16)
	Transitional 18–21 years	47 (11%)	19 (16%)	1 (1)	27 (13)
ESI triage category	1 (most severe)	1 (0.2%)	0(0%)	0 (0)	1 (0.5)
	2	37 (9%)	11 (9%)	9(9)	17 (8)
	3	116 (27%)	23 (19%)	29 (30)	64(30)
	4	255 (60%)	73 (62%)	58(59)	124(59)
	5 (least severe)	18 (4%)	11 (9%)	2 (2)	5 (2)
ED length of stay if discharged	(minutes)	118 (80, 171)	114 (78, 179)	123 (82 , 178)	118 (82, 165)
Blood test (***)	Any/None	98 (23%)	27/118 (23%)	22 (22%)	49 (23%)
Admitted		41 (10%)	9 (8%)	11 (11%)	21 (10%)
Year enrolled	2019 (Dec only)	5 (1%)	0 (0%)	3 (3%)	2 (1%)
	2020 (12 months)	233 (55%)	76 (64%)	35 (36%)	122 (58%)
	2021 (8 months)	189 (44%)	42 (36%)	60 (61%)	87 (41%)

*False negatives can be anticipated in the context of initial presentations of airborne respiratory viruses. **Includes patients in whom an alternative virus and in whom no virus was detected on an extended respiratory panel. ***Excludes cases where blood tests were ordered but no result was reported by the clinical laboratory. Percentage totals may exceed 100% due to rounding. Admission data excludes one death in a medically complex child and includes a patient who was boarded in the ED for his entire stay) ED pediatric emergency department; IQR Interquartile range; ESI emergency severity index.

**Table 2:** Point of care lung ultrasound interpretations and clinical outcomes.

Lung	All comers				SARS-Cov-2 Positive				SARS-Cov-2 Negative				SARS-Cov-2 Unknown			
Ultrasound	Total	Discharge	Ward	PICU	Total	Discharge	Ward	PICU	Total	Discharge	Ward	PICU	Total	Discharge	Ward	PICU
Normal	200	190 (95)	9 (4.5)	1 (0.5)	51	51 (100)	0 (0)	0 (0)	39	36 (92)	2 (5)	1 (3)	110	103 (94)	7 (6)	0 (0)
Very Mild	68	67 (99)	1 (1)	0 (0)	19	19 (100)	0 (0)	0 (0)	15	15 (100)	0 (0)	0 (0)	34	33 (97)	1 (3)	0 (0)
Mild	68	65 (96)	3 (4)	0 (0)	24	24(100)	0 (0)	0 (0)	19	17 (89)	2 (11)	0 (0)	25	24 (96)	1 (4)	0 (0)
Mild to moderate	30	24 (80)	3 (10)	3 (10)	3	3 (100)	0 (0)	0 (0)	11	9 (82)	2 (18)	0 (0)	16	12 (75)	1 (6)	3 (19)
Moderate	42	25 (62)	12 (29)	4 (10)	14	7 (50)	6 (43)	1 (7)	11	7 (64)	2 (18)	2 (18)	17	12 (71)	4 (24)	1 (6)
Mod/Severe	19	14 (74)	4 (21)	1 (5)	7	5 (71)	1 (14)	1 (14)	3	3 (100)	0 (0)	0(0)	9	6 (67)	3 (33)	0 (0)
	427 (100)	386 (90)	32 (7)	9 (2)	118 (100)	109 (92)	7 (6)	2 (2)	98 (100)	87 (89)	8 (3)	3	211 (100)	190 (90)	17 (8)	4 (2)

Lung ultrasounds’ severity originally recorded in the medical record was grouped for analysis**;** the ‘Mod/Severe’ category includes 7 ‘moderate to severe’, and 12 ‘severe’ cases. Percentages are affected by rounding. The all-comers group reflects what the emergency physician will see on arrival prior to tests being available. The unknown group refers to those for whom SARS-Cov-2 testing was either unavailable or for whom it was refused.

### Ultrasounds

There were 500 point-of-care lung ultrasounds reported in pediatric ED medical records during the study period. Of these 427 (visits representing 371 distinct patients) could be assigned a severity category based on the physicians’ written ultrasound report. The ultrasonographers, all physicians, categorized their reports using a 7-point scale from normal to severe. This level of apparent precision was not supported by the outcomes data: there was little difference in outcomes between the ‘Normal’, ‘Very mild’, and ‘Mild’ categories. We did not find differences in the distribution of lung ultrasound classifications between the pediatric emergency physicians, general emergency physicians and residents who performed the ultrasounds.

**Figure 1:**
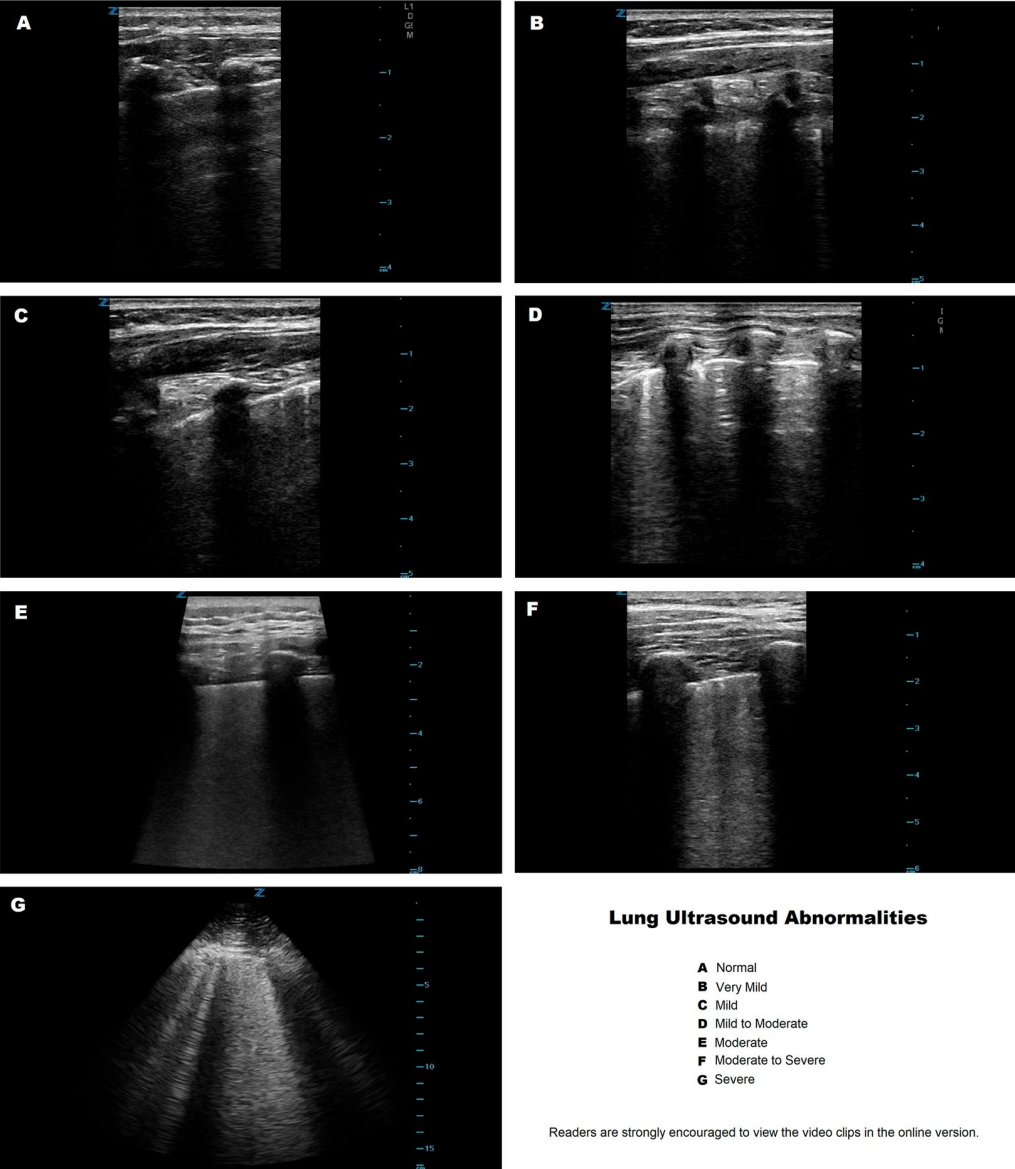
Representative sample of images for each category.

Sample ultrasound clips for each classification are provided in the supplemental materials. Still images are included in Fig. 1.

The intra-rater agreement for the classification of ultrasound reports was high; Gwet’s AC-1 coefficient for agreement was 0.82, and the intra-class correlation for intra-rater reliability for lung ultrasound was 0.92.

### Clinical outcomes

Our clinical outcomes were ED discharge versus hospitalization, and if hospitalized the level of care required: either hospital ward or pediatric intensive care. (The number of deaths was too low to allow meaningful analysis.) Most visits, 386/427 (90%), ended in discharge from the ED. Thirty two (7.5%) were admitted to the ward, and 9/427 (2%) were admitted to the PICU. The breakdown by viral etiology is in Table 1**.**

**Figure 2:**
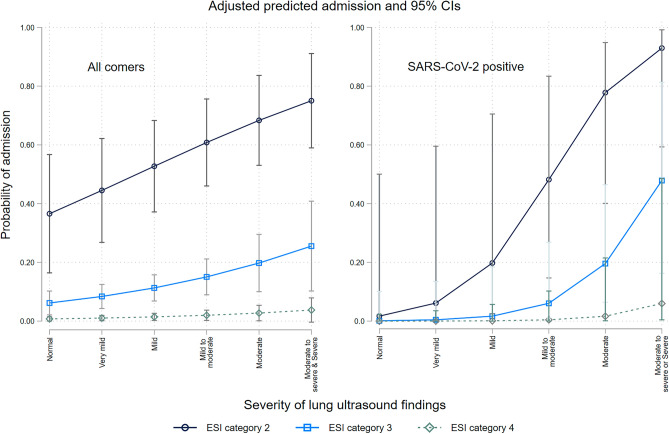
The predicted probability of admission is calculated from the logistic regression model estimating the relationship between the severity of lung ultrasound findings and hospital admission at different triage categories. The whiskers represent 95% confidence intervals. The left panel includes only those patients with a positive SARS-CoV-2 nasal or nasopharyngeal test. ESI, emergency severity index.

Admissions increased with the increasing severity of lung ultrasound findings with the suggestion of a threshold effect at moderate to severe and severe viral pneumonitis (Table 2). The Cochrane-Armitage test for trend was significant (*p* < 0.001) for all comers and SARS-CoV2 positive patients and with evidence of departure from a linear trend (Pearson *p* = 0.001). Admission rates showed similar correlation by triage categories (*p* < 0.0001) with evidence of non-linearity both in SARS-CoV-2 positive and other patients and with oxygen saturation grouped at clinically meaningful levels (*p* = 0.0012). In multivariable-adjusted regression lung ultrasound severity OR 1.52 (95% CI 1.20, 1.94) and Emergency Services Index triage category (OR 0.11 95% CI 0.06, 0.21), were significant predictors of admission. Restricting the analysis to patients who were proven SARS-CoV-2 positive showed a larger point estimate with a very wide CI (OR 3.77, 95% CI 1.32, 10.80). These are shown in Fig. 2. The implication of this figure is of a threshold effect and that lung ultrasound findings are only part of the explanation for hospitalization. Comparing regression models for hospital admission with clinical characteristics (as captured by the Emergency Services Index category (1–5) alone against a model that included these plus the lung ultrasound findings showed better fit characteristics for the model that includes the ultrasound severity). A model with Emergency Services Index category alone had better fit characteristics than a model with lung ultrasound alone.

### Blood tests

Blood tests were obtained in 23% of children in the SARS-CoV-2 positive, negative, and undetermined groups.

### Blood tests and need for hospitalization

**Figure 3:**
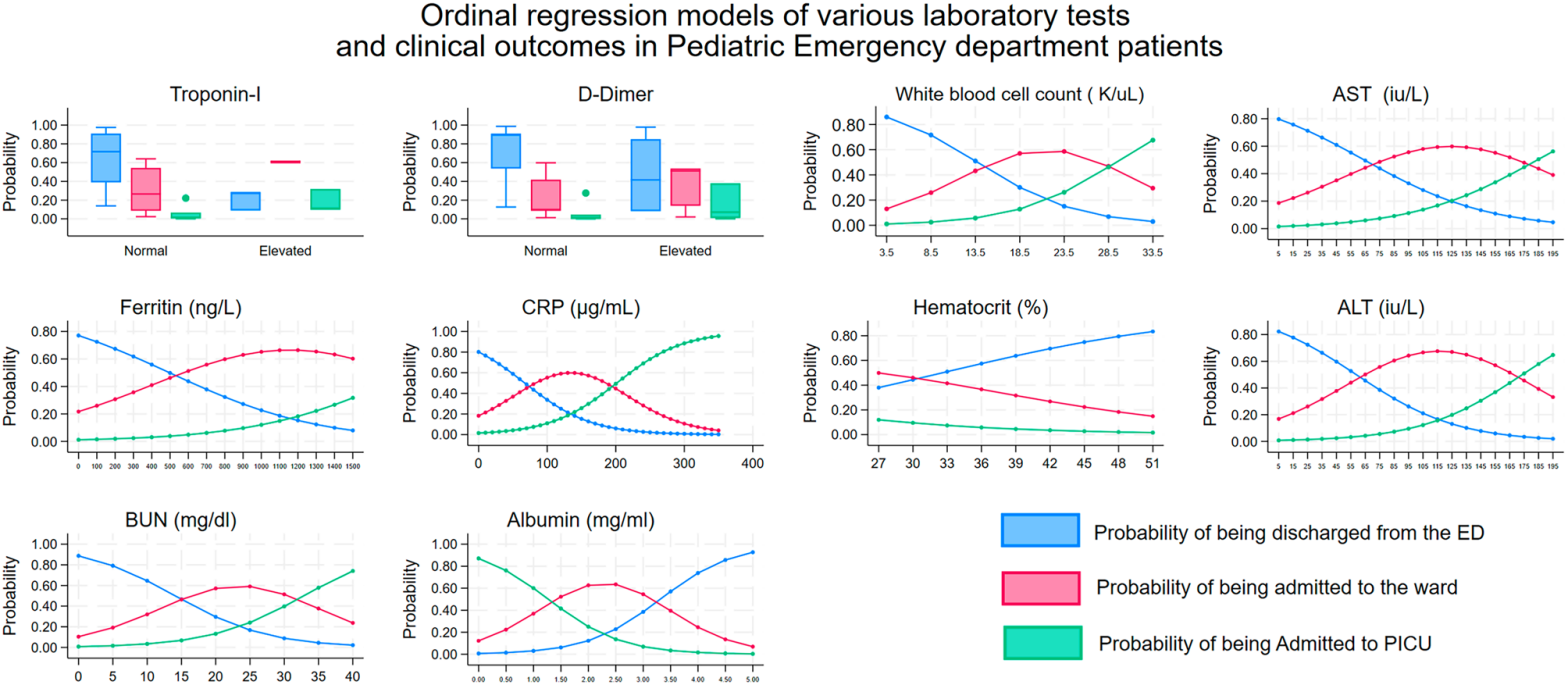
Laboratory findings and disease severity. Plots of the probability of discharge from the ED, admission to the ward, or admission to the PICU for selected laboratory tests. Troponin-I and D-dimer were categorized as positive or negative and are presented as box-plots. The graphs reflect adjustment for ESI triage category. All were statistically significant at the *p* < 0.05 level except for Hematocrit (*p* = 0.067) and AST (*p* = 0.052).

Ordinal logistic regression showed associations between increased odds of admission to the hospital and PICU with increasing levels of BUN, CRP, Ferritin, AST, ALT, and with decreasing levels of Albumin, and Hematocrit. These are shown in Fig. 3. Almost all patients with elevated troponin were admitted to the ward.

### Blood tests that can be predicted from the lung ultrasound

The following blood tests failed the screening process to be included in this part of the analysis: ESR, CRP, procalcitonin, albumin, hematocrit, WBC, Pro-BNP, BUN, creatinine, and troponin-I. These tests could not be confidently skipped even if the lung ultrasound was normal. This was especially true for troponin-I likely a de facto* sine-qua-non* for the diagnosis of viral myocarditis in most pediatric EDs, cardiac societies’ recommendations notwithstanding.

The combined lung ultrasound severity categories of moderate to severe, and severe disease, comprised only 19 (4%) of the lung ultrasounds performed and these were combined into a single group for analysis.

In SARS-CoV-2 positive patients we found a trend between the severity of lung ultrasound findings and AST, ALT, Ferritin, and LDH. Data sparsity limited analysis of elevated D-dimer or troponin. Among all comers, the effect sizes were weaker, although there was sufficient data to analyze D-dimer albeit with very wide confidence intervals. The results of the blood tests for each category of lung ultrasound severity are in Table 3. Using inverse probability weighted linear regression; Ferritin, LDH, AST, and ALT, were significantly associated with the severity of lung ultrasound findings. We had null findings for patients who were SARS-CoV-2 negative. These are shown in the supplements as extended Table 3.

**Table 3:** Laboratory values observed by the severity of lung ultrasound.

Lung ultrasound	Normal	Very mild	Mild	Mild to moderate	Moderate	Moderate-severe & severe	Trend test *p* value
SARS-CoV-2 Positive only							
N	51	19	24	3	14	7	
Ferritin ng/mL	214 (214, 214)	86 (77, 169)	124 (83, 165)	121 (116,125)	336 (40, 881)	585 (558, 675)	0.01
LDH iu/L	298 (298, 298)	238 (202, 273)	314 (203, 424)	(., .)	388 (357, 508)	500 (382, 549)	0.03
ALT iu/L	31 (31, 31)	29 (12, 46)	19 (16, 29)	50 (30, 70)	41 (37, 56)	44 (42, 84)	0.02
AST iu/L	31 (31, 31)	37 (11, 63)	18 (12, 37)	45 (33, 57)	45 (17, 94)	67 (63, 71)	0.03
Albumin mg/ml	3.9 (3.9, 3.9)	4.05 (4, 4.1)	3.95 (3.4, 4.15)	4 (3.9, 4.1)	3.65 (3.5, 3.7)	3.2 (3, 3.5)	0.01
All comers prior to virus testing results being known							
N	200	68	68	30	42	19	
LDH iu/L	306 (253 ,315)	239 (203, 289)	228 (203, 306)	247 (209, 269)	306 (245, 357)	441 (311, 549)	0.05
AST iu/L	26 (17, 33)	25 (16, 46)	18 (16, 35)	23 (16, 30)	21 (19, 56)	56 (42, 65)	0.01
ALT iu/L	26 (17, 31)	43 (18, 55)	17 (8, 25)	21 (15, 57)	32 (16, 54)	64 (41, 71)	0.01
D-dimer (elevated)	3 (43%)	4 (80%)	2 (40%)	2 (29%)	8 (62%)	8 (100%)	0.05

Among all-comers, who reflect the patients prior to SARS-CoV-2 testing, and for whom the emergency physician must decide to obtain (or not obtain) blood tests, the results broadly paralleled the SARS-CoV-2 positive group. The effect size in all-comers was smaller reflecting the dilution of effect by combining children who had SARS-CoV-2 with those who did not, shown in Table 3.

**Figure 4:**
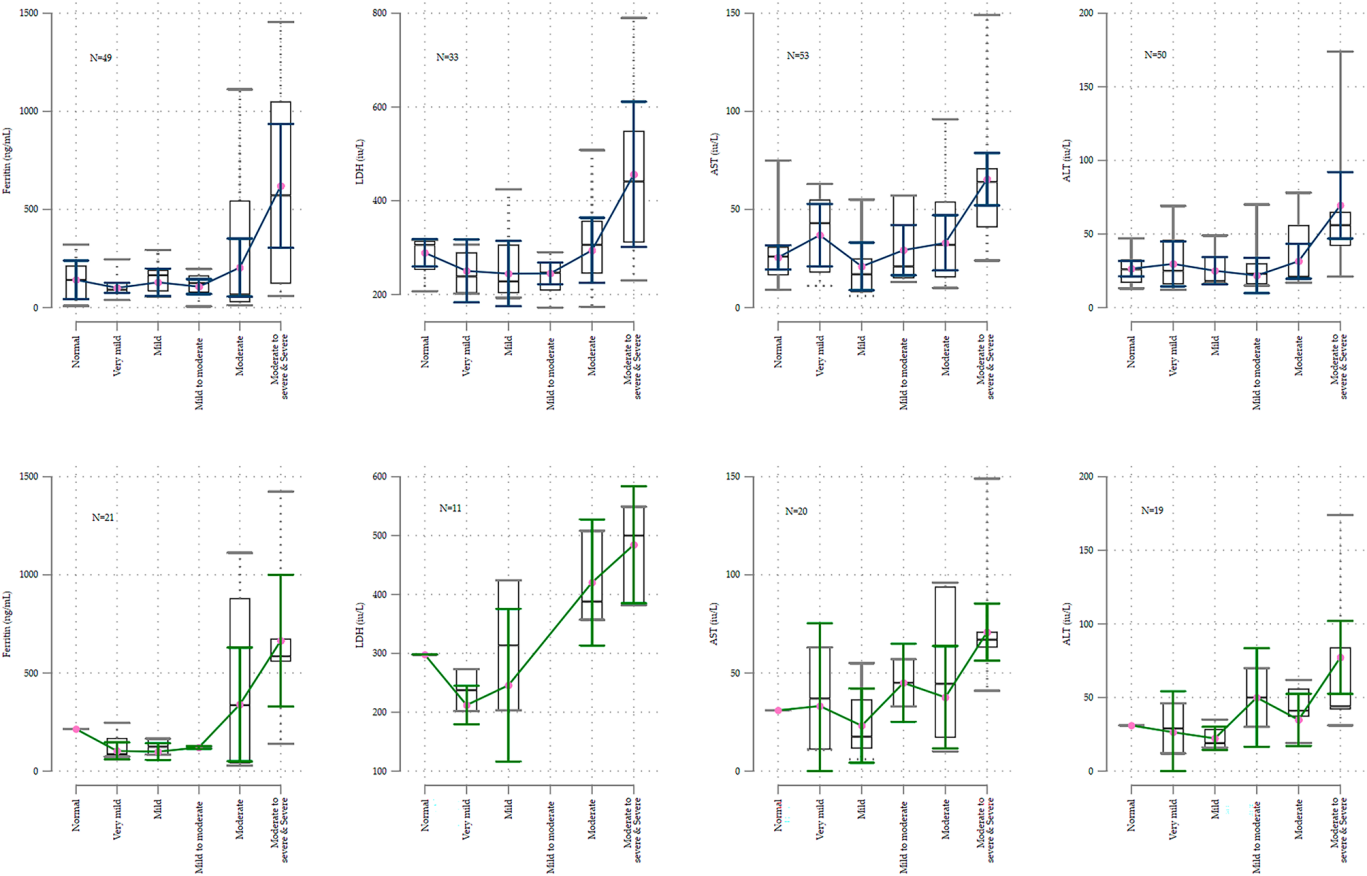
Boxplots of lung ultrasound and disease severity overlaid with inverse probability weighted regression model predicted values. Except for the D-Dimer, important abnormalities in the markers shown are observed only for more than moderately severe lung disease. The top row shows all-comers, and the bottom row: SARS-CoV-2 positive only. AST aspartate aminotransferase, ALT alanine transaminase.

**Figure 5:**
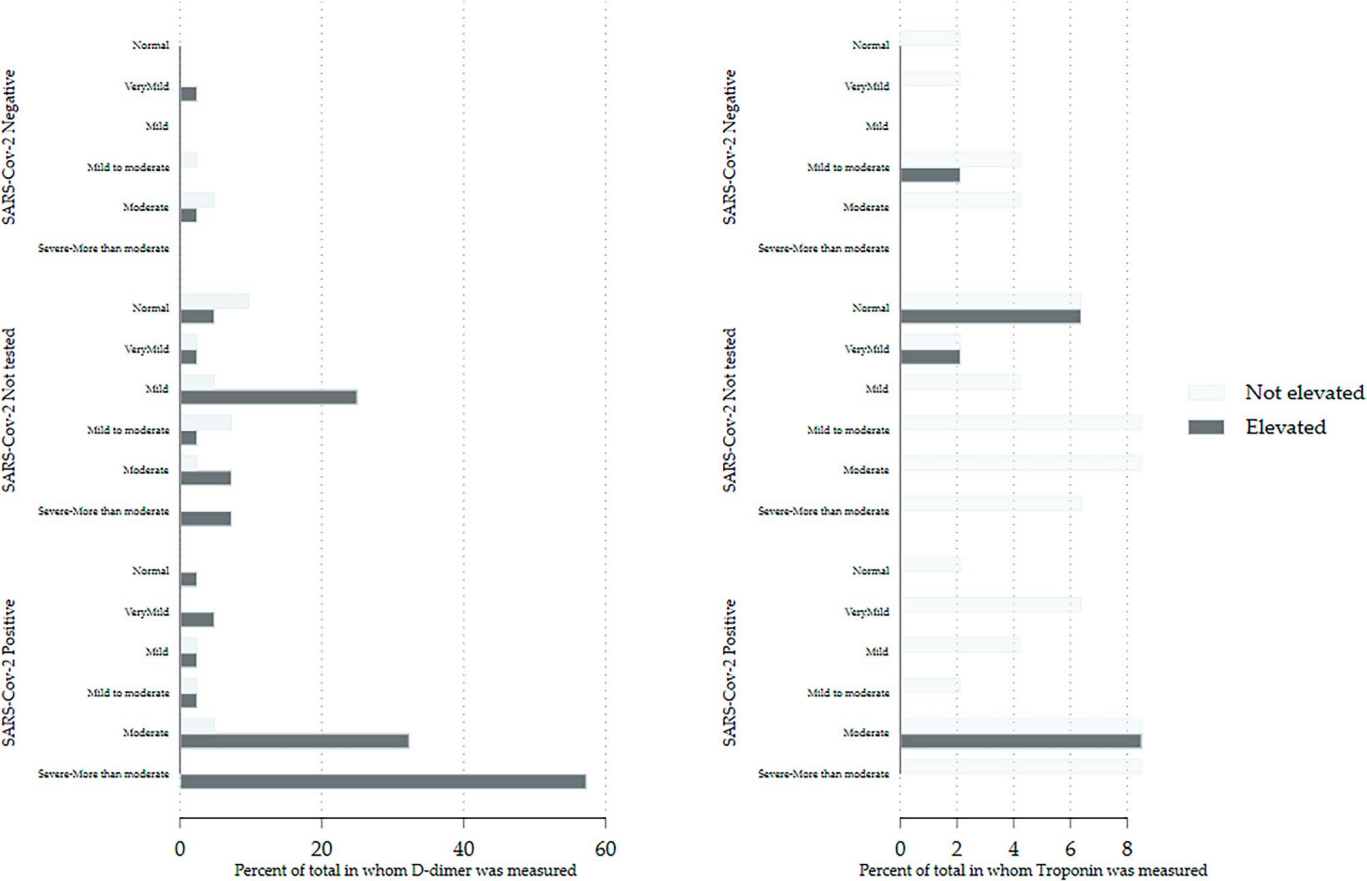
Bar graphs showing elevated D-dimer and troponin-I by SARS-CoV-2 status and severity of lung ultrasound findings.

The process for selecting analyses for Aim 3, determining if lung ultrasound findings were associated with laboratory values, are summarized in Table 4. The raw data for selected blood tests are shown graphically in Fig. 4**.** The results for D-dimer and troponin-I are shown in Fig. 5.

**Table 4:** Models comparing the outcome to lung ultrasound severity.

All comers							
Initial variable selection				Severity of lung ultrasound as categorical The most severe category reported. Normal is referent			
Method		ANOVA	Logit	Unweighted OLS regression		PS derived from all comers PS IPW regression	
		LUS results	LUS results				
Variable	N	F Statistic	β	β	*p* value	β	*p* value
All comers				Moderate to severe & severe LUS			Moderate to severe & severe LUS
Ferritin	49	3.288**	2.89*	481	0.006	367	0.032
LDH	33	2.837**	4.90*	177	0.023	155	0.050
AST	53	3.188**	NC***	40	0.001	35	0.001
ALT	50	3.639**	4.67**	38	0.001	43	0.010
				logit		logit	
				1.33	0.043	1.54	0.050
D-Dimer	43			OR 3.78 (1.05, 13.68)		OR 4.83 (1.00, 23.3)	

SARS-CoV-2 positive patients only Lung ultrasound severity treated as a continuous variable							
		Unweighted OLS regression		Ultrasound treated as continuous			
				PS IPW 1(¶) regression		PS IPW 2 regression	
Variable	N	β	*p* value	β	*p* value	β	*p* value
Ferritin	21	124	0.01	88	0.02	132	< 0.01
LDH	11	48	< 0.01	17	0.39	25	0.13
AST	20	11	0.03	5	0.06	6	0.03
ALT	19	11	0.05	5	0.10	4	0.21

Severity of findings on point of care lung ultrasound Analysis by category of ultrasound severity (uses PS from SARS-CoV-2 positive patients only for balancing)							
Variable	N	Normal	Very mild	Mild	Mild to moderate	Moderate	Moderate to severe & severe
		β (95% CI)	β (95% CI)	β (95% CI)	β (95% CI)	β (95% CI)	β (95% CI)
Ferritin	21	Referent	− 3 (− 96, 90)	Empty cell	8 (− 61, 77)	304 * (− 45, 653)	560 ** (119, 1001)
LDH	11	Referent	− 79 (− 140, − 19)	− 45 (− 226,136)	Empty cell	120 * (− 5, 245)	179 ** (45, 313)
AST	20	Referent	− 6 (− 43, 30)	− 8 (− 26, 10)	14 (− 8, 36)	17 (− 9, 43)	46** (7, 84)
ALT	19	Referent	− 7 (− 36, 21)	− 9 ** (17, − 1)	19 (− 18, 56)	1 (− 19, 21)	42* (− 6, 90)

Laboratory tests had to pass a two-step process on all comers to be included in the final analysis. The first column shows the number of observations in each analysis. The second column shows the results of ANOVA relating ultrasound severity to mean laboratory values for each test. Where the p-value was < 0.10 logistic regression relating lung ultrasound to the laboratory value being elevated was performed. These results are shown in the second column. If this result also had a *p* value of < 0.10 then that particular laboratory test was included in the final analysis. The fourth column shows ordinary least squares regression with laboratory value as the dependent variable and ultrasound severity as the independent variable. Ultrasound severity was treated as a categorical variable with normal as referent. Coefficients (β**)** are shown only for the ’More than moderately severe’ ultrasound group. (The coefficients for lesser degrees of ultrasound severity were generally non-significant.) The fifth column shows the final result; the propensity score derived inverse probability weighted ordinary least squares regression with laboratory value as the dependent variable and ultrasound severity as the independent variable. There were too few cases to allow analysis using categorical outcomes in the SARS-CoV-2-only group. There was no association between any blood test and lung ultrasound severity in the SARS-CoV-2 negative group (data not shown). Screening analysis *p < 0.1, **p < 0.05, ***p < 0.001. Where ALT was reported only as < 6 (n = 3) it was not included in the analysis. Values for albumin ordered as a single add-on test by the admitting service were included in the analysis. D-dimer and troponin were analyzed only as elevated or normal. The full models are in the supplemental materials. (¶)Parsimonious (1) Detailed(2) propensity score models**.** OLS ordinary least squares, LDH lactate dehydrogenase, AST aspartate aminotransferase, ALT alanine transaminase, OR odds ratio, NC not calculable because all were abnormal in more severe ultrasound findings, LUS lung ultrasound, logit logistic regression, PS propensity score, IPW inverse probability weighted.

Table 4 Only significant values are shown. See Supplemental material for full unabridged Table 4 Median (Interquartile range). SARS-CoV-2 Negative and unknown data were all non-significant (data not shown). LDH lactate dehydrogenase, AST aspartate aminotransferase, ALT alanine transaminase, ESR erythrocyte sedimentation rate, CRP c-reactive protein, WBC white blood cell count, Pro-BNP pro-b-natriuretic peptide, OR odds ratio, NC not calculable, LUS lung ultrasound, iu/l international units per liter, K/uL thousands per cubic millimeter, NAAT Nucleic acid amplification test. Rows were removed for all missing data. No data is represented by ‘.(.,.)’. Numbers in brackets represent interquartile range. 

### Differences between younger and older children

In the sensitivity analysis, presented in Table 5**,** we found relatively little effect of age group on our results, although sample size is limited. The results suggest that LDH, AST, ALT, and ferritin tend to be slightly higher in younger children for a given severity of lung ultrasound.

**Table 5:** Sensitivity analysis showing the effect of age on laboratory values based.

	LDH		Ferritin		AST		ALT		D-dimer	
Age group	β	p	β	p	β	p	β	p	OR	p
Neonate	51	0.248	51	0.002	20	0.003	1	0.935	0.15	0.258
Infant 1–12 months	108	0.035	16	0.874	33	0.001	20	0.001	0.11	0.272
Preschooler 1–5 years	21	0.594	− 35	0.709	15	0.006	14	0.015	0.21	0.381
School-age 6–12 years	Ref	–	Ref	–	Ref	–	Ref	–	Ref	–
Teenager 13–18 years	− 47	0.153	− 17	0.773	6	0.416	8	0.283	0.08	0.083
Transition 18–21 years	− 8	0.859	− 17	0.825	6	0.475	6	0.461	0.25	0.395

Each column shows the results of an inverse probability weighted logistic regression to see if age group affects the relationship between ultrasound findings and laboratory values. The beta coefficient reflects how the laboratory value would be modified for each age group for a given blood test. These results are not corrected for multiple comparisons. These models had poorer fit characteristics than the simpler regressions used in our main analysis. OR odds ratio, **β** regression coefficients, p p-value, Ref referent.

## Discussion

Increasingly severe pneumonitis on lung ultrasound was associated with increased admission rates from the ED. This was true regardless of the specific virus. The severity of lung ultrasound findings alone did not fully explain the decision to admit.

Increasingly abnormal laboratory values predicted worsening clinical outcomes as measured by decreased ED discharges, increased ward admissions, and increased PICU admissions.

A normal lung ultrasound would have allowed the emergency physician to forgo obtaining Ferritin, LDH, and LFT transaminases, but not troponin, creatinine, BUN, Pro-BNP; ESR, CRP, D-Dimer, or procalcitonin. Moderate and less severe lung ultrasound abnormalities could have been tolerated without missing elevations in Ferritin, LDH, and elevated LFT transaminases.

Our finding that increasingly severe lung damage as demonstrated on bedside lung ultrasound is associated with an increased probability of hospitalization is unsurprising. It is consistent with CT findings in adult practice and with other investigators findings in children. Our findings that increasingly abnormal laboratory values predict worse outcomes are also broadly consistent with other studies. Similar associations to those we found have been described for lung ultrasound findings and ferritin levels^26,27^ and lung CT and ferritin and D-dimer levels^28^. The consideration of Covid-19 as a new hyperferritinemic syndrome^15^ has arisen from the observation that children who die have respiratory and hyperferritinemic manifestations of Covid and their postmortem lung histology shows elevated ferritin. Our observation that lung damage may occur without hyperferritinemia is consistent with CT and pathological studies in adults^29^. Even in the presence of more severe lung pathology patients with a good prognosis have normal ferritin levels in contrast to those who do badly who have both severe lung pathology and are hyperferritinemic.

We had hoped because Covid-19 has been primarily a lung disease, that the detailed information provided by lung ultrasound would be sufficient to allow stratification of these patients’ care. There is evidence for this in adults where univariate analysis shows associations between laboratory values and lung ultrasound severity^25^. This turned out to not be the case in our data: inflammatory changes appear more systemic, not all of the hypercoagulablity is occurring in the lungs, and myocarditis could be present even when the lungs appeared unscathed. Where our results differ from others may reflect a difference in how we modeled the data: rather than using linear regression between lung ultrasound scores and laboratory values we observed and then modeled a threshold effect.

Operationalizing our results requires the incorporation of information from other research. First, we believe lung ultrasound is essential to the accurate management of these children without excessive radiation^4,30–35^. A practice model that uses auscultation to screen who gets further investigation is limited by the stethoscope’s 8% sensitivity to detect Covid pneumonitis. Even that 8% included older more cooperative children^1^. Creating a pathway that includes only those with auscultatory findings would miss an unacceptable number of patients who require more care. Chest X-ray performance is similarly dismal missing 75% of cases in one study, and having a sensitivity of 25% in another^1,36^.

Second, the decision to obtain blood tests or admit the child to the hospital does not depend solely on the severity of lung findings on imaging. If a child has more than moderately severe pneumonitis on lung ultrasound, then ferritin and other markers of severe disease should be obtained. For lesser degrees of lung ultrasound abnormalities, the decision to obtain laboratory testing will rest on other clinical factors such as chest pain or irritability, and in these cases, testing should then focus on troponin and systemic inflammatory markers. D-dimer results reflect both pulmonary and peripheral clotting and the decision to test rests on the philosophy of treating physicians regarding the management of pulmonary and disseminated hypercoagulable states in children.

The ability of lung ultrasound to help triage blood tests is useful when treating infants and toddlers in whom blood is especially difficult to obtain and in whom choices about which tests can be run must often be made. Blood testing children is resource intensive. A physician can complete and document a point-of-care lung ultrasound in less than 10 min, but obtaining blood from a small child can easily take two nurses 20 min. The blood samples then have to be transported to the lab and processed. And then the physician has to review the lab results. If lung ultrasound could obviate the need for any blood testing the impact of our work would have been greater.

In some patients where there is a concern for bacterial pneumonia, combining lung ultrasound with blood tests such as procalcitonin will likely be the optimal strategy^37^.

### Conceptual model of Covid-19

Our work supports a conceptual model of Covid-19 that goes beyond respiratory disease. In this conceptual model of Covid-19, although disease severity ranges from minimal symptoms to progressively more severe lung disease, the most severe form of Covid comprises severe lung disease plus a syndrome similar to macrophage activation syndrome (also termed secondary haemophagocytic lymphohistiocytosis)^20^. This model, derived in adults, contemplates elevated ALT and AST in tandem with elevated ferritin (typically > 500 ng/ml^38^) and more severe lung injury^20,39^. This is what we observed here in our pediatric data also.

Omicron variants of SARS-CoV-2 have helped clarify the extra-pulmonary pathogenicity of Covid-19 in general. Accordingly, clinicians and healthcare planners must think beyond overly simplistic oxygen saturation thresholds as the only admission criterion as could occur if SARS-CoV-2 infection is characterized as requiring admission only if it causes hypoxia. Instead, management decisions must consider Covid-19 as having overlapping pulmonary, hematological, clotting, immunological, gastrointestinal, and cardiac components any one of which can lead to morbidity.

Myocarditis was identified early as a cause of death in adult patients and although death is rare in children, myocarditis does occur. In our data, troponin elevations above the 99th centile were observed in 9/48 (19%) patients and were not related to lung ultrasound severity. Although not formally measured in our review, the decision to obtain troponin testing appears to have been guided by the appearance of irritability in preverbal and chest pain in verbal children. Unlike adults, elevated troponin in children does not usually reflect ischemia or renal failure, and from the ED perspective has to be managed as reflecting myocarditis^23,40^. ECGs are insufficiently sensitive to rule out myocarditis^40^. A formal diagnosis of myocarditis requires a tissue diagnosis, immunohistological staining, and extensive serum antibody testing^41^. A practical working diagnosis of myocarditis for pediatric emergency medicine is one of irritability, diaphoresis with feeding, chest pain, palpitations, new heart murmur, hepatomegaly, edema, or cardiomegaly in combination with either a suggestive ECG, or CXR, or an elevated troponin. Using this framework, it is likely that all of these elevated troponins reflected myocarditis. Our findings are inconvenient for pediatric emergency practice; in an irritable infant with SARS-CoV-2 infection, even in the absence of lung involvement, troponin testing may still be needed.

**Figure 6:**
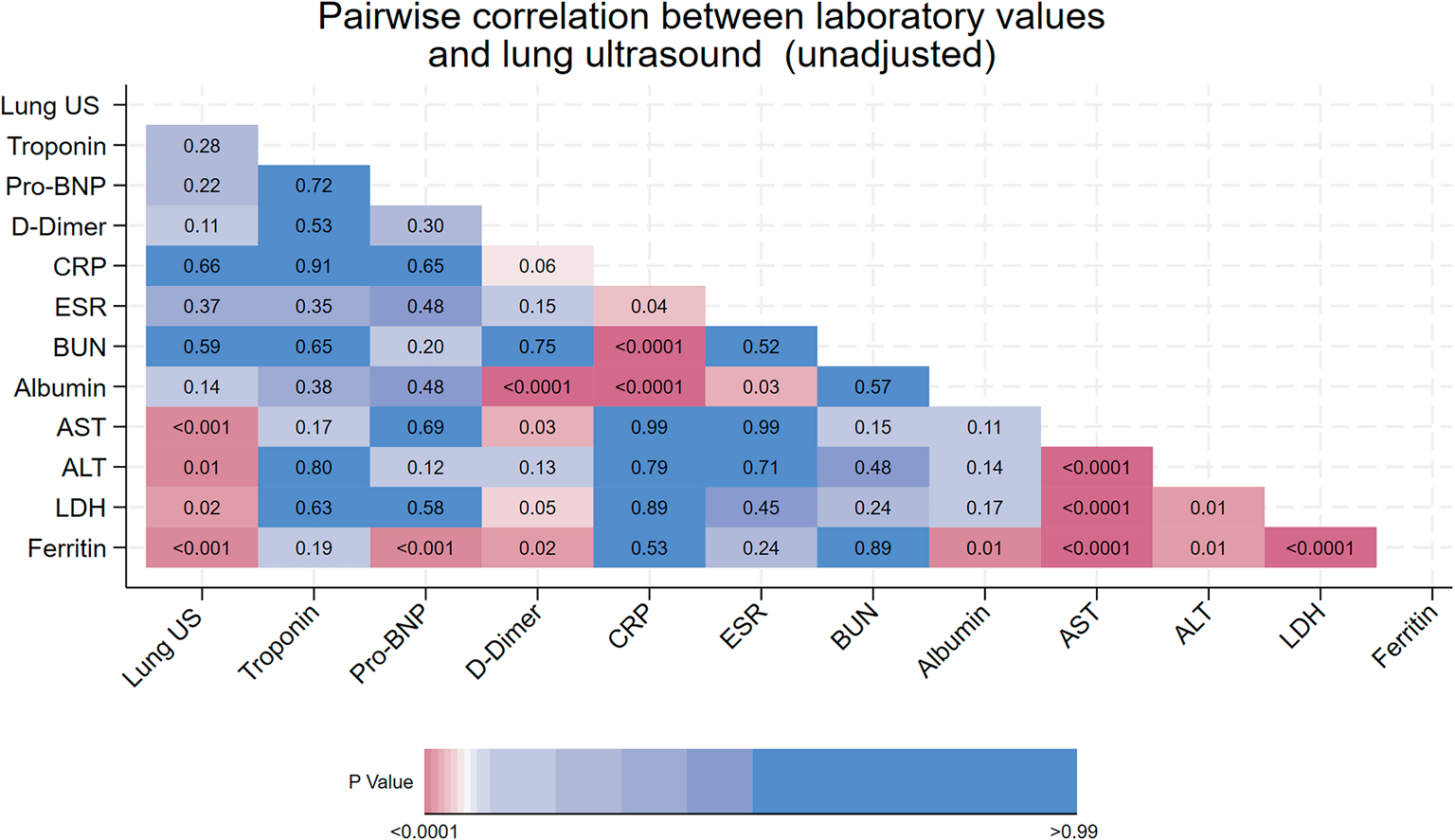
Raw pairwise correlations between laboratory tests.

It is tempting to simplify our findings by organ system, transaminases for liver function, troponin for myocarditis and so on. However, the interpretation of many laboratory tests is contextual on others. An elevated Pro-BNP may indicate heart failure in the context of an elevated troponin but pulmonary hypertension in the context of isolated severe lung disease. And while each of elevated transaminases and LDH raise concern for worse outcomes, the most feared complication, a Covid induced hyperferritinemic state is typically associated with elevations in these other enzymes too. Figure 6 below demonstrates this pattern.

Our finding that D-dimer was universally elevated in those children with the most severe lung disease but could also be elevated in those without lung disease provides support for the concept of distinct processes of lung-centric pulmonary intravascular coagulation^20^ and peripheral sepsis-induced disseminated intravascular coagulation^42^ that could occur separately or simultaneously. Our finding however is sharply limited by the small numbers in whom D-dimer was obtained and by the necessity of dichotomization of the results of different assays.

In short, imaging the lungs informs the clinician about the lungs. A conceptual model of Covid-19 disease where a hyperferritinemic state with its associated laboratory abnormalities, morbidity, and mortality complicates some, but not all, patients with more severe lung disease is emerging. Our findings dovetail with this conceptual model. Lung ultrasound does not inform the clinician about the presence of myocarditis or peripheral inflammation.

### Limitations

Our sample size is small, non-random, and reflects the experiences of a single community pediatric ED. This is particularly the case for subgroups. We treated each visit rather than the patient (371/427) as the unit of analysis. Blood sampling was performed at the discretion of the treating clinician and most children did not have any blood testing performed. We addressed this by first deriving propensity score models to predict the likelihood of a given child having a particular blood test ordered. We then used inverse probability weighting to adjust the next step in the analysis. To prevent false discoveries we implemented a rigorous two step screening process to limit which blood tests would qualify for inclusion in the final regressions.

The assays used for D-Dimer and troponin were changed during the study period. We addressed this by analyzing D-dimer and troponin as elevated or normal. This created a binary instead of a continuous variable and taken with already small sample sizes risks sparse data problems for these variables.

The sample includes patients from a period when SARS-CoV-2 testing was not available in community practice. This is reflected in the results where the presence of laboratory abnormalities typically associated with Covid-19 is noted with some frequency in the ‘SARS-CoV-2 positive’ group, less frequently in the ‘SARS-CoV-2 untested’ group, and less frequently again in the SARS-CoV-2 negative group. This untested group also includes patients whose parents either refused viral testing outright or permitted only non-SARS-CoV-2 viral testing to be performed (to avoid their child being restricted from school etc.). We addressed this by repeating the analyses across these groups.

Our testing was limited to PCR testing on nasal and nasopharyngeal swabs without any quantitative information. Given the airborne transmission of SARS-CoV-2 some false negatives, especially at initial presentation seem likely and some patients with pneumonitis on lung ultrasound likely had SARS-CoV-2 infection, particularly when no alternative virus was detected.

Viral testing may for some centers be something of a moving target. Point-of-care PCR has been demonstrated to improve ED care for RSV and influenza^43^. Antigen tests in general have significant performance problems unless the prevalence is high^44,45^. In EDs with point-of-care PCR machines that allow a triage nurse to perform the test and have a result available when the physician sees the patient, the subgroup analysis of SARS-Cov-2 results should be used rather than all-comers.

We had a small number of ultrasound operators. The ultrasounds did not always clearly indicate a reported severity of findings, consequently, we dropped 73/500 (15%) visits from the analysis. This helps ensure consistency of the scans performed and their interpretation but potentially at the price of generalizability. We did not use a numeric ultrasound scoring system. Numeric scoring systems are inherently attractive, particularly for statisticians, when developing prospective research. However, numeric scoring systems are time-consuming to use, leading to fewer study subjects being enrolled, and their apparent precision is often illusory. One widely cited scoring schema designed for Covid-19 involves calculating a specific score for 14 areas and increasing the score by one point (on scale from 0 to 3) when consolidation is present in an area^46^. This differs from our method of scanning where we slide the probe down the back and chest wall to avoid missing small areas, and our method of reporting where consolidation is specifically noted and separately described in the ultrasound report. This may introduce more subjectivity than the former approach and it led to us being unable to include almost 15% of scans in our analysis. We found evidence of such illusory precision in our own grading; even in the absence of a numeric scoring system; the ultrasonographers in this study interpreted far more categories of severity of lung ultrasound than objectively matter when correlated with laboratory data or hospital admission. We, like others, have found consolidation to be much less common in SARS-Cov-2 infections than other respiratory infections^47^.

Our sample includes a broad age range of patients. We addressed this with a sensitivity analysis to answer the question ‘Did age group matter?’ This method allowed us to avoid the problem of subdividing our sample into many smaller groups each of which contains insufficient data to be individually informative and may prove useful in pediatric research in general. Our sensitivity analyses suggest that LDH, AST, ALT, and ferritin tend to be slightly higher in infants and younger children compared to older children for a given severity of lung ultrasound; although the magnitude was small. These findings are consistent with the findings of a pooled analysis of studies of laboratory abnormalities and disease severity in children with Covid-19^17^.

## Conclusion

In summary, lung ultrasound informs mostly about the lungs; lung pathology alone did not fully account for the hospitalization of children with viral pneumonitis in general and Covid-19 in particular. The answers to our three questions are:

1. The severity of lung ultrasound abnormalities does, to a degree, predict hospitalization.
2. A normal lung ultrasound does not obviate the need to obtain troponin, CRP, or D-dimer levels if these are otherwise indicated but the other blood tests studied may be avoided.
3. There is a threshold effect between lung ultrasound abnormalities and blood abnormalities. Moderate to severe, and severe lung ultrasound abnormalities are associated with hyperferritinemia, elevated LDH, elevated transaminases, and an elevated D-dimer when the SARS-CoV2 was positive.

## Methods

The study was approved by Sutter Health institutional review board. All methods were carried out in accordance with relevant guidelines and regulations. Informed consent for study participation was waived by the Sutter Health institutional review and Sutter Health privacy boards.

This was a retrospective chart review. Patients were eligible if they were being evaluated for possible SARS-CoV-2 infection, were aged between 14 days and 21 years of age, were seen in our ED, and had a point-of-care lung ultrasound reported in the chart. Data collection for this study includes data from 30/November/2019 to 14/August/2021. Patient visits for multi-system inflammatory syndrome in children following Covid-19 were excluded. This date range included early periods in the pandemic when testing was not available or severely rationed, and even earlier periods where SARS-Co-2 infection would be expected to be unlikely. We included these periods in our ‘all-comers’ group to provide more conservative estimates than would be obtained by limiting our sample to the period of peak prevalence.

### Outcomes

Our outcome for the questions “does lung ultrasound predict outcomes?” was hospital admission versus discharge from the ED. Our outcome for ultrasound was severity of lung ultrasound findings on a six-point scale. Our outcomes for blood tests were association between the results and PICU, versus ward admission, versus discharge from the ED.

### Data sources

All data was obtained from the electronic medical record. We used the reference ranges provided by the laboratory for each patient. For procalcitonin, > 0.50 ng/mL was considered abnormal. Only laboratory values drawn during the ED visit were included.

### Viral testing

Nasal and nasopharyngeal swabs were collected using nylon flocked swabs in universal transport medium. Nucleic acid testing was performed using DiaSorin Molecular Simplexa COVID-19 Direct real-time RT-PCR assay (DiaSorin Molecular LLC, Cypress, California, USA) or Roche Cobas SARS-CoV-2 6800/8800 Systems real-time RT-PCR assay (Roche Molecular Systems Inc., Branchburg, New Jersey, USA). PCR cycle thresholds were not reported. Antigen testing was not used. Blood tests and respiratory pathogen panels were performed as part of usual clinical care in the hospital laboratory.

### Chart abstraction

Chart selection and extraction were performed electronically using regexm functions rather than ICD-10 codes. We did this because ICD codes applied at discharge from clinical settings do not necessarily capture the clinical diagnosis^48^. This arises because physicians sometimes hand type the diagnosis in the discharge diagnosis field when they have trouble retrieving their preferred diagnosis in the electronic health care user interface drop-down lists. The SQL terms used in the diagnosis/chief complaint field were (dx_name like '%viral pneumonitis%' or dx_name like '%covid%' or dx_name like '%sars-cov%' ordx_name like '%anosmia%' or dx_name like '%loss of smell%' or dx_name like '%dysgeusia%' ordx_name like '%loss of taste%' or dx_name like 'altered mental%' or dx_name like '%confus%' ordx_name like '%myalgia%' or dx_name like '%muscle ache%' or dx_name like '%neck ache%' ordx_name like '%asthma%' or dx_name like '%cough%' or dx_name like '%short of breath%' ordx_name like '%dyspnea%' or dx_name like '%weakness%' or dx_name like '%guillain%' or dx_name like '%myocarditis%' or dx_name like '%fever%'). These charts were then screened for point-of-care lung ultrasound reports using regexm functions. Lung ultrasound reports were extracted using natural language string parsing. Charts were directly reviewed in Epic for validation and clarification as needed.

**Figure 7:**
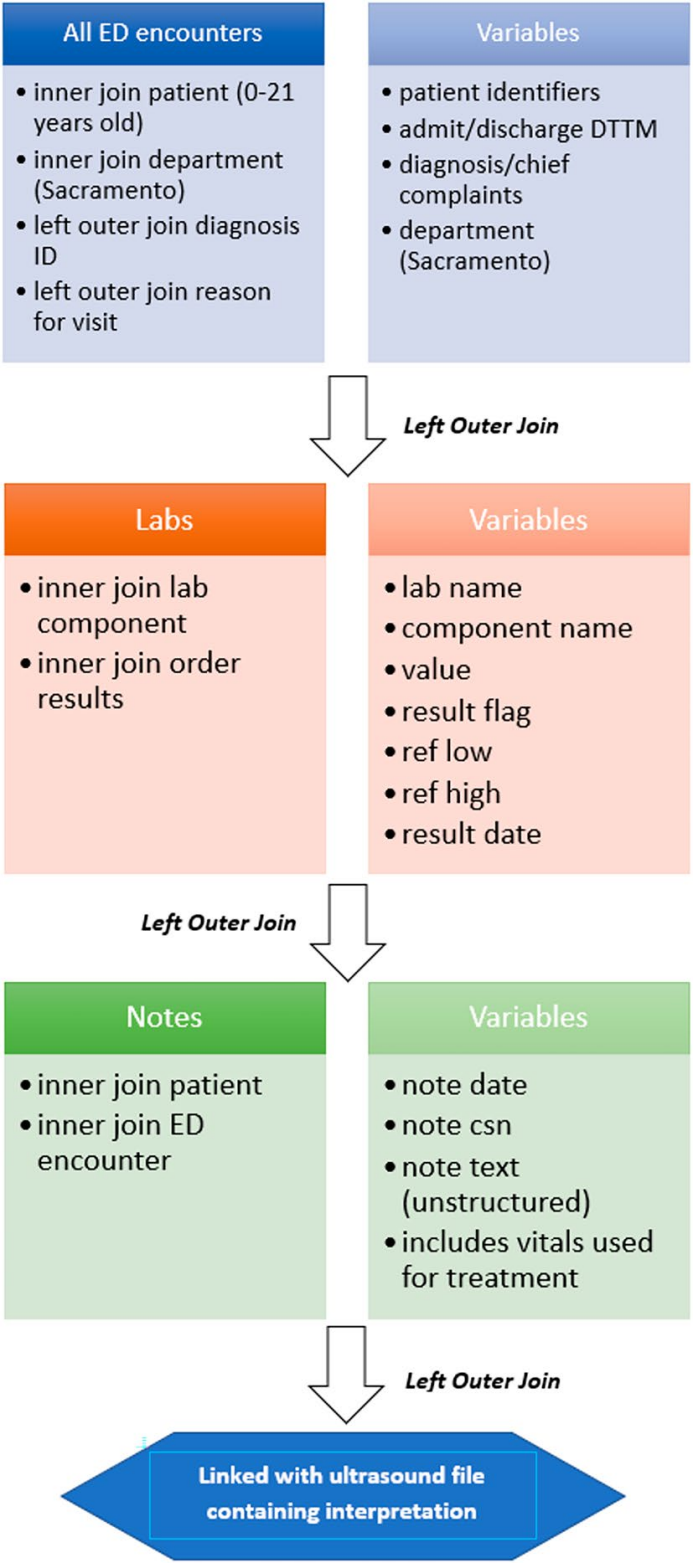
SQL extraction from the electronic medical record.

The SQL code used to extract laboratory data is diagrammed in Fig. 7.

### Ultrasound

Ultrasounds were performed by general or pediatric emergency physicians or emergency medicine residents. Most were performed by pediatric emergency physicians who had each performed over 100 lung ultrasounds on a bovine model of bronchiolitis/respiratory disease complex prior to the Covid-19 pandemic.

One author (PW) reviewed all the lung ultrasound reports using a template to assign severity. The ultrasound reviewer was not blinded to the study questions. Ultrasound reports were extracted as text snippets and presented to the reviewer without access to the remainder of the electronic medical record. The reviewer categorized each ultrasound using a 7-point scale: normal, or as having very mild, mild, mild to moderate, moderate, moderate to severe, or severe pneumonitis.

The ultrasonographers' determinations of lung ultrasound severity were somewhat subjective. They did not use a formalized score based on the counting of B-lines in specific acoustic access points (which would be difficult to do in real-time in often uncooperative infants and toddlers). That said we found them to be remarkably consistent. The online version includes representative video clips and reports. Still images from these are shown.

We performed the Kruskal–Wallis test of the hypothesis that the ultrasound classifications performed by different ultrasonographers followed the same distribution (implying they are drawn from the same population). We also performed ordinal logistic regression with ultrasound classification as the dependent and the ultrasongrapher and triage category as the independent variables. The ultrasonographers were identified as one of two pediatric emergency physicians, resident physicians, or general emergency physicians.

Because of the small numbers in the ‘moderate to severe’ and ‘severe’ categories, these were combined into one category in the analysis phase. Ultrasound reports rather than raw images were used for two reasons. First, in community practice bedside point-of-care lung ultrasounds frequently include only representative images rather than clips of every window. This is especially true in highly mobile and uncooperative infants and toddlers. Second, the written ultrasound report allows us to know how the physician who decided whether or not to obtain blood tests or admit the child interpreted the findings. Representative samples of ultrasound video clips are included.

### Statistical analysis

We used a variety of methods to ensure consistency and robustness in our results. We addressed the potential selection bias for obtaining laboratory studies with propensity score analysis. We limited our analysis to those in whom lung ultrasound was performed. We present the results for all-comers, and separately for those in whom nasal or nasopharyngeal NAAT testing was positive and negative. Some patients were seen before such testing was available and the airborne nature of SARS-CoV-2 means that lung infection without nasal infection occurs in some patients.

### Intra-rater reliability of lung ultrasound report interpretation

Each report was presented multiple times in a slightly different text fragment without access to the rest of the medical record. Where different determinations of lung ultrasound severity were made the most severe category was used in the analysis.

A subset of 71 patients had their final ultrasound report re-evaluated by the original reviewer blinded to the original report. This re-evaluation occurred more than one month after the original evaluations were performed. The intra-rater agreement was measured using Gwet’s AC-1 coefficient and intraclass correlation coefficient (random-effects model). These are preferred to κ statistics when prevalence and agreement are expected to be high^49–51^. Intra-rater reliability was measured using the original seven classifications of disease severity.

### Relationship between lung ultrasound findings and hospital admission

We performed the Cochran-Armitage test for a trend of the severity of lung ultrasound findings and hospitalization. We also performed this test for oxygen saturation (measured at triage) and the initial Emergency Severity Index (ESI) triage category. Oxygen saturation was modeled both using a dichotomous threshold of 92%, using oxygen saturation grouped as follows, 97–100%; 94–96%; 92–93%; 91–90%, < 90%, and < 85%, and as a continuous variable. We included ultrasound severity, oxygen saturation, and the ESI^52^ triage category in multivariable-adjusted analysis using logistic regression. Ultrasound severity (0–6) and ESI triage category (0–5) were modeled as continuous. We observed multicollinearity between the ESI triage category and oxygen saturation. Models with ESI category and ultrasound severity had better fit characteristics than those with oxygen saturation or both ESI category and oxygen saturation. We also compared regression models of hospital admission with clinical characteristics (as captured by the ESI category (1–5) alone versus a model that included ESI category plus lung ultrasound using Bayesian information criteria.

### Relationship between blood tests and clinical outcomes

Our outcome measures were Discharge from the ED, Ward admission, and PICU admission. We performed ordinal logistic regression with the dependent variable ordered as Discharge from the ED, Ward admission, and PICU admission. We adjusted all analyses for ESI triage category. These models had better fit and specification characteristics than models without ESI triage category and provided a necessary adjustment when disentangling clinical characteristics from laboratory ones. We used the omodel program in Stata to test the proportional odds assumption. We used marginsplot with ESI triage category set at 3 to graph the results of these models.

### Relationship between lung ultrasound and laboratory findings

The decision to obtain laboratory investigations on these children suspected of having Covid-19 was non-random and was made before the results from nasal PCR swabs were available. We performed ANOVA tests with the value of the blood test as the dependent and lung ultrasound severity as the independent variable. We also performed logistic regression dichotomizing the lung ultrasound severity categories as being above or below the highest two levels of severity. We performed the Jonckheere–Terpstra test for a trend for each lab test result for ultrasound severity. Any blood test with a *p* value < 0.10 in both the ANOVA and logistic regression among all-comers was further analyzed by propensity score analysis^53–55^. The variable screening procedure was based on all-comers with SARS-CoV-2 positive and negative patients subsequently being analyzed as subgroups.

### Propensity score analysis

We estimated inverse probability weights for each of these blood tests using logistic regression-based propensity score analysis. These scores included the ultrasound findings; ESI triage category; and for some blood tests admission; with the precise specification depending on the fit characteristics of the model. These inverse probability weights were then used to weight ordinary least squares regression analysis. This was done to account for the non-random decision to obtain blood tests which were likely influenced by the overall severity of the illness of the child (reflected in the ESI category) and the severity of the lung ultrasound findings. Logistic regression was used because the decision to obtain blood work is ultimately binary, even if the factors leading to that decision are not.

In the case of troponin and D-dimer, both assays were changed during the study period and no calibration between the two tests was provided. Therefore, we dichotomized each at the 99th centile and analyzed these as normal or abnormal using logistic regression. In the case of D-dimer, dichotomizing the lung ultrasound severity categories as being moderately severe was necessary to avoid perfect prediction of elevated d-dimer in the presence of ‘moderate to severe and severe’ lung ultrasound findings. An alternative analysis using exact logistic regression which allows for this scenario is in the appendices. We used Stata17.1 (StataCorp LLP, College Station, TX) for data analyses.

### Sensitivity analysis

We did a sensitivity analysis to address the range of developmental and physiological phases encompassed by pediatrics. We repeated our final models for each significant laboratory test but included age group as a dummy variable using school-age children as the referent category. We did this because of the broad range of ages of our patients. The age groups we used were: Neonate 14–28 days, infant, 29 days to 1 year; preschooler, 1–5 years; school-age, 6–12 years, teenager, 13–18 years; and transitional, 18–21 years. We excluded neonates younger than 14 days because of the potential for residual pulmonary hypertension making lung ultrasound interpretation more difficult. Our pediatric ED sees all children covered by California Children’s Service (CCS) up to age 21 years of age, and depending on capacity may also see non-CCS-covered children up to 21 years of age. Because of the small numbers and the potential for false negative results, we report these results only for all-comers. We analyzed age group rather than age because the effects of age on physiology and development are non-linear and these categories, although uneven, better express the stages of childhood. These analyses answered the question: Are there differences between age groups in these outcomes?

### Supplementary Information

Supplementary Information 1.

Supplementary Information 2.

Supplementary Information 3.

Supplementary Information 4.

Supplementary Information 5.

Supplementary Information 6.

Supplementary Information 7.

Supplementary Information 8.

Supplementary Information 9.

Supplementary Information 10.

Supplementary Information 11.

Supplementary Video 1.

Supplementary Video 2.

Supplementary Video 3.

Supplementary Video 4.

Supplementary Video 5.

Supplementary Video 6.

Supplementary Video 7.

## Data Availability

A de-identified data set along with the statistical code needed to reproduce our analysis is included in the supplementary materials.
