# Supplementary material for: Point-of-care lung ultrasound predicts hyperferritinemia and hospitalization, but not elevated troponin in SARS-CoV-2 viral pneumonitis in children
Source: Sci Rep. 2024 Mar 11;14:5899. doi: 10.1038/s41598-024-55590-9 (PMC10928070; doi:10.1038/s41598-024-55590-9)
Supplement: Supplementary file 1 — Supplementary Information 1. [file 41598_2024_55590_MOESM1_ESM.docx]

**Supplemental Information files**

S1. Table. Respiratory panel

S2. Statistical code (Aims 1 &3)

S3. Extended Table 3

S4. Extended Table 4

S5. Sample of US reports and their classification

S6. Dataset. De-identified data set xls format

S7. Dataset. De-identified data set Stata format

S8. Dataset to create the left-sided panel in Figure 1

S9. Statistical code (Aim 2)

Supplementary file type: Representative video clips (numbered 1-7)

Supplementary File Type

Representative video clips (numbered 1-7)

Clip 1 Normal on lung ultrasound

Clip 2 Very mild disease on lung ultrasound

Clip 3 Mild disease on lung ultrasound

Clip 4 Mild to moderate disease on lung ultrasound

Clip 5 Moderate disease on lung ultrasound

Clip 6 Moderate to severe disease on lung ultrasound

Clip 7 Severe disease on lung ultrasound
