# Supplementary material for: Point-of-care lung ultrasound predicts hyperferritinemia and hospitalization, but not elevated troponin in SARS-CoV-2 viral pneumonitis in children
Source: Sci Rep. 2024 Mar 11;14:5899. doi: 10.1038/s41598-024-55590-9 (PMC10928070; doi:10.1038/s41598-024-55590-9)
Supplement: Supplementary file 2 — Supplementary Information 2. [file 41598_2024_55590_MOESM2_ESM.rtf]

013f
Virus Full results				Freq.		Percent		Cum.
			
CORONAVIRUS HKU1			2		0.93			0.93
CORONAVIRUS NL63			5		2.31			3.24
CORONAVIRUS OC43			3		1.39			4.63
CORONAVIRUS 229E			2		0.93			5.56
RHINOVIRUS/ENTEROVIRUS	          73		33.80			39.35
INFLUENZA A				4		1.85			41.20
INFLUENZA B				1		0.46			41.67
PARAINFLUENZA 1 VIRUS		1		0.46			42.13
PARAINFLUENZA 3 VIRUS		6		2.78			44.91
CHLAMYDOPHILA PNEUMONIAE	1		0.46			45.37
SARS_CoV2-sutterlab		        108	 	50.00			95.37
SARS_CoV2-S/C-DPH lab		          10		4.63			100.00
			
Total					        216		100.00
Normal;heading 1;heading 2;heading 3;heading 4;heading 5;heading 6;heading 7;heading 8;heading 9;caption;Title;Subtitle;Strong;Emphasis;Placeholder Text;No Spacing;Light Shading;Light List;Light Grid;Medium Shading 1;Medium Shading 2;Medium List 1;Medium List 2;Medium Grid 1;Medium Grid 2;Medium Grid 3;Dark List;Colorful Shading;Colorful List;Colorful Grid;Light Shading Accent 1;Light List Accent 1;Light Grid Accent 1;Medium Shading 1 Accent 1;Medium Shading 2 Accent 1;Medium List 1 Accent 1;Revision;List Paragraph;Quote;Intense Quote;Medium List 2 Accent 1;Medium Grid 1 Accent 1;Medium Grid 2 Accent 1;Medium Grid 3 Accent 1;Dark List Accent 1;Colorful Shading Accent 1;Colorful List Accent 1;Colorful Grid Accent 1;Light Shading Accent 2;Light List Accent 2;Light Grid Accent 2;Medium Shading 1 Accent 2;Medium Shading 2 Accent 2;Medium List 1 Accent 2;Medium List 2 Accent 2;Medium Grid 1 Accent 2;Medium Grid 2 Accent 2;Medium Grid 3 Accent 2;Dark List Accent 2;Colorful Shading Accent 2;Colorful List Accent 2;Colorful Grid Accent 2;Light Shading Accent 3;Light List Accent 3;Light Grid Accent 3;Medium Shading 1 Accent 3;Medium Shading 2 Accent 3;Medium List 1 Accent 3;Medium List 2 Accent 3;Medium Grid 1 Accent 3;Medium Grid 2 Accent 3;Medium Grid 3 Accent 3;Dark List Accent 3;Colorful Shading Accent 3;Colorful List Accent 3;Colorful Grid Accent 3;Light Shading Accent 4;Light List Accent 4;Light Grid Accent 4;Medium Shading 1 Accent 4;Medium Shading 2 Accent 4;Medium List 1 Accent 4;Medium List 2 Accent 4;Medium Grid 1 Accent 4;Medium Grid 2 Accent 4;Medium Grid 3 Accent 4;Dark List Accent 4;Colorful Shading Accent 4;Colorful List Accent 4;Colorful Grid Accent 4;Light Shading Accent 5;Light List Accent 5;Light Grid Accent 5;Medium Shading 1 Accent 5;Medium Shading 2 Accent 5;Medium List 1 Accent 5;Medium List 2 Accent 5;Medium Grid 1 Accent 5;Medium Grid 2 Accent 5;Medium Grid 3 Accent 5;Dark List Accent 5;Colorful Shading Accent 5;Colorful List Accent 5;Colorful Grid Accent 5;Light Shading Accent 6;Light List Accent 6;Light Grid Accent 6;Medium Shading 1 Accent 6;Medium Shading 2 Accent 6;Medium List 1 Accent 6;Medium List 2 Accent 6;Medium Grid 1 Accent 6;Medium Grid 2 Accent 6;Medium Grid 3 Accent 6;Dark List Accent 6;Colorful Shading Accent 6;Colorful List Accent 6;Colorful Grid Accent 6;Subtle Emphasis;Intense Emphasis;Subtle Reference;Intense Reference;Book Title;Bibliography;TOC Heading;
