# Supplementary material for: Point-of-care lung ultrasound predicts hyperferritinemia and hospitalization, but not elevated troponin in SARS-CoV-2 viral pneumonitis in children
Source: Sci Rep. 2024 Mar 11;14:5899. doi: 10.1038/s41598-024-55590-9 (PMC10928070; doi:10.1038/s41598-024-55590-9)
Supplement: Supplementary file 3 — Supplementary Information 3. [file 41598_2024_55590_MOESM3_ESM.pdf]

```
//For Heejung
///

// 20230109

**# Bookmark #1
cap drop covid3
gen covid3 =.
 lab var covid3 "Pos Covid by NAAt/PCR swab/bronch missing =."
  replace covid3 =1 if covid_pos ==1
  replace covid3 =1 if covid_pos2 ==1
  replace covid3 =1 if covid_confirm    =="1"
  replace covid3 =1 if nasal_covid ==1
  replace covid3 =1 if  viral_panel2 ==42
   replace covid3 =0 if  covid3 ==.  & viral_panel2 <. & viral_panel2 !=42
   replace covid3 =0 if  covid3 ==.  & viral_panel ==7  & viral_panel2 !=42

cap drop covid4
gen covid4 =covid3
 replace covid4 =0 if covid3==.
  lab var covid4 "Pos Covid by NAAt/PCR swab/bronch missing =0"



///Revisions for PLOS one
// Create a variable for PCR proven SARS-CoV2
//

 **# Propensity score for


 // PC Crashed re-written form spreadsheet records


**# Bookmark #3
//ferritn parsimonious
 foreach var in ferritin_measure {
    cap drop  iptwt_`var'
     logit `var' i.f_score i.triage_imp o.admit
     estat gof
     cap drop propensity
      predict propensity ,pr

      propwt ferritin_measure propensity, ipt gen(wt_`var')

      //reg l_ferritin i.f_score

     // reg l_ferritin i.f_score [pweight =iptwt_`var']

       reg l_ferritin i.f_score if covid3==1

      reg l_ferritin i.f_score [pweight =iptwt_`var']  if covid3==1
        reg l_ferritin f_score [pweight =iptwt_`var']  if covid3==1
 }
    xi: pbalchk  ferritin_measure   i.f_score  i.triage_imp sp02  ,wt(iptwt_ferritin_measure  ) graph



//ferritin detailed

cap drop reg_l_fer_norm
cap drop reg_l_fer_propwt
 foreach var in ferritin_measure {
```

```
     cap drop  iptwt_`var'

      logit `var' i.f_score i.triage_imp   sp02 age_y
      estat gof
      cap drop propensity
       predict propensity ,pr

       propwt ferritin_measure propensity, ipt gen(wt_`var')

       reg l_ferritin i.f_score
          predict reg_l_fer_norm ,xb


       reg l_ferritin i.f_score [pweight =iptwt_`var'] if covid3==1 ,nocon
          predict reg_l_fer_propwt ,xb
 }
  tw (lowess reg_l_fer_norm f_score )(sc l_ferritin  f_score ) (lowess reg_l_fer_propwt  f_score ) ( lowess l_ferritin  f_score )

    xi: pbalchk  ferritin_measure   i.f_score  i.triage_imp sp02  ,wt(iptwt_ferritin_measure  ) graph

  //AST Parsimonious
    cap drop ast_done
 gen ast_done =1 if l_lft_ast <.
 replace ast_done =0 if l_lft_ast ==.

       foreach var in ast_done {
     cap drop  iptwt_`var'
      logit `var' i.f_score##c.triage_imp o.admit
      estat gof
      cap drop propensity
       predict propensity ,pr

       propwt ast_done  propensity, ipt gen(wt_`var')

       reg l_lft_ast i.f_score

       reg l_lft_ast  i.f_score [pweight =iptwt_`var']
 }
    xi: pbalchk  ast_done   i.f_score i.triage_imp   sp02  ,wt(iptwt_ast_done  ) graph






 //AST detailed

 cap drop ast_done
 gen ast_done =1 if l_lft_ast <.
 replace ast_done =0 if l_lft_ast ==.

       foreach var in ast_done {
     cap drop  iptwt_`var'
      logit `var' i.f_score c.f_score i.triage_imp sp02 c.age_y##c.triage_imp
      estat gof
      cap drop propensity
       predict propensity ,pr

       propwt ast_done  propensity, ipt gen(wt_`var')

       reg l_lft_ast i.f_score

```

```
        reg l_lft_ast  i.f_score [pweight =iptwt_`var']
    }

     xi: pbalchk  ast_done   i.f_score  i.triage_imp sp02  ,wt(iptwt_ast_done  ) graph

   //ALT Parsiominious
     cap drop alt_done
    gen alt_done =1 if l_lft_alt <.
    replace alt_done =0 if l_lft_alt ==.

        foreach var in alt_done {
      cap drop  iptwt_`var'
       logit `var' i.f_score##c.triage_imp i.admit
       estat gof
       cap drop propensity
        predict propensity ,pr

        propwt alt_done propensity, ipt gen(wt_`var')

        reg l_lft_alt i.f_score

        reg l_lft_alt  i.f_score [pweight =iptwt_`var']
    }

    xi: pbalchk  alt_done  i.f_score i.triage_imp   sp02  ,wt(iptwt_alt_done  ) graph


   //ALT Detailed
    cap gen age_y = age_m /12 added in after safe harbor deleted this from the file
     cap drop alt_doneA
    gen alt_doneA =1 if l_lft_alt <.
    replace alt_doneA =0 if l_lft_alt ==.

        foreach var in alt_doneA {
      cap drop  iptwt_`var'
       logit `var' i.f_score##c.triage_imp   sp02 c.age_y##c.triage_imp
       estat gof
       cap drop propensity
        predict propensity ,pr

        propwt alt_doneA propensity, ipt gen(wt_`var')

        reg l_lft_alt i.f_score

        reg l_lft_alt  i.f_score [pweight =iptwt_`var']
    }
    xi: pbalchk  alt_doneA   i.f_score i.triage_imp    sp02  ,wt(iptwt_alt_doneA  ) graph




       //parsimonious
            foreach var in ldh_done {
      cap drop  iptwt_`var'
       logit `var'   i.f_score i.triage_imp

       estat gof
       cap drop propensity
        predict propensity ,pr

        propwt ldh_done propensity, ipt gen(wt_`var')

        reg l_ldh i.f_score
```

```
        reg l_ldh  i.f_score [pweight =iptwt_`var']
    }
    xi: pbalchk  ldh_done   i.f_score  i.triage_imp sp02  ,wt(iptwt_ldh_done  ) graph

      //detailed

        foreach var in ldh_done {
      cap drop  iptwt_`var'
       logit `var'   i.f_score i.triage_imp    sp02 c.age_y c.triage_imp

       estat gof
       cap drop propensity
        predict propensity ,pr

        propwt ldh_done propensity, ipt gen(wt_`var')

        reg l_ldh i.f_score

        reg l_ldh  i.f_score [pweight =iptwt_`var']
    }
     xi: pbalchk  ldh_done   i.f_score  i.triage_imp sp02  ,wt(iptwt_ldh_done  ) graph



      foreach var in dimer_measured {
     cap drop iptwt_`var'
       logit `var'   i.f_score i.triage_imp pts_that_hourP21


        estat gof
       cap drop propensity
        predict propensity ,pr

        propwt dimer_measured propensity, ipt gen(wt_`var')

       logit elev_dimer f456, or

       logit elev_dimer f456 i.age_group ,or

       logit elev_dimer f456 [pw =iptwt_`var'] ,or

       logit elev_dimer f456 i.age_group1 [pw =iptwt_`var']  ,or
    }
    xi: pbalchk  dimer_measured  i.f_score i.triage_imp sp02  ,wt(iptwt_dimer_measured  ) graph
     // The issue here is that F56 has perfect predcition for 8 values. P values are very unstable and can get well below 0.05 using exlogisitc and manually generated weighted outcomes. But, F456 avoids perfect prediction and seems to proved estimates that appeart more reasonable.
   //detailed
       foreach var in dimer_measured {
     cap drop iptwt_`var'
       logit `var' i.f_score i.triage_imp##sp02 c.age_y c.triage_imp pts_that_hourP14



        estat gof
       cap drop propensity
        predict propensity ,pr

        propwt dimer_measured propensity, ipt gen(wt_`var')

       logit elev_dimer f456, or

```

```
    logit elev_dimer f456 i.age_group1 ,or

     logit   elev_dimer f456 [pw =iptwt_`var'] ,or

     logit elev_dimer f456 i.age_group1 [pw =iptwt_`var']  ,or
 }

 xi: pbalchk  dimer_measured   i.f_score i.triage_imp  sp02  ,wt(iptwt_dimer_measured  ) graph

 **## Table 3  Raw lab data for each category

// See spreadsheet for code
 loc flagD "if flagD==0"

 table1   ,by(f_score) vars(l_crp conts\  l_esr  conts\l_procalcitonin conts\ l_ferritin  conts\ l_ldh conts\ l_lft_alt conts\ l_lft_ast conts\ l_alb  conts\ l_hematocrit  conts\ l_cbc_wbc conts\ l_abs_lymphocyte conts\ l_abs_neutrophil conts\  l_bnp conts\ l_creatinine  conts\ elev_troponin bine \elev_dimer bine) saving(table_3b_flagD_revised_a.xlsx ,replace)


//  Table 3 test of trend column
foreach var in l_crp l_esr l_procalcitonin l_ferritin l_ldh l_lft_ast l_lft_alt l_alb l_hematocrit l_cbc_wbc  l_abs_lymphocyte l_abs_neutrophil  l_bnp l_creatinine    {
    di "`var'"

    nptrend `var' `flagD',gr(f_score) jterpstra
}


 nptrend   elev_dimer , gr(f_score) carmitage
 nptrend   elev_troponin , gr(f_score) carmitage



//l_crp l_abs_lymphocyte l_abs_neutrophil  l_bicarb l_bnp l_cbc_wbc l_dimer_new l_dimer_old l_esr l_ferritin l_hematocrit l_ldh l_lft_ast l_lft_alt l_procalcitonin
 //table_1mc allows total column

// Table 3b Include only Covid POS by nasal swab

table1  if covid3 ==1  ,by(f_score) vars(l_crp conts\  l_esr  conts\l_procalcitonin conts\ l_ferritin  conts\ l_ldh conts\ l_lft_alt conts\ l_lft_ast conts\ l_alb  conts\ l_hematocrit  conts\ l_cbc_wbc conts\      l_bnp conts\ l_creatinine  conts\ elev_troponin bine \elev_dimer bine) saving( table_3b_flagD_revised_b.xlsx ,replace sheet(Covid_POS_only))

 foreach var in l_crp l_esr l_procalcitonin l_ferritin l_ldh l_lft_ast l_lft_alt l_alb l_hematocrit l_cbc_wbc   l_bnp l_creatinine   elev_troponin elev_dimer    {

    nptrend `var' if covid3==1 ,gr(f_score)     jterpstra

    }
 nptrend   elev_dimer  if covid3==1, gr(f_score) carmitage
 nptrend   elev_troponin if covid3==1 , gr(f_score) carmitage
 //

 table1  if covid3 ==0  ,by(f_score) vars(l_crp conts\  l_esr  conts\l_procalcitonin conts\ l_ferritin  conts\ l_ldh conts\ l_lft_alt conts\ l_lft_ast conts\ l_alb  conts\ l_hematocrit  conts\ l_cbc_wbc conts\      l_bnp conts\ l_creatinine  conts\ elev_troponin bine \elev_dimer bine) saving( table_3b_NEG_COVID_revised_c.xlsx ,replace sheet(Covid_NEG_only))

 foreach var in l_crp l_esr l_procalcitonin l_ferritin l_ldh l_lft_ast l_lft_alt l_alb l_hematocrit l_cbc_wbc   l_bnp l_creatinine     {

```

```
    di "`var'"
    di  `var'

nptrend `var' if covid3==0 ,gr(f_score)  jterpstra

}


 nptrend   elev_dimer , gr(f_score) carmitage
 nptrend   elev_troponin , gr(f_score) carmitage










 **# Figure 2 Graph (components first)

  gr box l_ldh if covid3==1 ,over(f_score )
    gr play ldh_graph.grec
     graph save "Graph" "ldh_graph_rev.gph"

   gr box l_ferritin if covid3==1 ,over(f_score )
    // manual clean up in editor
     graph save "Graph" "ferritin_graph_rev.gph"

    gr box l_lft_ast l_lft_alt if covid3==1,over(f_score )
    //manual clean up
    graph save "Graph" "AST_ALT_graph_rev.gph", replace

   stop need bar graph for dimer
    graph save "Graph" "dimer_graph_rev.gph"
      graph save "Graph" "dimer_graph_rev.gph", replace

    //////////

    //Create a marker for US experience by operator
    //

    ///

**#   Used in seprate do file to pull ot the Sp02 then merged back in.
/*
cap drop t1
cap drop t2
cap drop t3
cap drop t4
cap drop t5
cap drop t6
cap drop t7

gen t1 =strpos(note_text, "Sp02")

gen t3 = substr(note_text ,t1 ,35)

gen t4 =(strpos(t3, "%")  -4)
gen t5 =substr(t3,t4,4)

gen t6 = .
replace t6 =100 if regexm(t5, "100")
```

```
replace t6 =99 if regexm(t5 ,"99")
replace t6 =98 if regexm(t5 ,"98")
replace t6 =97 if regexm(t5 ,"97")
replace t6 =96 if regexm(t5 ,"96")
replace t6 =95 if regexm(t5 ,"95")
replace t6 =94 if regexm(t5 ,"94")
replace t6 =93 if regexm(t5 ,"93")
replace t6 =92 if regexm(t5 ,"92")
replace t6 =91 if regexm(t5 ,"91")
replace t6 =90 if regexm(t5 ,"90")
replace t6 =89 if regexm(t5 ,"89")
replace t6 =88 if regexm(t5 ,"88")
replace t6 =87 if regexm(t5 ,"87")
replace t6 =86 if regexm(t5 ,"86")
replace t6 =85 if regexm(t5 ,"85")
replace t6 =84 if regexm(t5 ,"84")
replace t6 =83 if regexm(t5 ,"83")
replace t6 =82 if regexm(t5 ,"82")
replace t6 =81 if regexm(t5 ,"81")
replace t6 =80 if regexm(t5 ,"80")

replace t6 =.a if regexm(t5, "/=")
replace t6 =.b if regexm(t3, "Min:")
replace t6 =.b if regexm(t3, "Max")

    */



**# Sp02 Groupings
cap drop gr_sp02
cap lab drop o2
cap lab drop ox

gen gr_sp02 =1 if sp02 >=97
replace gr_sp02 =2 if sp02 <97 & sp02>=94
replace gr_sp02 =3 if sp02 <94 & sp02>=92
replace gr_sp02 =4 if sp02 <92 & sp02>=90
replace gr_sp02=5 if sp02 <90
replace gr_sp02 =6 if sp02 <=85

lab define ox 1 "sp02 >=97" 2 "sp02 94%-96%"  3 "Sp02 92%-94%" 4 "Sp02 91%-90%" 5 "Sp02 <90%"  6 "Sp02 <85%"

lab val gr_sp02 o2

loc flagD "if flagD==0"

// Paragraph 3 Results  Admission
nptrend admit `flagD'  ,gr(f_score ) carmitage
nptrend admit `flagD' ,gr(triage_imp ) carmitage
nptrend admit `flagD' ,gr(gr_sp02  ) carmitage

logit admit  f_score  triage_imp `flagD' ,or
margins ,at(f_score =(0/5))
marginsplot


//

//

// Table 3  Test of trend column
```

```
//
foreach var in l_crp l_esr l_procalcitonin l_ferritin l_ldh l_lft_ast l_lft_alt l_alb l_hematocrit l_cbc_wbc  l_abs_lymphocyte l_abs_neutrophil  l_bnp l_creatinine   elev_troponin elev_dimer    {
    di "`var'"

    nptrend `var' `flagD',gr(f_score) jterpstra
}

//Uses T16 after dropping (7) those cases identified by flagD in T15 for bing in the study before 11-30-19
//
//Table 5 data

tab any_lab
tab wbc_done
tab crp_done
tab esr_done
tab bnp_done
reg l_ldh  i.f_score i.age_group1  [pweight =iptwt_ldh_done ]
reg l_ldh  i.f_score  [pweight =iptwt_ldh_done ]
reg l_ldh  i.f_score i.b4.age_gr [pweight =iptwt_ldh_done ]
reg l_ldh  f4 i.b4.age_gr [pweight =iptwt_ldh_done ]
reg l_ldh  i.f_score i.b4.age_gr [pweight =iptwt_ldh_done ]
reg l_ferritin  i.f_score i.b4.age_gr [pweight =iptwt_ferritin_measure  ]
reg l_lft_ast   i.f_score i.b4.age_gr [pweight =iptwt_ast_done  ]
reg l_lft_alt   i.f_score i.b4.age_gr [pweight =iptwt_alt_done  ]
logistic  elev_dimer   i.f_score i.b4.age_gr [pweight =iptwt_dimer_measured  ]
logit elev_dimer f456 i.age_group [pw =iptwt_dimer_measured ]  ,or
    //
//
//
//


cap frame drop diagt_f456
frame create diagt_f456  str15(variable) double(ppv lb_ppv ub_ppv npv lb_npv ub_npv sens  lb_sens ub_sens spec lb_spec ub_spec auc auc_lb auc_ub prev)
 foreach var in admit elev_ferritin elev_ldh elev_lft_alt elev_lft_ast elev_crp elev_esr elev_procalcitonin   elev_dimer elev_trop {
    di "`var'"
    diagt `var' f456 if flagD==0,sf

    frame post diagt_f456   ("`var'") (r(ppv)) (r(ppv_lb)) (r(ppv_ub)) (r(npv)) (r(npv_lb)) (r(npv_ub)) ( r(sens)) (r(sens_lb)) (r(sens_ub)) (r(spec))  (r(spec_lb)) (r(spec_ub)) (r(roc))  (r(roc_lb))  (r(roc_ub)) (r(prev))


}
frame diagt_f456 :save diagt_f456 ,replace



cap frame drop diagt_f56
frame create diagt_f56  str15(variable) double(ppv lb_ppv ub_ppv npv lb_npv ub_npv sens  lb_sens ub_sens spec lb_spec ub_spec auc  auc_lb auc_ub prev)
 foreach var in admit elev_ferritin elev_ldh elev_lft_alt elev_lft_ast elev_crp elev_esr elev_procalcitonin   elev_dimer elev_trop {
    di "`var'"
    diagt `var' f56 if flagD==0,sf

    frame post diagt_f56   ("`var'") (r(ppv)) (r(ppv_lb)) (r(ppv_ub)) (r(npv)) (r(npv_lb)) (r(npv_ub)) ( r(sens)) (r(sens_lb)) (r(sens_ub)) (r(spec))  (r(spec_lb)) (r(spec_ub)) (r(roc)) (r(roc_lb))  (r(roc_ub))(r(prev))
```

```
}
frame diagt_f56 :save diagt_f56 ,replace




///revised Table 1
**# Bookmark #2
 di _N

 tab covid3

 tab male
 tab male  if covid3==1
 tab male  if covid3==0
 tab male  if covid3==.

 tabstat age_y, stat(mean sd  p50 p25 p75)
 tabstat age_y  if covid3==1,  stat(mean sd  p50 p25 p75)
 tabstat age_y  if covid3==0,  stat(mean sd  p50 p25 p75)
  tabstat age_y  if covid3==.,  stat(mean sd  p50 p25 p75)

 tab age_group1
 tab age_group1 if covid3==1
  tab age_group1 if covid3==0
 tab age_group1 if covid3==.

   tab age_group1 if covid3==1

 tab triage_imp
 tab triage_imp if covid3==1
tab triage_imp if covid3==0
tab triage_imp if covid3==.


 tabstat ed_los ,stat(p50 p25 p75)
 tabstat ed_los if covid3==1 ,stat(p50 p25 p75)
tabstat ed_los if covid3==0 ,stat(p50 p25 p75)
tabstat ed_los if covid3==. ,stat(p50 p25 p75)

 tabstat ed_los if admit ==0 ,stat(p50 p25 p75)
 tabstat ed_los if admit ==0 &  covid3==1 ,stat(p50 p25 p75)
    tabstat ed_los if admit ==0 &  covid3==0 ,stat(p50 p25 p75)
    tabstat ed_los if admit ==0 &  covid3==. ,stat(p50 p25 p75)

    codebook BloodLabResulted
    codebook BloodLabResulted   if covid3==1
    codebook BloodLabResulted   if covid3==0
    codebook BloodLabResulted   if covid3==.
    di 118-91
di 27/118
di 211-162
di 49/211
di 19/98
di 98-76
di 22/98
di 427-329
di 98/427

tab admit if covid3 ==1
tab admit if covid3 ==0
```

```
tab admit if covid3 ==.



// Can't rely on initial number must subtract because some blood tests reported at exact same time

 tab year
 tab year if covid3==1
  tab year if covid3==0
  tab year if covid3==.



//Table 2  Revised

tab f_score if covid3 ==1
tab f_score if covid3 ==1 & admit ==1 ,mis

tab f_score if covid3 ==0
tab f_score if covid3 ==.

 tab f_score if covid3 ==0 & admit ==1 ,mis
 tab f_score if covid3 ==. & admit ==1 ,mis

 /////////////////////////
 //////////////////////////
**# Bookmark Table 4 Logit column to include on COVID POS SWAB


 // ANOVA
 // Column 1 and 2
 //
 //Revised table 4
 // Logits for column 3 only !!!!!

 // OLS  Column 4 &5
 // Adjusted OLS   ^& 7


//ferritn parsimonious
 foreach var in ferritin_measure {
    cap drop  iptwt_`var'
     logit `var' i.f_score i.triage_imp o.admit
     estat gof
     cap drop propensity
      predict propensity ,pr

      propwt ferritin_measure propensity, ipt gen(wt_`var')

      //reg l_ferritin i.f_score

     // reg l_ferritin i.f_score [pweight =iptwt_`var']

       reg l_ferritin i.f_score if covid3==1

      reg l_ferritin i.f_score [pweight =iptwt_`var']  if covid3==1
        reg l_ferritin f_score [pweight =iptwt_`var']  if covid3==1
 }
    xi: pbalchk  ferritin_measure   i.f_score  i.triage_imp sp02  ,wt(iptwt_ferritin_measure  ) graph



//ferritin detailed
```

```

cap drop reg_l_fer_norm
cap drop reg_l_fer_propwt
 foreach var in ferritin_measure {
    cap drop  iptwt_`var'

     logit `var' i.f_score i.triage_imp   sp02 age_y
     estat gof
     cap drop propensity
      predict propensity ,pr

      propwt ferritin_measure propensity, ipt gen(wt_`var')

      reg l_ferritin i.f_score  if covid3==1
         predict reg_l_fer_norm ,xb



      reg l_ferritin i.f_score [pweight =iptwt_`var'] if covid3==1
         predict reg_l_fer_propwt ,xb
 }
  tw (lowess reg_l_fer_norm f_score )(sc l_ferritin  f_score ) (lowess reg_l_fer_propwt  f_score ) (
lowess l_ferritin  f_score )

   xi: pbalchk  ferritin_measure   i.f_score  i.triage_imp sp02  ,wt(iptwt_ferritin_measure  ) graph

  //AST Parsimonious
   cap drop ast_done
 gen ast_done =1 if l_lft_ast <.
 replace ast_done =0 if l_lft_ast ==.

      foreach var in ast_done {
    cap drop  iptwt_`var'
     logit `var' i.f_score##triage_imp c.admit
     estat gof
     cap drop propensity
      predict propensity ,pr

      propwt ast_done  propensity, ipt gen(wt_`var')

      reg l_lft_ast i.f_score if covid3 ==1

      reg l_lft_ast i.f_score [pweight =iptwt_`var']  if covid3==1
 }
 xi: pbalchk  ast_done i.f_score i.triage_imp sp02  ,wt(iptwt_ast_done) graph






 //AST detailed

 cap drop ast_done
 gen ast_done =1 if l_lft_ast <.
 replace ast_done =0 if l_lft_ast ==.

      foreach var in ast_done {
    cap drop  iptwt_`var'
     logit `var' i.f_score c.f_score i.triage_imp sp02 c.age_y##triage_imp
     estat gof
     cap drop propensity
      predict propensity ,pr
```

```

      propwt ast_done  propensity, ipt gen(wt_`var')

      reg l_lft_ast i.f_score

      reg l_lft_ast  i.f_score [pweight =iptwt_`var']
  }

   xi: pbalchk  ast_done   i.f_score  i.triage_imp sp02  ,wt(iptwt_ast_done  ) graph

 //ALT Parsiominious
   cap drop alt_done
  gen alt_done =1 if l_lft_alt <.
  replace alt_done =0 if l_lft_alt ==.

      foreach var in alt_done {
    cap drop  iptwt_`var'
     logit `var' i.f_score##c.triage_imp i.admit
     estat gof
     cap drop propensity
      predict propensity ,pr

      propwt alt_done propensity, ipt gen(wt_`var')

      reg l_lft_alt i.f_score

      reg l_lft_alt  i.f_score [pweight =iptwt_`var']
  }

  xi: pbalchk  alt_done  i.f_score i.triage_imp   sp02  ,wt(iptwt_alt_done  ) graph


 //ALT Detailed

   cap drop alt_doneA
  gen alt_doneA =1 if l_lft_alt <.
  replace alt_doneA =0 if l_lft_alt ==.

      foreach var in alt_doneA {
    cap drop  iptwt_`var'
     logit `var' i.f_score##c.triage_imp   sp02 c.age_y##c.triage_imp
     estat gof
     cap drop propensity
      predict propensity ,pr

      propwt alt_doneA propensity, ipt gen(wt_`var')

      reg l_lft_alt i.f_score if covid3==1

      reg l_lft_alt  i.f_score [pweight =iptwt_`var']  if covid3==1
  }
  xi: pbalchk  alt_doneA   i.f_score i.triage_imp    sp02  ,wt(iptwt_alt_doneA  ) graph



   //LDH
     //parsimonious
          foreach var in ldh_done {
    cap drop  iptwt_`var'
     logit `var'   i.f_score i.triage_imp

     estat gof
     cap drop propensity
```

```
        predict propensity ,pr

        propwt ldh_done propensity, ipt gen(wt_`var')

        reg l_ldh i.f_score

        reg l_ldh  i.f_score [pweight =iptwt_`var'] if covid3==1
  }
  xi: pbalchk  ldh_done   i.f_score  i.triage_imp sp02  ,wt(iptwt_ldh_done  ) graph

    //detailed

        foreach var in ldh_done {
      cap drop  iptwt_`var'
       logit `var'   i.f_score i.triage_imp    sp02 c.age_y c.triage_imp

       estat gof
       cap drop propensity
        predict propensity ,pr

        propwt ldh_done propensity, ipt gen(wt_`var')

       // reg l_ldh i.f_score
       //reg l_ldh i.f_score if covid3==1

        reg l_ldh  i.f_score [pweight =iptwt_`var']    if covid3==1
  }
   xi: pbalchk  ldh_done   i.f_score  i.triage_imp sp02  ,wt(iptwt_ldh_done  ) graph



    foreach var in dimer_measured {
   cap drop iptwt_`var'
     logit `var'   i.f_score i.triage_imp pts_that_hourP21


        estat gof
       cap drop propensity
        predict propensity ,pr

        propwt dimer_measured propensity, ipt gen(wt_`var')

       logit elev_dimer f456, or

       logit elev_dimer f456 i.age_group ,or

       logit elev_dimer f456 [pw =iptwt_`var'] if covid3==1 ,or

       logit elev_dimer f456 i.age_group1 [pw =iptwt_`var']  if covid3==1 ,or
  }
  xi: pbalchk  dimer_measured  i.f_score i.triage_imp sp02  ,wt(iptwt_dimer_measured  ) graph
   // The issue here is that F56 has perfect predcition for 8 values. P values are very unstable and can get well below 0.05 using exlogisitc and manually generated weighted outcomes. But, F456 avoids perfect prediction and seems to proved estimates that appeart more reasonable.
 //detailed
     foreach var in dimer_measured {
   cap drop iptwt_`var'
     logit `var'   i.f_score i.triage_imp##c.sp02 c.age_y c.triage_imp pts_that_hourP14



        estat gof
       cap drop propensity
```

```
        predict propensity ,pr

        propwt dimer_measured propensity, ipt gen(wt_`var')

       logit elev_dimer f456 if covid3==1, or

      //logit elev_dimer f456 i.age_group1 ,or

       logit   elev_dimer f456   [pw =iptwt_`var']   ,or

      // logit elev_dimer f456 i.age_group1 [pw =iptwt_`var']  ,or
  }

  xi: pbalchk  dimer_measured   i.f_score i.triage_imp  sp02  ,wt(iptwt_dimer_measured  ) graph

 // Insert i.age_group into analysis for age group differences
```
