# Supplementary material for: Point-of-care lung ultrasound predicts hyperferritinemia and hospitalization, but not elevated troponin in SARS-CoV-2 viral pneumonitis in children
Source: Sci Rep. 2024 Mar 11;14:5899. doi: 10.1038/s41598-024-55590-9 (PMC10928070; doi:10.1038/s41598-024-55590-9)
Supplement: Supplementary file 5 — Supplementary Information 5. [file 41598_2024_55590_MOESM5_ESM.docx]

Extended Table 4

| Initial variable selection (all comers) | | | | | | | | | | | | | |
| --- | --- | --- | --- | --- | --- | --- | --- | --- | --- | --- | --- | --- | --- |
| **Method** |  | **ANOVA** | **Logit** | **Unweighted**  **OLS regression** | | **PS IPW regression** | |  | | | | | |
|  |  |  |  |  |  |  |  |  |  |  |  |  |  |
| **Variable** | **N** | **F Statistic** | **OR** | **β** | **p-value** | **β** | **p-value** |  |  |  |  |  |  |
| **All comers** |  |  |  | **Moderate to severe & severe LUS** |  |  | **Moderate to severe & severe LUS** |  |  |  |  |  |  |
| Ferritin | 49 | 3.288** | 2.89* | 481 | 0.006 | 368 | 0.033 |  |  |  |  |  |  |
| LDH | 33 | 2.837** | 4.90* | 177 | 0.023 | 155 | 0.05 |  |  |  |  |  |  |
| AST. | 53 | 3.188** | NC*** | 40 | 0.001 | 35 | 0.001 |  |  |  |  |  |  |
| ALT | 50 | 3.639** | 4.67** | 38 | 0.001 | 35 | 0.01 |  |  |  |  |  |  |
|  |  |  |  | **logit** |  | **logit** |  |  |  |  |  |  |  |
|  |  |  |  | 1.33 | 0.043 | 1.54 | 0.05 |  |  |  |  |  |  |
| D-Dimer | 43 |  | 3.78** | OR 3.78 (1.05, 13.68) |  | 4.83 (1.00,23.3) |  |  |  |  |  |  |  |
|  | **Lung ultrasound treated as continuous variable**  **Analysis by category of ultrasound severity** | | | | | | | **Analysis by category of ultrasound severity**  PS derived from all comers IPW | | | | | |
| **Variable** | **N** | **OLS regression** | | **PS IPW regression** | |  | | **Normal** | **Very mild** | **Mild** | **Mid to moderate** | **Moderate** | **Mod rate to severe & severe** |
|  |  | **β** (95%CI) | **p-value** | **β** (95%CI) | p**-value** |  |  |  |  |  |  |  |  |
| Ferritin | 49 | 83  (28, 138) | 0.004 | 42  (1, 83) | 0.046 |  |  | Referent | -75  (-194, 43) | -45  (-175, 86) | -71  (-194, 52) | 20  (-185, 224) | 367**  (33, 701) |
| LDH | 33 | 31  (5, 57) | 0.019 | 5  (-13, 24) | 0.564 |  |  | Referent | -31  (-101, 37) | -36  (-118, 48) | -50***  (-82, -18) | 5  (-63, 72) | 155**  (0, 310) |
| AST. | 53 | 6  (2,10) | 0.004 | 3 (0, 6) | 0.053 |  |  | Referent | 6  (-11, 23) | -1  (-21, 19) | 2  (-14, 17) | 8  (-11, 28) | 35***  (16, 54) |
| ALT | 50 | 6  (2, 10) | 0.006 | 3  (-1, 8) | 0.168 |  |  | Referent | 3  (-13, 20) | -1  (-12, 10) | -5  (-8, 18) | 5  (-8, 18) | 43***  (19, 67) |
|  | **SARS-CoV-2 positive only** | | | | | | | | | | | | |
|  | **Lung ultrasound treated as continuous variable** | | | | | **Analysis by category of ultrasound severity**  uses PS from SARS-CoV-2 positive patients only for balancing | | | | | | | |
| **Variable** | **N** | **OLS regression** | | **PS IPW regression** | |  | | **Normal** | **Very mild** | **Mild** | **Mid to moderate** | **Moderate** | **Moderate to severe & severe** |
|  |  | **β** | **p-value** | **β** | **p-value** |  |  |  |  |  |  |  |  |
| Ferritin | 21 | 124 | 0.01 | 87 | 0.02 |  |  | Referent | -3 (-96, 90) | Empty   cell | 8 (-61, 77) | 304 * (-45, 653) | 560 ** (119, 1001) |
| LDH | 11 | 48 | <0.01 | 17 | 0.39 |  |  | Referent | -79 (-140, -19) | -45 (-226,136) | Empty cell | 120 * (-5, 245) | 179 ** (45, 313) |
| AST. | 20 | 11 | 0.03 | 5 | 0.06 |  |  | Referent | -6 (-43, 30) | -8 (-26, 10) | 14 (-8, 36) | 17 (-9, 43) | 46** (7, 84) |
| ALT | 19 | 11 | 0.05 | 5 | 0.10 |  |  | Referent | -7 (-36, 21) | -9 ** (17, -1) | 19 (-18, 56) | 1 (-19, 21) | 42* (-6, 90) |

Laboratory tests had to pass a two-step process on all comers to be included in the final analysis. The first column shows the number of observations in each analysis. The second column shows the results of ANOVA relating ultrasound severity to mean laboratory values for each test. Where the p-value was <0.10 logistic regression relating lung ultrasound to the laboratory value being elevated was performed. These results are shown in the second column. If this result also had a p-value of <0.10 then that particular laboratory test was included in the final analysis. The fourth column shows ordinary least squares regression with laboratory value as the dependent variable and ultrasound severity as the independent variable. Ultrasound severity was treated as a categorical variable with normal as the referent. Coefficients are shown only for the ’More than moderately severe’ ultrasound group. (The coefficients for lesser degrees of ultrasound severity were generally non-significant.) The fifth column shows the final result; the propensity score derived inverse probability-weighted ordinary least squares regression with laboratory value as the dependent variable and ultrasound severity as the independent variable. There were too few cases to allow analysis using categorical outcomes in the SARS-CoV-2-only group. There was no association between any blood test and lung ultrasound severity in the SARS-CoV-2 negative group (data not shown). Screening analysis *p<0.1, **p<0.05, ***p<0.001. Where ALT was reported only as <6 (n=3) it was not included in the analysis. Values for albumin ordered as a single add-on test by the admitting service were included in the analysis. D-dimer and troponin were analyzed only as elevated or normal. Includes additional models alluded to but not shown in Table 4 in the manuscript. OLS ordinary least squares, LDH lactate dehydrogenase, AST aspartate aminotransferase, ALT alanine transaminase, OR odds ratio, NC not calculable because all were abnormal in more severe ultrasound findings, LUS lung ultrasound, logit logistic regression, PS propensity score, IPW inverse probability-weighted.
