# Supplementary material for: Point-of-care lung ultrasound predicts hyperferritinemia and hospitalization, but not elevated troponin in SARS-CoV-2 viral pneumonitis in children
Source: Sci Rep. 2024 Mar 11;14:5899. doi: 10.1038/s41598-024-55590-9 (PMC10928070; doi:10.1038/s41598-024-55590-9)
Supplement: Supplementary file 6 — Supplementary Information 6. [file 41598_2024_55590_MOESM6_ESM.rtf]

;;;013f﻿

tart report 
"ultrasound  indication: rule out infiltrate/fluid  machine:  mindray   archived:  local   probe: 10.5 mhz  image quality:good  narrative: posterior , axillary and anterior acoustic windows interrogated bilaterally. normal a lines throughout. no excess b-lines, air bronchograms or effusion. no consolidation  impression:normal lung us                "
"Normal"


Start report 
"point of care lung ultrasound  indication: rule out infiltrate/fluid  machine:  mindray   archived:  local   probe: 10.5 mhz, 4.1 mhz  image quality: excellent  narrative: posterior , axillary and anterior acoustic windows interrogated bilaterally.   impression:patch of moth-eaten pleura and long b lines in the left infrascapular window. no effusion. no consolidation.             "
"Very mild"


Start report 
"ultrasound performed at bedside  point of care limited  lung ultrasound  indication: rule out infiltrate/fluid  machine:  mindray   archived:  local   probe: 10.5 mhz  image quality: good   narrative: posterior , axillary and anterior acoustic windows interrogated bilaterally. excessive long b-lines with irregular air bronchiograms on the right. subpleural pneumonia, less than 1 cm on the right.   impression:abnormal      
"Mild to Moderate"


Start report 
"ultrasound  indication: rule out infiltrate/fluid  machine:  mindray   archived:  local   probe: 10.5 mhz  image quality:  good  narrative: posterior , axillary and anterior acoustic windows interrogated bilaterally. normal a lines throughout. no excess b-lines, air bronchograms or effusion. no consolidation  impression:normal lung us   
"Normal"


Start report 
"ultrasound  bedside lung ultrasound shows small amount of patchy viral pneumonitis in the right axillary acoustic window and some mild pleural irregularities and the intrascapular windows.  images archived locally  machine: mindray  probe: 9– megahertz  impression: very mild pneumonitis      differential diagnoses are considered as follows, but not limited to: covid pneumonitis, other viral pneumonitis, pneumonia, adenovirus. 

"Very mild"


Start report 
       point of care lung ultrasound  indication: rule out infiltrate/fluid  machine:  mindray   archived:  local   probe: 10.5 mhz  image quality: very good   narrative: posterior , axillar and anterior acoustic windows interrogated bilaterally. normal a lines, no excess b-lines, no effusion, no consolidation  impression: normal   

"Normal"


Start report 
"ultrasound.     point of care limited lung ultrasound  indication: rule out infiltrate/fluid  machine:  mindray   archived:  local   probe: 4.1mhz  image quality: good   narrative: posterior , axillar and anterior acoustic windows interrogated bilaterally.   impression: normal      
"Normal"


Start report 
"point of care lung ultrasound  indication: rule out infiltrate/fluid  machine:  mindray   archived:  local   probe: 10.5 mhz  image quality: very good  narrative: posterior , axillary and anterior acoustic windows interrogated bilaterally. air bronchograms. no excess b lines. normal short b lines. no consolidation. no effusion.  impression:bronchitis      "
"Very mild"


Start report 
"ultrasound  indication: rule out infiltrate/fluid  machine:  mindray   archived:  local   probe: 10.5 mhz  image quality: good  narrative: posterior , axillary and anterior acoustic windows interrogated bilaterally. normal a lines throughout. no excess b-lines, air bronchograms or effusion. no consolidation  impression:normal lung us            "
"Normal"


Start report 
"ultrasound radiology report  sutter medical center emergency department - sacramento    exam: poc lung ultrasound    indications: known covid exposure with symptoms    views: 2-3 views (as below)    probe: linear probe    image quality: good    procedure: using the linear array transducer i evaluated the bilateral anterolateral thorax in a systematic fashion revealing the presence of lung sliding and comet-tail artifact at the interrogated interspaces. m-mode shows normal anatomy and function.     impression: negative limited lung ultrasound with no pneumothorax identified.  these images were archived and i independently interpreted the images at the bedside.    
"Normal"

Start report 
"ultrasound  indication: rule out infiltrate/fluid  machine:  mindray   archived:  local   probe: 10.5 mhz  image quality: good  narrative: posterior , axillary and anterior acoustic windows interrogated bilaterally.   impression: normal lung exam   
"Normal"


Start report 
"  point of care limited  lung ultrasound  indication: rule out infiltrate/fluid  machine:  mindray   archived:  local   probe: 10.5 mhz  image quality: good   narrative: posterior , axillary and anterior acoustic windows interrogated bilaterally. excess long b lines right intrascapular area. some moth eaten pleura bilat  axillae clear. no effusion or consolidation   impression:pneumonitis c/w covid    
"Mild"


Start report 
"  procedures (if indicated)    .ed ultrasound     indications: rule out pneumonia      imaged archived locally in ultrasound machine. a 10 mghz ultrasound used to interrogate lung fields and heart fields. on us, patient has excess b lines with areas of consolidation, as well as pleural thickening of the right lung. there are shorter b lines notable in the left upper lung. there were no signs of cardiac effusions. there is normal contractivity.                     "
"Moderate"


Start report 
"ultrasound: lung       indications: rule out pneumonia      imaged archived locally in mindray ltrasound machine.  a 10 mghz ultrasound used to interrogate all posterior  lung fields and long and short parasternal axis cardiac fields.       on us, patient has excess long  b lines bilaterally with areas of consolidation, as well as pleural thickening of the right lung. there are excess short  b lines without excess long notable in the both upper lungs.     there was no pericardial effusions. there is normal contractility of heart.       impression: bilateral alveloar fluid and patchy consolidation on the right with small amount of pleural effuison               "
"Moderate"

Start report 
"limited bedside poc ultrasound radiology report  sutter medical center emergency department - sacramento    exam: poc lung ultrasound    indications: chest pain or shortness of breath    views: 2-3 views (as below)    probe: linear probe    image quality: good    procedure: using the linear array transducer i evaluated the bilateral posterolateral thorax in a systematic fashion revealing the presence of lung sliding with pleural thickening irregularities and b lines in some of the lung fields.      impression: findings consistent with covid.  these images were archived and i independently interpreted the images at the bedside.     
"Mild"


Start report 
"ultrasound  indication: rule out infiltrate/fluid  machine:  mindray   archived:  local   probe: 4-1mhz  image quality: good  narrative: posterior , axillary and anterior acoustic windows interrogated bilaterally. normal a lines throughout. no excess b-lines, air bronchograms or effusion. no consolidation. one area of moth eaten pleura.  impression:normal lung us     this document was transcribed by nicolo "
"Very mild"


Start report 
" bedside ultrasound showed no evidence of pleural thickening or irregularities. she had no evidence of consolidations or b lines on ultrasound."
"Normal"


Start report 
" point of care lung ultrasound  indication: rule out infiltrate/fluid  machine:  mindray   archived:  local   probe: 10-.5 mhz  image quality: good  narrative: posterior , axillar and anterior acoustic windows interrogated bilaterally. upper limitof normal short b lines. normal a lines. no effusion"
"Normal"


Start report 
"  point of care limited  lung ultrasound  indication: rule out infiltrate/fluid  machine:  mindray   archived:  local   probe: 10.5 mhz  image quality: good   narrative: posterior , axillary and anterior acoustic windows interrogated bilaterally.   impression:normal cardiac windows with no effusion.       
"Normal"


Start report 
"   point of care limited lung ultrasound     indication: rule out infiltrate/fluid  machine:  mindray   archived:  local   probe: 9-3mhz  image quality: good   narrative: posterior , axillary and anterior acoustic windows interrogated bilaterally. normal a lines throughout. no excess b-lines, air bronchograms or effusion.  "
"Normal"


Start report 
"           point of care lung ultrasound  indication: rule out infiltrate/fluid  machine:  mindray   archived:  local   probe: 10-5 mhz  image quality: good  narrative: posterior , axillary and anterior acoustic windows interrogated bilaterally. she has normal a lines, no excess b-lines, and no consolidation or effusion.  impression: normal      
"Normal"


Start report 
"point of care lung ultrasound  indication: rule out infiltrate/fluid  machine:  mindray   archived:  local   probe: 4.1mhz  image quality: fair   narrative: posterior , axillar and anterior acoustic windows interrogated bilaterally. moth-eaten pleura in and excess long coalescent b lines  in right > left intrascaupluar windows and  axillary windows. no effusion or focal consolidation     impression: pneumonitis c/w covid          
"Mild to Moderate"


Start report 
"point of care lung ultrasound  indication: rule out infiltrate/fluid  machine:  mindray   archived:  local   probe: 10.5 mhz  image quality: ***  narrative: posterior acoustic windows interrogated bilaterally.   impression:borderline excess B lines in 1 field.   "
"Very mild"


Start report 
"point of care lung ultrasound  indication: rule out infiltrate/fluid  machine:  mindray   archived:  local   probe: 10.5 mhz  image quality: normal  narrative: posterior , axillary and anterior acoustic windows interrogated bilaterally. normal  impression: normal    "
"Normal"


Start report 
"    point of care lung ultrasound  indication: rule out infiltrate/fluid  machine:  mindray   archived:  local   probe: 10.5 mhz  image quality: very good   narrative: posterior , axillar and anterior acoustic windows interrogated bilaterally. normal a lines,. upper liit normal short b lines. no long b lines no effusion or consolidation   impression:normal     "
"Normal"

Start report 
"ultrasound  indication: rule out infiltrate/fluid  machine:  mindray   archived:  local   probe: 10.5 mhz  image quality: good  narrative: posterior , axillary and anterior acoustic windows interrogated bilaterally. normal a lines throughout. no excess b-lines, air bronchograms or effusion. no consolidation  impression:normal lung us    
"Normal"


Start report 
"ultrasound was done showing a subcentimeter consol"
"Very mild"


Start report 
" limited bedside poc ultrasound radiology report  sutter medical center emergency department - sacramento    exam: poc limited lung ultrasound    indications: chest pain    views: 2-3 views (as below)    probe: linear array    image quality: excellent    procedure:  using the linear array transducer i evaluated the bilateral anterior, posterior, superior and inferior thorax in a systematic fashion "
"Normal"


Start report 
"ultrasound. consistent with very mild covid. see report below.       point of care limited lung ultrasound   indication: rule out infiltrate/fluid  machine:  mindray   archived:  local   probe: 9-3 mhz  image quality: good   narrative: posterior , axillary and anterior acoustic windows interrogated bilaterally. pleural thickening intrascapular r>l. no excessive b lines. consistent with very mild covid.   impression: normal lung us      
"Very mild"


Start report 
")     us:  point of care lung ultrasound  indication: rule out infiltrate/fluid  machine:  mindray   archived:  local   probe: 10.5 mhz  image quality: ***  narrative: posterior , axillar and anterior acoustic windows interrogated bilaterally. excess b lines. excess air bronchograms. crowded, consistent with right base. lower acoustic window and right axilla. upper limit of normal. short b  lines.   impression: lobar pneumonia on right.       medical decision making     "
"Moderate"


Start report 
"ultrasound  indication: rule out infiltrate/fluid  machine:  mindray   archived:  local   probe: 10.5 mhz  image quality: very good   narrative: posterior , axillary and anterior acoustic windows interrogated bilaterally. normal a lines throughout. no excess b-lines, air bronchograms or effusion. no consolidation . one inch spot with increased long b line in the right intrascapular regioun  impression:normal lung us   
"Very mild"


Start report 
" ultrasound probe 1    impressions: normal. no effusions. no b lines. normal a lines.  **PM patient seen and examined. ultrasound is reassuring. will order covid swab. advised mother of return precautions."
"Normal"


Start report 
"ultrasound  indication: rule out infiltrate/fluid  machine:  mindray   archived:  local   probe: 10.5 mhz  image quality: good  narrative: posterior , axillary and anterior acoustic windows interrogated bilaterally. normal a lines throughout. no excess b-lines, air bronchograms or effusion. no consolidation  impression:normal lung us   
"Normal"


Start report 
"ultrasound  indication: rule out infiltrate/fluid  machine:  mindray   archived:  local   probe: 10.5 mhz  image quality: good  narrative: posterior , axillary and anterior acoustic windows interrogated bilaterally. normal a lines throughout. no excess b-lines, air bronchograms or effusion. no consolidation  impression:normal lung us "
"Normal"


Start report 
"ultrasound  indication: rule out infiltrate/fluid  machine:  mindray   archived:  local   probe: 10.5 mhz  image quality: normal  narrative: posterior , axillary and anterior acoustic windows interrogated bilaterally. normal a lines throughout. no excess b-lines, air bronchograms or effusion. no consolidation. small area with slightly rough pleura  impression:normal lung us      
"Very mild"


Start report 
"ultrasound  indication: rule out infiltrate/fluid  machine:  mindray   archived:  local   probe: 10.5 mhz  image quality: good  narrative: posterior , axillary and anterior acoustic windows interrogated bilaterally. normal a lines throughout. no excess b-lines, air bronchograms or effusion. no consolidation  impression:normal lung us    
"Normal"


Start report 
"ultrasound radiology report  sutter medical center emergency department - sacramento    exam: poc lung ultrasound    indications: known covid exposure with symptoms    views: 2-3 views (as below)    probe: linear probe    image quality: good    procedure: using the linear array transducer i evaluated the bilateral anterolateral thorax in a systematic fashion revealing the presence of lung sliding and comet-tail artifact at the interrogated interspaces. m-mode shows normal anatomy and function.     impression: negative limited lung ultrasound with no pneumothorax identified.  these images were archived and i independently interpreted the images at the bedside.  
"Normal"


Start report 
"  point of care lung ultrasound  indication: rule out infiltrate/fluid  machine:  mindray   archived:  local   probe: 10.5 mhz  image quality: good  narrative: posterior , axillar and anterior acoustic windows interrogated bilaterally.significant excess short and long b lines intrascapular region. some pleura thickening. no effusion or consolidation "
"Mild to Moderate"


Start report 
"point of care lung ultrasound  indication: rule out infiltrate/fluid  machine:  mindray   archived:  local   probe: 10.5 mhz  image quality:good   narrative: posterior , axillar and anterior acoustic windows interrogated bilaterally. excess intrascapular  b lines. no effusion or consolidation.   impression:pneumonitis c/w covid      "
"Mild"


Start report 
"  point of care limited  lung ultrasound  indication: rule out infiltrate/fluid  machine:  mindray   archived:  local   probe: 10.5 mhz  image quality: good   narrative: posterior , axillary and anterior acoustic windows interrogated bilaterally. few air bronchograms. no excess b lines. no effusion. no consolidation.  impression c/w mild bronchitis. no evidence for covid-19.    "
"Very mild"


Start report 
"ultrasound   indication: rule out infiltrate/fluid  machine:  mindray   archived:  local   probe: 9-3 mhz  image quality: good   narrative: posterior , axillary and anterior acoustic windows interrogated bilaterally.  he has some moth-eaten pleura and occasional excess long b-lines in the left and right axilla.  the posterior and anterior windows are normal.  no effusion.  no consolidation.  impression: abnormal lung us consistent with very mild pneumonitis.     very mild viral pneumonitis, left and right axillae.      "
"Very mild"


Start report 
" bedside ultrasound performed showing no signs of covid-19.               "
"Normal"


Start report 
"  point of care lung ultrasound  indication: rule out infiltrate/fluid  machine:  mindray   archived:  local   probe: 10.5 mhz  image quality: good   narrative: posterior acoustic windows interrogated bilaterally. normal a lines. occasional b lines in normal range. no pleural effusion.  impression: ***      "
"Normal"


Start report 
"ultrasound     indication: rule out infiltrate/fluid  machine:  mindray   archived:  local   probe: 9-3mhz  image quality: good   narrative: posterior , axillary and anterior acoustic windows interrogated bilaterally. normal a lines throughout. no excess b-lines, air bronchograms or effusion. no consolidation  impression:normal lung us        
"Normal"

Start report 
"  point of care lung ultrasound  indication: rule out infiltrate/fluid  machine:  mindray   archived:  local   probe: 4.1mhz  image quality: good  narrative: posterior , axillar and anterior acoustic windows interrogated bilaterally. ***  impression:***     "
"Normal"


Start report 
"ultrasound  indication: rule out infiltrate/fluid  machine:  mindray   archived:  local   probe: 10.5 mhz  image quality: good  narrative: posterior , axillary and anterior acoustic windows interrogated bilaterally. normal a lines throughout. no excess b-lines, air bronchograms or effusion. no consolidation  impression:normal lung us          
"Normal"

Start report 
" indication: rule out infiltrate/fluid  machine:  mindray   archived:  local   probe: 9-3 mhz  image quality: very goood   narrative: posterior , axillary and anterior acoustic windows interrogated bilaterally.  there are extensive b-lines and associated moth eaten pleura intrascapularly left greater than right and in the axilla to a lesser extent.  impression: abnormal lung us consistent with covid.        "
"Moderate to severe"


Start report 
"ultrasound  indication: rule out infiltrate/fluid  machine:  mindray   archived:  local   probe: 10.5 mhz  image quality: goodnarrative: posterior , axillary and anterior acoustic windows interrogated bilaterally. normal a lines throughout. no excess b-lines, air bronchograms or effusion. no consolidation  impression:normal lung us     
"Normal"


Start report 
"ultrasound   indication: rule out infiltrate/fluid  machine:  mindray   archived:  local   probe: 9-3 mhz  image quality: good   narrative: posterior , axillary and anterior acoustic windows interrogated bilaterally. moth-eaten pleura bilaterally. no effusion or consolidation. compression consistent with very mild viral pneumonitis.  impression: abnormal lung us     "
"Mild"


Start report 
"  point of care lung ultrasound  indication: rule out infiltrate/fluid  machine:  mindray   archived:  local   probe: 10.5 mhz  image quality: good  narrative: posterior and axillar acoustic windows interrogated bilaterally.   impression: diffuse a-lines, no evidence of b-lines, no effusion  "
"Normal"


Start report 
" point of care lung ultrasound  indication: rule out infiltrate/fluid  machine:  mindray   archived:  local   probe: 10.5 mhz  image quality: good  narrative: posterior and axillar acoustic windows interrogated bilaterally.   impression: diffuse a-lines, no evidence of b-lines, no effusion"
"Normal"


Start report 
"ultrasound  indication: rule out infiltrate/fluid  machine:  mindray   archived:  local   probe: 10.5 mhz  image quality: good  narrative: posterior , axillary and anterior acoustic windows interrogated bilaterally. normal a lines throughout. no excess b-lines, air bronchograms or effusion. no consolidation  impression:normal lung us"   
"Normal"


Start report 
"   point of care lung ultrasound  indication: rule out infiltrate/fluid  machine:  mindray   archived:  local   probe: 10.5 mhz  image quality: goodnarrative: posterior , axillary and anterior acoustic windows interrogated bilaterally. 1 right sided subpleural subcentimeter focal pneumonia. no excess b-lines elsewhere. no effusion. no significant consolidation.  impression: as this finding was isolated no treatment indicated.   "
"Very mild"
"

Start report 
"ultrasound     indication: rule out infiltrate/fluid  machine:  mindray   archived:  local   probe: 9-3mhz  image quality: good   narrative: posterior , axillary and anterior acoustic windows interrogated bilaterally. normal a lines throughout. no excess b-lines, air bronchograms or effusion. no consolidation  impression:normal lung us         reviewed and electronically signed by paul walsh md.     
"Normal"


Start report 
"  point of care limited  lung ultrasound  indication: rule out infiltrate/fluid  machine:  mindray   archived:  local   probe: 10.5 mhz  image quality: good   narrative: posterior , axillary and anterior acoustic windows interrogated bilaterally. diffuse excess long coalescent b lines. bronchograms in the left. no pleural effusion or consolidation.   impression: findings consistent with moderate severity pneumonitis, bronchiolitis or chf.      
"Moderate"


Start report 
"ultrasound  indication: rule out infiltrate/fluid  machine:  mindray   archived:  local   probe: 10.5 mhz  image quality: good  narrative: posterior , axillary and anterior acoustic windows interrogated bilaterally. normal a lines throughout. no excess b-lines, air bronchograms or effusion. no consolidation  impression:normal lung us   
"Normal"


Start report 
" the ultrasound doesn't show any evidence of lung infection." 
"Normal"


Start report 
"  point of care lung ultrasound  indication: rule out infiltrate/fluid  machine:  mindray   archived:  local   probe: 10.5 mhz  image quality: very good.  narrative: posterior , axillar and anterior acoustic windows interrogated bilaterally. normal a-lines. no b-lines.  impression: no effusion. no consolidation.  "
"Normal"


Start report 
"  point of care lung ultrasound  indication: rule out infiltrate/fluid  machine:  mindray   archived:  local   probe: 10.5 mhz  image quality: very good.  narrative: posterior , axillary and anterior acoustic windows interrogated bilaterally. normal a-lines. no b-lines. no effusion. no consolidation.    impression:normal    this document was transcribed by julianna c rojo, cms.    signature & attestation: all medical record entries made
"Normal"


Start report 
"ultrasound  indication: rule out infiltrate/fluid  machine:  mindray   archived:  local   probe: 10.5 mhz  image quality: good   narrative: posterior , axillary and anterior acoustic windows interrogated bilaterally. excess b lines in the right posterior lung field excess b lines in the right posterior lung. some moth-eaten pleura i nthe left axilla.   impression: mild patchy pneumonitis c/w covid     "
"Mild"


Start report 
"point of care lung ultrasound  indication: rule out infiltrate/fluid  machine:  mindray   archived:  local   probe: 10.5 mhz  image quality: normal  narrative: posterior , axillary and anterior acoustic windows interrogated bilaterally. normal  impression:normal     "
"Normal"


Start report 
"point of care lung ultrasound  indication: rule out infiltrate/fluid  machine:  mindray   archived:  local   probe: 10.5 mhz  image quality: normal  narrative: posterior , axillary and anterior acoustic windows interrogated bilaterally. normal  impression:normal      "
"Normal"


Start report 
"ultrasound   indication: rule out infiltrate/fluid  machine:  mindray   archived:  local   probe: 9-3 mhz  image quality: good   narrative: posterior , axillary and anterior acoustic windows interrogated bilaterally. moth-eaten pleura in the intrascapular windows, occasional b-lines, no effusion or consolidation   impression: abnormal lung us, consistent with mild viral pneumonitis  "
"Mild"


Start report 
"  point of care lung ultrasound  indication: rule out infiltrate/fluid  machine:  mindray   archived:  local   probe: 10.5 mhz and 4.1 mhz  image quality: fair   narrative: posterior acoustic windows interrogated bilaterally. excess b lines intrascapular bilat r >left with  localized consolidation on the right. no effusion + air bronchograms right. no moth eaten pleura.  impression: consolidation in the right intrascapular area with excess b-lines consistent with covid or  other patchy pneum"
"Mild to Moderate"


Start report 
"ultrasound   indication: rule out infiltrate/fluid  machine:  mindray   archived:  local   probe: 9-3 mhz  image quality: good   narrative: posterior , axillary and anterior acoustic windows interrogated bilaterally.  there were multiple areas of long excess b-lines mostly in the right interscapular area with less severe findings in the left interscapular area and relative sparing of the axilla.  no effusion no focal consolidations  impression: abnormal lung us consistent with viral pneumonitis.      
"Moderate to severe"


Start report 
"ultrasound  indication: rule out infiltrate/fluid  machine:  mindray   archived:  local   probe: 10.5 mhz  image quality: goodd  narrative: posterior , axillary and anterior acoustic windows interrogated bilaterally. normal a lines throughout. no excess b-lines, air bronchograms or effusion. no consolidation. one window less than upper limit of b lines only. cardiac window showed normal contractility   impression:normal lung us   
"Normal"


Start report 
"   point of care limited lung ultrasound   indication: rule out infiltrate/fluid  machine:  mindray   archived:  local   probe: 9-3 mhz  image quality: good   narrative: posterior , axillary and anterior acoustic windows interrogated bilaterally.  he has long b-lines with moth-eaten pleura right posterior acoustic windows, left posterior acoustic windows and right axilla.  he has several pustule is less than 0.5 cm each with some surrounding irregular air bronchograms "
"Moderate"


Start report 
"    point of care lung ultrasound  indication: rule out infiltrate/fluid  machine:  mindray   archived:  local   probe: 10.5 mhz  image quality: good  narrative: posterior , axillary and anterior acoustic windows interrogated bilaterally.   impression:  excess short B lines diffusely on the posterior windows. moth eaten pleural diffusely bilaterally on the left lower bases. excess long B lines in the left axilla. findings are consistent with viral pneumonitis or pneumonia.   "
"Moderate"


Start report 
"point of care lung ultrasound  indication: rule out infiltrate/fluid  machine:  mindray   archived:  local   probe: 10.5 mhz  image quality: 1  narrative: posterior , axillary and anterior acoustic windows interrogated bilaterally. the left has some increased b lines on the right base. excess long b lines.  impression: patchy viral pneumonitis"
"Mild"


Start report 
"ultrasound     indication: rule out infiltrate/fluid  machine:  mindray   archived:  local   probe: 9-3mhz  image quality: good   narrative: posterior , axillary and anterior acoustic windows interrogated bilaterally. normal a lines throughout. no excess b-lines, air bronchograms or effusion. no consolidation  impression:normal lung us    .    "Normal"


Start report 
"ultrasound  indication: rule out infiltrate/fluid  machine:  mindray   archived:  local   probe: 10.5 mhz  image quality: good  narrative: posterior , axillary and anterior acoustic windows interrogated bilaterally. normal a lines throughout. minimal b-lines left lung with no air bronchograms or effusion. no consolidation  impression:normal lung us   "
"Very mild"


Start report 
"  point of care limited  lung ultrasound  indication: rule out infiltrate/fluid  machine:  mindray   archived:  local   probe: 10.3 mhz  image quality: good   narrative: posterior , axillary and anterior acoustic windows interrogated bilaterally. ***  findings: moth eaten plethora in bilateral intrascapular and acoustic window.   impression: mild penumonitis         "
"Mild"


Start report 
"  point of care limited  lung ultrasound  indication: rule out infiltrate/fluid  machine:  mindray   archived:  local   probe: 10.3 mhz  image quality: good   narrative: posterior , axillary and anterior acoustic windows interrogated bilaterally.    findings: moth eaten plethora in bilateral intrascapular and acoustic window. no effusion or consolidation   impression: mild penumonitis      "
"Mild"


Start report 
"  point of care limited  lung ultrasound  indication: rule out infiltrate/fluid  machine:  mindray   archived:  local   probe: 10.5 mhz  image quality: good   narrative: posterior , axillary and anterior acoustic windows interrogated bilaterally. left upper lobe with slight increase in b lines that are mostly short. on the right axilla , excess long b lines.   impression very mild patchy pneumonitis  "
"Very mild"


Start report 
" point of care lung ultrasound  indication: rule out infiltrate/fluid  machine:  mindray   archived:  local   probe: 10.5 mhz  image quality: good  narrative: posterior , axillary and anterior acoustic windows interrogated bilaterally with evidence of scattered b lines.   impression: scattered b lines    "
"Very mild"


Start report 
"  point of care limited  lung ultrasound  indication: rule out infiltrate/fluid  machine:  mindray   archived:  local   probe: 10.5 mhz  image quality: good   narrative: posterior , axillary and anterior acoustic windows interrogated bilaterally. ***  impression: left lung with multiple long b lines, mostly linear  bronchoairgrams. left lung base there are areas of multifocal irregularly placed air bronchograms c/w pneumonia.   tiny pleural effusion on the right base with irregular areas of consolidation about 1 cm depth.     "
"Moderate"


Start report 
"point of care limited  lung ultrasound  indication: rule out infiltrate/fluid  machine:  mindray   archived:  local   probe: 10.5 mhz  image quality: good   narrative: posterior , axillary and anterior acoustic windows interrogated bilaterally.  right  lung:  with multiple long b lines , beneath this   linear  bronchoairgrams. beneath this near the right lung base there are areas of multifocal irregularly placed air bronchograms c/w pneumonia.  left lung tiny pleural effusion on the left base with irregular areas of consolidation about 1 cm depth.   impression :extensive right sided consolidation c/w pna and smuch smallef left sided pna   "
"Severe"


Start report 
"ultrasound   indication: rule out infiltrate/fluid  machine:  mindray   archived:  local   probe: 9-3 mhz  image quality: good   narrative: posterior , axillary and anterior acoustic windows interrogated bilaterally. diffuse air bronchogram's on left lung with b lines in base.   irregularly spaced air bronchograms and pleural thickening.  b lines on left base and left midzone.   depth greater than 1 cm.  long b lines in upper posterior acoustic constrict windowns.  some b lines located on right upper.  irregularly spaced air bronchogram's on right base consistent with pneumonia.  incidental finding of mild hydronephrosis on left kidney.     impression: abnormal lung us   "
"Moderate"


Start report 
 "ultrasound radiology report  sutter medical center emergency department - sacramento    exam: poc limited lung ultrasound    indications: fever or shortness of breath    views: 2-3 views (as below)    probe: linear array    image quality: good    procedure:  using the linear array transducer i evaluated the bilateral anterior, posterior and lateral thorax in a systematic fashion revealing the presence of significant consolidation lll. mild diffuse b lines. there is the presence of lung sliding and comet-tail artifact at the interrogated interspaces. m-mode shows normal anatomy and function.    impression:  point of care limited pediatric lung ultrasound with the presence of a consolidation consistent with pneumonia absence of a pneumonthorax and findings consistent with bronchiolitis. "
"Mild to Moderate"


Start report 
"ultrasound   indication: rule out infiltrate/fluid  machine:  mindray   archived:  local   probe: 9-3 mhz  image quality: good   narrative: posterior , axillary and anterior acoustic windows interrogated bilaterally. excess long b lines and moth eaten pluera at left base and right area with no focal consolictiaon  impression: consistent with covid, abnormal lung us   "
"Mild to Moderate"


Start report 
"ultrasound     indication: rule out infiltrate/fluid  machine:  mindray   archived:  local   probe: 9-3mhz  image quality: good   narrative: posterior , axillary and anterior acoustic windows interrogated bilaterally. normal a lines throughout. no excess b-lines, air bronchograms or effusion. no consolidation  impression:normal lung us            "
"Normal"

Start report 
"ultrasound.     indication: rule out infiltrate/fluid  machine:  mindray   archived:  local   probe: 9-3 mhz  image quality: very good  narrative: posterior , axillary and anterior acoustic windows interrogated bilaterally. diffuse excess long b lines, moth-eaten pleura. consistent with covid pneumonitis.   impression: abnormal lung us   
"Severe"


Start report 
"point of care lung ultrasound  indication: rule out infiltrate/fluid  machine:  mindray   archived:  local   probe: 10.5 mhz  image quality: good  narrative: posterior , axillary and anterior acoustic windows interrogated bilaterally he has 2 tiny foci of excess long b-lines one in the left axilla and one in the right intrascapular posterior window.    impression: consistent with very mild covid pneumonitis       records reviewed:  nursing notes  relevant past records   "
"Very mild"


Start report 
"ultrasound  indication: rule out infiltrate/fluid  machine:  mindray   archived:  local   probe: 10.5 mhz  image quality:good  narrative: posterior , axillary and anterior acoustic windows interrogated bilaterally. normal a lines throughout. no excess b-lines, air bronchograms or effusion. no consolidation  impression:normal lung us    
"Normal"


Start report 
"ultrasound  indication: rule out infiltrate/fluid  machine:  mindray   archived:  local   probe: 10.5 mhz  image quality: good  narrative: posterior , axillary and anterior acoustic windows interrogated bilaterally. normal a lines throughout. no excess b-lines, air bronchograms or effusion. no consolidation  impression:normal lung us   "
"Normal"


Start report 
"ultrasound  indication: rule out infiltrate/fluid  machine:  mindray   archived:  local   probe: 10.5 mhz  image quality: good  narrative: posterior , axillary and anterior acoustic windows interrogated bilaterally. normal a lines throughout. no excess b-lines, air bronchograms or effusion. no consolidation  impression:normal lung us     "
"Normal"


Start report 
"ultrasound   sutter medical center emergency department - sacramento    exam: poc lung ultrasound    indications: rule out pneumonia     probe: 10.5 mghz linear probe    image quality: good    procedure: using the linear array transducer i evaluated the bilateral anterolateral thorax in a systematic fashion revealing the presence of lung sliding and comet-tail artifact at the interrogated interspaces. m-mode shows normal anatomy and function.     findings: excess long and short B lines throughout right lung. B lines in left lung field is the upper limit of normal.     impression:findings suggest pneumonia in the right lung field. left lung fields are negative for pneumonia. these images were archived and i in"
"Mild to Moderate"


Start report 
"ultrasound     indication: rule out infiltrate/fluid  machine:  mindray   archived:  local   probe: 9-3mhz  image quality: poor because child unable to sit still.  narrative: posterior , axillary and anterior acoustic windows interrogated bilaterally. normal a lines throughout. no excess b-lines, air bronchograms or effusion. no consolidation  impression:normal lung us     "
"Normal"


Start report 
"  point of care limited  lung ultrasound  indication: rule out infiltrate/fluid  machine:  mindray   archived:  local   probe: 10.5 mhz  image quality: good   narrative: posterior , some upper limit of normal long b lines shown in right axilla. left axilla normal.   impression: consistent with early pneumonitis but very mild   "
"Very mild"


Start report 
"ultrasound  indication: rule out infiltrate/fluid  machine:  mindray   archived:  local   probe: 10.5 mhz  image quality: good  narrative: posterior , axillary and anterior acoustic windows interrogated bilaterally. normal a lines throughout. no excess b-lines, air bronchograms or effusion. no consolidation  impression:normal lung us       "
"Normal"


Start report 
"1 point of care limited  lung ultrasound  indication: rule out infiltrate/fluid  machine:  mindray   archived:  local   probe: 10.5 mhz  image quality: good   narrative: posterior , axillary and anterior acoustic windows interrogated bilaterally. some minor moth eaten pleura and long b lines intrasacpularly bilaterally. very mild no axillary involvement. no effusion/consolidation   impression:borderline for very mild pneumonitis    
"Very mild"


Start report 
"1point of care limited  lung ultrasound  indication: rule out infiltrate/fluid  machine:  mindray   archived:  local   probe: 10.5 mhz  image quality: good   narrative: posterior , axillary and anterior acoustic windows interrogated bilaterally. some minor moth eaten pleura and long b lines intrasacpularly bilaterally. very mild no axillary involvement. no effusion/consolidation   impression:borderline for very mild pneumonitis    " 
"Very mild"


Start report 
"ultrasound  indication: rule out infiltrate/fluid  machine:  mindray   archived:  local   probe: 10.5 mhz  image quality: good   narrative: posterior , axillary and anterior acoustic windows interrogated bilaterally. occasional air bronchograms.   impression:  essentially normal lung ultrasound with tiny amount of bronchitis     
"Very mild"


Start report 
"   ultrasound probe 1     impression: normal. no B lines. normal a lines. no pleural thickening. no effusions or consolidations.    ultrasound is normal.        "
"Normal"


Start report 
"ultrasound     indication: rule out infiltrate/fluid  machine:  mindray   archived:  local   probe: 9-3mhz  image quality: good   narrative: posterior , axillary and anterior acoustic windows interrogated bilaterally. normal a lines throughout. no excess b-lines, air bronchograms or effusion. no consolidation  impression:normal lung us        
"Normal"


Start report 
"ultrasound  indication: rule out infiltrate/fluid  machine:  mindray   archived:  local   probe: 10.5 mhz  image quality: good  narrative: posterior , axillary and anterior acoustic windows interrogated bilaterally. normal a lines throughout. no excess b-lines, air bronchograms or effusion. no consolidation  impression:normal lung us  
"Normal"


Start report 
"  point of care limited  lung ultrasound  indication: rule out infiltrate/fluid  machine:  mindray   archived:  local   probe: 10.5 mhz  image quality: good   narrative: posterior , axillary and anterior acoustic windows interrogated bilaterally.  mild and long b-lines with mild moth-eaten pleura.  no effusion no consolidation.  impression:  consistent with mild pneumonitis.      "
"Mild"


Start report 
"ultrasound  indication: rule out infiltrate/fluid  machine:  mindray   archived:  local   probe: 10.5 mhz  image quality: good   narrative: posterior , axillary and anterior acoustic windows interrogated bilaterally. increased linear air bronchiograms bilaterally. no effusion or consolidation.   impression:abnormal "
"Mild"


Start report 
"ultrasound   indication: rule out infiltrate/fluid  machine:  mindray   archived:  local   probe: 9-3 mhz  image quality: good   narrative: posterior , axillary and anterior acoustic windows interrogated bilaterally.  she has bilateral moth-eaten pleura but only rare b-lines.  sparing of the axilla.  no effusion, no consolidation    impression: abnormal lung us consistent with very mild pneumonitis     "
"Very mild"


Start report 
"ultrasound  indication: rule out infiltrate/fluid  machine:  mindray   archived:  local   probe: 10.5 mhz  image quality: normal  narrative: posterior , axillary and anterior acoustic windows interrogated bilaterally. normal a lines throughout. no excess b-lines, air bronchograms or effusion. no consolidation  impression:normal lung us     medical decision making      "
"Normal"


Start report 
"ultrasound  indication: rule out infiltrate/fluid  machine:  mindray   archived:  local   probe: 10.5 mhz  image quality: good  narrative: posterior , axillar and anterior acoustic windows interrogated bilaterally. normal A lines.  normal B lines. excess B lines with pleural thickening in left axillary window . no effusion.   impression: pneumonia     
"Mild to Moderate"


Start report 
"limited bedside poc ultrasound radiology report  sutter medical center emergency department - sacramento    exam: poc limited lung ultrasound    indications: fever or shortness of breath    views: 2-3 views (as below)    probe: linear array    image quality: good    procedure:  using the linear array transducer i evaluated the bilateral anterior, posterior and lateral thorax in a systematic fashion revealing the presence of scatteredt b-lines  there is the presence of lung sliding and comet-tail artifact at the interrogated interspaces.     impression:  point of care limited pediatric lung ultrasound with the absence of a consolidation consistent with pneumonia absence of a pneumonthorax and findings consistent with bronchiolitis.  these images were archived and i independently interpreted the images at the bedside.    "
"Mild"


Start report 
"ultrasound   indication: rule out infiltrate/fluid  machine:  mindray   archived:  local   probe: 4-1mhz  image quality: good   narrative: posterior , axillary and anterior acoustic windows interrogated bilaterally. normal a lines throughout. no excess b-lines, air bronchograms or effusion. no consolidation  impression:normal lung us. short b-lines in axilla.      "
"Normal"


Start report 
"    point of care lung ultrasound  indication: rule out infiltrate/fluid  machine:  mindray   archived:  local   probe: 10.5 mhz  image quality: good  narrative: posterior and axillar acoustic windows interrogated bilaterally.   impression: diffuse a-lines, no evidence of b-lines, no effusion      "
"Normal"


Start report 
"ultrasound  indication: rule out infiltrate/fluid  machine:  mindray   archived:  local   probe: 10.5 mhz  image quality: good  narrative: posterior , axillary and anterior acoustic windows interrogated bilaterally. normal a lines throughout. few normal short b lines no excess b-lines, air bronchograms or effusion. no consolidation  impression:normal lung us   
"Normal"


Start report 
"point of care lung ultrasound  indication: rule out infiltrate/fluid  machine:  mindray   archived:  local   probe: 10.5 mhz  image quality: excellent  narrative: posterior , axillar and anterior acoustic windows interrogated bilaterally.     impression: normal. normal a lines. no b lines. no consolidation.        
"Normal"


Start report 
"ultrasound  indication: rule out infiltrate/fluid  machine:  mindray   archived:  local   probe: 10.5 mhz  image quality: good  narrative: posterior , axillary and anterior acoustic windows interrogated bilaterally. normal a lines throughout. no excess b-lines, air bronchograms or effusion. no consolidation  impression:normal lung us  upper limit of normal short b-lines diffusely.  "
"Normal"


Start report 
"ultrasound  indication: rule out infiltrate/fluid  machine:  mindray   archived:  local   probe: 10.5 mhz  image quality: good  narrative: posterior , axillary and anterior acoustic windows interrogated bilaterally. normal a lines throughout. no excess b-lines, air bronchograms or effusion. no consolidation  impression:normal lung us     reviewed and electronically signed by paul walsh md.   "
"Normal"


Start report 
" ultrasound was unremarkable. ","Normal"

161
"ultrasound radiology report  sutter medical center emergency department - sacramento    exam: poc lung ultrasound    indications: chest pain or shortness of breath    views: 2-3 views (as below)    probe: linear probe    image quality: good    procedure: using the linear array transducer i evaluated the bilateral anterolateral thorax in a systematic fashion revealing the presence of lung sliding and comet-tail artifact at the interrogated interspaces. m-mode shows normal anatomy and function.     impression: negative limited lung ultrasound with no pneumothorax ident"
"Normal"


Start report 
"ultrasound  indication: rule out infiltrate/fluid  machine:  mindray   archived:  local   probe: 4.1mhz  image quality:   narrative: posterior , axillary acoustic windows interrogated bilaterally.  he has excess b-lines with pleural thickening in the intrascapular acoustic windows bilaterally and axilla bilaterally.  impression viral pneumonitis versus bronchiolitis   "
"Moderate"


Start report 
"ultrasound  indication: rule out infiltrate/fluid  machine:  mindray   archived:  local   probe: 10.5 mhz  image quality:good   narrative: posterior , axillary and anterior acoustic windows interrogated bilaterally. normal a lines throughout. no excess b-lines, air bronchograms or effusion. no consolidation  impression:normal lung us     "
"Very mild"


Start report 
"ultrasound  indication: rule out infiltrate/fluid  machine:  mindray   archived:  local   probe: 10.5 mhz  image quality: good  narrative: posterior , axillary and anterior acoustic windows interrogated bilaterally. normal a lines throughout. no excess b-lines, air bronchograms or effusion. no consolidation  impression:normal lung us   "
"Normal"


Start report 
"     point of care lung ultrasound  indication: rule out infiltrate/fluid  machine:  mindray   archived:  local   probe: 10.5 mhz  image quality: good  narrative: posterior , axillar and anterior acoustic windows interrogated bilaterally. no consolidation. normal a-line normal b lines   impression:normal    "
"Normal"


Start report 
"ultrasound     indication: rule out infiltrate/fluid  machine:  mindray   archived:  local   probe: 9-3mhz  image quality: good   narrative: posterior , axillary and anterior acoustic windows interrogated bilaterally. normal a lines throughout. no excess b-lines, air bronchograms or effusion. no consolidation  impression:normal lung us         reviewed and electronically signed by paul walsh md.   
"Normal"


Start report 
" point of care lung ultrasound  indication: rule out infiltrate/fluid  machine:  mindray   archived:  local   probe: 10.5 mhz  image quality: good   narrative: posterior , axillary and anterior acoustic windows interrogated bilaterally.  he has excess long and short b-lines bilaterally in the intrascapular windows and in the right axilla. no effusion. some air bronchograms. no consolidation.  impression: ultrasound consistent with pneumonitis/bronchiolitis.     "
"Mild to Moderate"


Start report 
"    point of care lung ultrasound  indication: rule out infiltrate/fluid  machine:  mindray   archived:  local   probe: 10.5 mhz  image quality: very good   narrative: posterior , axillar and anterior acoustic windows interrogated bilaterally. excess short b lines diffusely. thickened pleura. long blines upper limit normal - occassionally beyond that in some windows. no effusion   impression: pneumonitis - c/w covid"    
"Mild"


Start report 
"ultrasound   indication: rule out infiltrate/fluid  machine:  mindray   archived:  local   probe: 9-3 mhz  image quality: good   narrative: posterior , axillary and anterior acoustic windows interrogated bilaterally.  he has excess long b-lines an moth-eaten pleura and the lower right base only.  there is sparing of the eczema.  no consolidation.  no effusion  impression: abnormal lung us  consistent with very mild viral pneumonitis.    
"Mild"


Start report 
"ultrasound  sutter medical center emergency department - sacramento    exam: lung ultrasound    indications: rule out pneumonia     probe: 10.5 mghz linear probe     image quality: good    procedure: using the linear array transducer i evaluated the bilateral anterolateral thorax in a systematic fashion revealing the presence of lung sliding and comet-tail artifact at the interrogated interspaces. m-mode shows normal anatomy and function.    findings: upper limit of normal b lines of bases of both lung fields. there is atelectasis noted on the left lung and a non-linear broncohogram definitive for infiltrate. negative for pleural effusions     impression: no notable findings definitive for pneumonia. these images were archived and i independently interpreted the images at the bedside."
"Normal"


Start report 
"ultrasound  indication: rule out infiltrate/fluid  machine:  mindray   archived:  local   probe: 10.5 mhz  image quality: limited by child not staying still  narrative: posterior , axillary and anterior acoustic windows interrogated bilaterally. normal a lines throughout. no excess b-lines, air bronchograms or effusion. no consolidation  impression:normal lung us, upper limit of normal b lines     "
"Normal"


Start report 
"point of care lung ultrasound  indication: rule out infiltrate/fluid  machine:  mindray   archived:  local   probe: 10.5 mhz  image quality: good  narrative: posterior, axillar and anterior acoustic windows interrogated bilaterally. normal a-lines, no excess b-lines, no consolidation, no effusion  impression: normal us  "
"Normal"


Start report 
"ultrasound  indication: rule out infiltrate/fluid  machine:  mindray   archived:  local   probe: 10.5 mhz  image quality: good  narrative: posterior , axillary and anterior acoustic windows interrogated bilaterally. normal a lines throughout. no excess b-lines, air bronchograms or effusion. no consolidation  impression:normal lung us   "
"Normal"

Start report 
" point of care lung ultrasound  indication: rule out infiltrate/fluid  machine:  mindray   archived:  local   probe: 10.5 mhz  image quality: very good  narrative: posterior , axillar and anterior acoustic windows interrogated bilaterally. one area has borderline b-line. everything else is okay.  impression: normal.    t
"Very mild"


Start report 
"ultrasound  indication: rule out infiltrate/fluid  machine:  mindray   archived:  local   probe: 10.5 mhz  image quality: good  narrative: posterior , axillary and anterior acoustic windows interrogated bilaterally. normal a lines throughout. no excess b-lines, air bronchograms or effusion. no consolidation. no pneumonia.  impression:normal lung us       "
"Normal"


Start report 
"ultrasound  indication: rule out infiltrate/fluid  machine:  mindray   archived:  local   probe: 10.5 mhz  image quality: good  narrative: posterior , axillary and anterior acoustic windows interrogated bilaterally. normal a lines throughout. no excess b-lines, air bronchograms or effusion. no consolidation  impression:normal lung us          
"Normal"


Start report 
"ultrasound  indication: rule out infiltrate/fluid  machine:  mindray   archived:  local   probe: 10.5 mhz  image quality: good  narrative: posterior , axillary and anterior acoustic windows interrogated bilaterally. normal a lines throughout. no excess b-lines, air bronchograms or effusion. no consolidation  impression:normal lung us       abnormal lab results:   no abnormal labs in the last 3 months.      this document was transcribed by julianna c rojo, cms.    signature & attestation: all medical record entries made by the scribe were at my direction. i have reviewed the chart and agree that the record accurately reflects my person"
"Normal"


Start report 
"  point of care lung ultrasound  indication: rule out infiltrate/fluid  machine:  mindray   archived:  local   probe: 10.5 mhz  image quality:very good narrative: posterior , axillary and anterior acoustic windows interrogated bilaterally.   he has crowded air bronchograms and a small area of consolidation between 0.5 and 1 cm depth  in the right upper  acoustic windows posteriorly and in the upper most portion of the right axillary window.  does not have moth-eaten pleura or the coalescent b-lines typically seen with covid.  impression:  right upper lobe pneumonia     "
"Mild to Moderate"


Start report 
"  point of care limited  lung ultrasound  indication: rule out infiltrate/fluid  machine:  mindray   archived:  local   probe: 10.5 mhz  image quality: good   narrative: posterior , axillary and anterior acoustic windows interrogated bilaterally.  he has moth-eaten pleura and occasional excess b-lines bilaterally.  no effusion no consolidation.  impression:  very mild viral pneumonitis   "
"Very mild"

Start report 
"ultrasound  indication: rule out infiltrate/fluid  machine:  mindray   archived:  local   probe: 10.5 mhz  image quality: good  narrative: posterior , axillary and anterior acoustic windows interrogated bilaterally. normal a lines throughout. no excess b-lines, air bronchograms or effusion. no consolidation  impression:normal lung us    "
"Normal"


Start report 
"ultrasound unremarkable.     point of care lung ultrasound  indication: rule out infiltrate/fluid  machine:  mindray   archived:  local   probe: 10.5 mhz  image quality: good  narrative: posterior , axillary and anterior acoustic windows interrogated bilaterally. normal a lines throughout. no excess b-lines, air bronchograms or effusion. no consolidation  impression: normal lung us     "
"Normal"


Start report 
" point of care lung ultrasound  indication: rule out infiltrate/fluid  machine:  mindray   archived:  local   probe: 10.5 mhz  image quality: v good   narrative: posterior acoustic windows interrogated bilaterally. normal a lines, few linear airbronchgram lul only c/w atelectasis.,     impression likely viral infection  lul normal otherwise not covid     records reviewed:  nursing notes  relevant past records    "
"Very mild"


Start report 
"  point of care lung ultrasound  indication: rule out infiltrate/fluid  machine:  mindray   archived:  local   probe: 4.1mhz  image quality: ***  narrative: posterior , axillar and anterior acoustic windows interrogated bilaterally. increased breath sounds diffusely   no effusion. normal a lines. normal b lines  impression:***    *** "
"Normal"


Start report 
" point of care lung ultrasound  indication: rule out infiltrate/fluid  machine:  mindray   archived:  local   probe: 10.5 mhz  image quality: ***  narrative: posterior , axillar and anterior acoustic windows interrogated bilaterally. normal a lines. normal b lines. no profusion. no consolidation.  impression:normal us.  "
"Normal"


Start report 
"ultrasound  indication: rule out infiltrate/fluid  machine:  mindray   archived:  local   probe: 10.5 mhz  image quality:good  narrative: posterior , axillary and anterior acoustic windows interrogated bilaterally. normal a lines throughout. no excess b-lines, air bronchograms or effusion. no consolidation  impression:normal lung us  
"Normal"


Start report 
"  point of care lung ultrasound  indication: rule out infiltrate/fluid  machine:  mindray   archived:  local   probe: 10.5 mhz  image quality: good  narrative: posterior , axillar and anterior acoustic windows interrogated bilaterally. he does have borderline  increased b lines bilaterally in the axilla right > left   impression: possible mild pnemonitis       "
"Mild"


Start report 
"ultrasound  indication: rule out infiltrate/fluid  machine:  mindray   archived:  local   probe: 10.5 mhz  image quality: good  narrative: posterior , axillary and anterior acoustic windows interrogated bilaterally. normal a lines throughout. no excess b-lines, air bronchograms or effusion. no consolidation  impression:normal lung us  "
"Normal"


Start report 
"ultrasound  indication: rule out infiltrate/fluid  machine:  mindray   archived:  local   probe: 10.5 mhz  image quality: good  narrative: posterior , axillary and anterior acoustic windows interrogated bilaterally. normal a lines throughout. no excess b-lines, air bronchograms or effusion. no consolidation  impression:normal lung us   "
"Normal"


Start report 
"ultrasound.     limited bedside poc ultrasound radiology report  sutter medical center emergency department - sacramento    exam: poc lung ultrasound    indications: chest pain or shortness of breath    views: 2-3 views (as below)    probe: linear probe    image quality: good    procedure: using the linear array transducer i evaluated the bilateral anterolateral thorax in a systematic fashion revealing the presence of lung sliding and comet-tail artifact at the interrogated interspaces. m-mode shows normal anatomy and function.     impression: negative limited lung ultrasound with no pneumothorax identif"
"Normal"


Start report 
"  point of care limited  lung ultrasound  indication: rule out infiltrate/fluid  machine:  mindray   archived:  local   probe: 4.3 mhz  image quality: good   narrative: posterior , axillary and anterior acoustic windows interrogated bilaterally.  he he has patchy areas of at least moderate severity excess b-lines and moth-eaten pleura.  he also was fairly large areas that are not involved.  no effusion.  no consolidation.  bilateral changes.  worse on the left.       impression:  consistent with moderate severity bilateral covid pneumonitis    "
"Moderate"


Start report 
"point of care lung ultrasound  indication: rule out infiltrate/fluid  machine:  mindray   archived:  local   probe: 10.5 mhz  image quality: good  narrative: posterior , axillary and anterior acoustic windows interrogated bilaterally. ***  impression:***    mostly normal some areas of excess air bronchobrows on right.   occasion irreg plurea and b lines   mist blines of uper limit normal  both axxila upper llimt of b lines  impress  borderline us.     "
"Very mild"


Start report 
"ultrasound completed.     limited bedside poc ultrasound radiology report  sutter medical center emergency department - sacramento    exam: poc lung ultrasound    indications: bronchiolitis     views: 2-3 views (as below)    probe: linear probe    image quality: good    procedure: using the linear array transducer i evaluated the bilateral anterolateral thorax in a systematic fashion revealing the presence of lung sliding and comet-tail artifact at the interrogated interspaces. m-mode shows normal anatomy and function.     impression: negative limited lung ultrasound 
"Normal"


Start report 
"ultrasound  indication: rule out infiltrate/fluid  machine:  mindray   archived:  local   probe: 10.5 mhz  image quality: good   narrative: posterior , axillary and anterior acoustic windows interrogated bilaterally. excess short and long b-lines to left base in posterior window and left axillary window. increase air bronchogram*** to left base. right lung has excess short b-lines anteriorly with right lung otherwise normal.   impression:left pneumonitis with possible right anterior pneumonitis. no effusi"
"Moderate"


Start report 
"ultrasound  indication: rule out infiltrate/fluid  machine:  mindray   archived:  local   probe: 10.5 mhz  image quality: good   narrative: posterior , axillary and anterior acoustic windows interrogated bilaterally. excess short and long b-lines to left base in posterior window and left axillary window. increase air bronchograms in the  left base. right lung has excess short b-lines anteriorly with right lung otherwise normal.  no effusion. no consolidation   impression:left pneumonitis with possible right anterior pneumonitis.  "
"Moderate"


Start report 
"  point of care limited  lung ultrasound  indication: rule out infiltrate/fluid  machine:  mindray   archived:  local   probe: 10.5 mhz  image quality: good   narrative: posterior , axillary and anterior acoustic windows interrogated bilaterally. abnormal long b lines in left upper posterior and axillary window. no effusion or consolidation.  impression: abnormal consistent with focal pneumonitis   "
"Very mild"


Start report 
" bedside ultrasound of the posterior lungs showed no pleural irregularities, consolidations, or b lines consistent with covid findings   "           "
"Normal"


Start report 
"ultrasound  indication: rule out infiltrate/fluid  machine:  mindray   archived:  local   probe: 10.5 mhz  image quality: good    narrative: posterior , axillary and anterior acoustic windows interrogated bilaterally. normal a lines throughout. no excess b-lines, air bronchograms or effusion. no consolidation  impression:normal lung us "


Start report 
"ultrasound  indication: rule out infiltrate/fluid  machine:  mindray   archived:  local   probe: 10.5 mhz  image quality: good  narrative: posterior , axillary and anterior acoustic windows interrogated bilaterally. normal a lines throughout.  no consolidation or effusion.  he had 2 areas in the upper anterior feels with moth-eaten pleura and long coalescent b-lines.  impression:abormal lung us consistent with pleural scarring.  in the context of a positive covid test likely represents lung involvement of the covid.   "
"Very mild"


Start report 
"  point of care limited  lung ultrasound  indication: rule out infiltrate/fluid  machine:  mindray   archived:  local   probe: 10.5 mhz  image quality: good   narrative: posterior , axillary and anterior acoustic windows interrogated bilaterally.   impression: no evidence of pulmonary edema.    reviewed and electronically signed by paul walsh md.       medical decision making:   differential diagnosis includes [but is not limited to] viral uri, influenza, bronchiolitis, "
"Normal"


Start report 
"  point of care limited  lung ultrasound  indication: rule out infiltrate/fluid  machine:  mindray   archived:  local   probe: 10.5 mhz  image quality: good   narrative: posterior , axillary and anterior acoustic windows interrogated bilaterally. some air bronchograms on the right acoustic window, posterior. no effusion or consolidation. no b-lines.  impression: consistent with mild bronchitis or atelectasis.    "
"Mild"


Start report 
"  point of care limited  lung ultrasound  indication: rule out infiltrate/fluid  machine:  mindray   archived:  local   probe: 10.5 mhz  image quality: good   narrative: posterior , axillary and anterior acoustic windows interrogated bilaterally. some air bronchograms on the right acoustic window, posterior. no effusion or consolidation. no b-lines.  impression: consistent with mild bronchitis or atelectasis.  "
"Mild"


Start report 
"ultrasound  indication: rule out infiltrate/fluid  machine:  mindray   archived:  local   probe: 10.5 mhz  image quality: good  narrative: posterior , axillary and anterior acoustic windows interrogated bilaterally. normal a lines throughout. no excess b-lines, air bronchograms or effusion. no consolidation  impression:normal lung us   "
"Normal"


Start report 
"ultrasound  indication: rule out infiltrate/fluid  machine:  mindray   archived:  local   probe: 10.5 mhz  image quality: good  narrative: posterior , axillary and anterior acoustic windows interrogated bilaterally. normal a lines throughout. no excess b-lines, air bronchograms or effusion. no consolidation  impression:normal lung us   "
"Normal"


Start report 
" point of care lung ultrasound  indication: rule out infiltrate/fluid  machine:  mindray   archived:  local   probe: 10.5 mhz  image quality: good narrative: posterior , axillar and anterior acoustic windows interrogated bilaterally.  patchy areas of increased b lines. no effusion or consolidation  impression:virla pneumonitis      "
"Mild"

Start report 
"ultrasound  indication: rule out infiltrate/fluid  machine:  mindray   archived:  local   probe: 10.5 mhz  image quality: good  narrative: posterior , axillary and anterior acoustic windows interrogated bilaterally. normal a lines throughout. no excess b-lines, air bronchograms or effusion. no consolidation  impression:normal lung us   "
"Normal"


Start report 
"ultrasound  indication: rule out infiltrate/fluid  machine:  mindray   archived:  local   probe: 10.5 mhz  image quality:good   narrative: posterior , axillary and anterior acoustic windows interrogated bilaterally. normal a lines throughout. no excess b-lines, air bronchograms or effusion. no consolidation  impression:normal lung us   "
"Normal"


Start report 
"  point of care limited  lung ultrasound  indication: rule out infiltrate/fluid  machine:  mindray   archived:  local   probe: 10.5 mhz  image quality: good   narrative: posterior , axillary and anterior acoustic windows interrogated bilaterally. some excess b lines and air bronchograms bilaterally, worse on the right.   no effusion.     impression:consistent with bronchiolitis     "
"Mild"


Start report 
"ultrasound  indication: rule out infiltrate/fluid  machine:  mindray   archived:  local   probe: 10.5 mhz  image quality: good  narrative: posterior , axillary and anterior acoustic windows interrogated bilaterally. normal a lines throughout. no excess b-lines, air bronchograms or effusion. no consolidation  impression:normal lung us   "
"Normal"


Start report 
"point of care lung ultrasound  indication: rule out infiltrate/fluid  machine:  mindray   archived:  local   probe: 10.5 mhz  image quality: good  narrative: posterior , axillar and anterior acoustic windows interrogated bilaterally.   impression:normal    "
"Normal"


Start report 
"ultrasound  indication: rule out infiltrate/fluid  machine:  mindray   archived:  local   probe: 10.5 mhz  image quality: good  narrative: posterior , axillar and anterior acoustic windows interrogated bilaterally. no excess b lines, effusion or consolidaton  impression:normal   "
"Normal"


Start report 
"ultrasound  indication: rule out infiltrate/fluid  machine:  mindray   archived:  local   probe: 10.5 mhz  image quality: good  narrative: posterior , axillar and anterior acoustic windows interrogated bilaterally. no excess b lines, effusion or consolidaton  impression:normal  "
"Normal"


Start report 
"  point of care lung ultrasound  indication: rule out infiltrate/fluid  machine:  mindray   archived:  local   probe: 10.5 mhz  image quality: good  narrative: posterior , axillary and anterior acoustic windows interrogated bilaterally. normal a lines throughout. no excess b-lines, air bronchograms or effusion. no consolidation  impression:normal lung us         "
"Normal"


Start report 
"ultrasound  indication: rule out infiltrate/fluid  machine:  mindray   archived:  local   probe: 10.5 mhz  image quality: good  narrative: posterior and axillar acoustic windows interrogated bilaterally.   impression: diffuse a-lines, no evidence of b-lines, no effusion       "
"Normal"


Start report 
"ultrasound  indication: rule out infiltrate/fluid  machine:  mindray   archived:  local   probe: 10.5 mhz  image quality: good  narrative: posterior , axillary and anterior acoustic windows interrogated bilaterally. normal a lines throughout. no excess b-lines, air bronchograms or effusion. no consolidation  impression:normal lung us "   
"Normal"


Start report 
"ultrasound radiology report  sutter medical center emergency department - sacramento    exam: poc limited ultrasound of ***    indications: ***    views: 2-3 views (as below)    probe: linear    image quality: excellent        point of care limited lung ultrasound   indication: rule out infiltrate/fluid  machine:  mindray   archived:  local   probe: 9-3 mhz  image quality: good   narrative: posterior , axillary and anterior acoustic windows interrogated bilaterally. area of excess long b-lines in the right intrascapular window posteriorly. no effusion or consolidation. some moth eaten pleura bilaterally.     impression: abnormal lung us       reviewed and electronically signed b"
"Mild to Moderate"


Start report 
"point of care lung ultrasound  indication: rule out infiltrate/fluid  machine:  mindray   archived:  local   probe: 10.5 mhz  image quality: normal   narrative: posterior acoustic windows interrogated bilaterally.   impression: normal. normal a lines and b lines        "
"Normal"


Start report 
"  point of care limited  lung ultrasound  indication: rule out infiltrate/fluid  machine:  mindray   archived:  local   probe: 10.5 mhz  image quality: good   narrative: posterior , axillary and anterior acoustic windows interrogated bilaterally. b lines upper limit of normal. there are a couple of air bronchograms.   impression: us more c/w non-covid infection.          "
"Very mild"


Start report 
"  limited bedside poc ultrasound radiology report  sutter medical center emergency department - sacramento    exam: focus lung ultrasound, mindray     indications: cough     probe: 4.5mhz, 10.5mhz probe failed due to body habitus     image quality: poor     archives: local     procedure: using the probe i evaluated the bilateral anterolateral thorax in a systematic fashion revealing the presence of lung sliding and comet-tail artifact at the interrogated interspaces.      impression: borderline excess b line in right upper posterior acoustic window with thickening, subpleural less than 1cm. on the right there is an "
"Very mild"


Start report 
"ultrasound  indication: rule out infiltrate/fluid  machine:  mindray   archived:  local   probe: 10.5 mhz  image quality: good   narrative: posterior , axillary and anterior acoustic windows interrogated bilaterally.  no effusion. area of air bronchograms on the left upper lobe less than 1 cm, bilateral long b lines in the bases and anterior lung fields.  impression:  bilateral  long b lines in the bases and some airbronchogram? pneumonitis v bronchiolits v cld     "Moderate"


Start report 
"ultrasound radiology report  sutter medical center emergency department - sacramento    exam: focus lung ultrasound    indications: cough     probe: 10.5 mhz probe     image quality: good    procedure: ***     impression: upper limit of normal b lines on the right and left side    "      "
"Normal"


Start report 
"ultrasound  indication: rule out infiltrate/fluid  machine:  mindray   archived:  local   probe: 10.5 mhz  image quality: ***  narrative: posterior , axillary and anterior acoustic windows interrogated bilaterally. normal a lines throughout. no excess b-lines, air bronchograms or effusion. no consolidation  impression:normal lung us   
"Normal"


Start report 
"ultrasound  indication: rule out infiltrate/fluid  machine:  mindray   archived:  local   probe: 10.5 mhz  image quality: good  narrative: posterior , axillary and anterior acoustic windows interrogated bilaterally. normal a lines throughout. no excess b-lines, air bronchograms or effusion. no consolidation  impression: normal lung us        
"Normal"


Start report 
"ultrasound  indication: rule out infiltrate/fluid  machine:  mindray   archived:  local   probe: 10.5 mhz  image quality: adequate  narrative: posterior , axillary and anterior acoustic windows interrogated bilaterally.   impression:  lung exam without abnormality, no focal infiltrate identified.     medical decision making:   differential diagnosis includes [but is not limited to] viral uri, asthma/copd exacerbation, pneumonia, pulmonary embolism among others    in summary, this is a 5 month old female who presents with cough and rhinorrhea.  evaluation in the ed most pertinent for reassuring pe a"
"Normal"


Start report 
"ultrasound  indication: rule out infiltrate/fluid  machine:  mindray   archived:  local   probe: 10.5 mhz  image quality: adequate  narrative: posterior , axillary and anterior acoustic windows interrogated bilaterally.   impression:  lung exam without abnormality, no focal infiltrate identified.   
"Normal"


Start report 
"ultrasound   indication: rule out infiltrate/fluid  machine:  mindray   archived:  local   probe: 9-3 mhz  image quality: good   narrative: posterior , axillary and anterior acoustic windows interrogated bilaterally.  she has an area of mild the pleura with excess long coalescent b-lines on the right posterior intrascapular windows.  no effusion.  no consolidation.  impression: abnormal lung us focal unilateral viral pneumonitis   
"Mild"


Start report 
" point of care lung ultrasound  indication: rule out infiltrate/fluid  machine:  mindray   archived:  local   probe: 10.5 mhz  image quality:fair narrative: posterior , axillar and anterior acoustic windows interrogated bilaterally.   impression:excess b lines in all windows. based on bedside us, uncertain if it is viral pneumenitus or vs covid   "
"Moderate"


Start report 
"  point of care limited  lung ultrasound  indication: rule out infiltrate/fluid  machine:  mindray   archived:  local   probe: 10.5 mhz  image quality: good   narrative: posterior , axillary and anterior acoustic windows interrogated bilaterally. few b lines on the left base.  no effusion/consolidation   impression: mild focal pneumonitis left  "
"Mild"


Start report 
"  point of care lung ultrasound  indication: rule out infiltrate/fluid  machine:  mindray   archived:  local   probe: 10.5 mhz  image quality: good  narrative: posterior , axillary, and anterior acoustic windows interrogated bilaterally. pleural thickening with excess non coalescent and coalescent lines in right upper anterior acoustic window. minimillay increased coalescent  b lines posterior acoustic window.    impression resolving multifocal viral pneumonitis        
"Mild to Moderate"

Start report 
"ultrasound  indication: rule out infiltrate/fluid  machine:  mindray   archived:  local   probe: 10.5 mhz  image quality: ***  narrative: posterior , axillary and anterior acoustic windows interrogated bilaterally. normal a lines throughout. no excess b-lines, air bronchograms or effusion. no consolidation  impression:normal lung us     "
"Normal"


Start report 
" point of care lung ultrasound  indication: rule out infiltrate/fluid  machine:  mindray   archived:  local   probe: 10.5 mhz  image quality: good  narrative: posterior , axillar and anterior acoustic windows interrogated bilaterally. some increase b lines bilaterally, some exccess long b lines. mostly excess short b lines. no consolidation. no effusion.     "
"Very mild"


Start report 
"   point of care lung ultrasound  indication: rule out infiltrate/fluid  machine:  mindray   archived:  local   probe: 10.5 mhz  image quality: good  narrative: posterior , axillar and anterior acoustic windows interrogated bilaterally. some increase b lines bilaterally, some excess long b lines. mostly excess short b lines. no consolidation. no effusion.   impression:c/w covid or bronchiolitis    . "
"Very mild"


Start report 
"  point of care lung ultrasound  indication: rule out infiltrate/fluid  machine:  mindray   archived:  local   probe: 10.5 mhz  image quality: very good   narrative: posterior and axillary acoustic windows interrogated bilaterally. he has an area pleural thickening and irregularity placed are bronchograms consistent with subcentimeter consolidation on the right. he has diffusely increased excessive coalescent b-lines and associated mop eaten pleura bilaterally in the intrascapular areas and in the axillary bilaterally.  impression: small area of subcentimeter consolidation and associated bilateral diffuse pneumonitis worse on the right consistent with covid.  
"Moderate"


Start report 
" point of care lung ultrasound  indication: rule out infiltrate/fluid  machine:  mindray   archived:  local   probe: 10.5 mhz  image quality: very good  narrative: posterior examiner he and anterior acoustic windows interrogated bilaterally.  he has upper limit of normal to slightly abnormal b lines with minimal pleural thickening only in the right upper most aspect of the posterior acoustic window.  impression: fairly typical of what we would expect with rsv. does not appear to be i covid.          "
"Very mild"


Start report 
"  point of care limited  lung ultrasound  indication: rule out infiltrate/fluid  machine:  mindray   archived:  local   probe: 10.5 mhz  image quality: good   narrative: posterior , axillary and anterior acoustic windows interrogated bilaterally.minor excess short and some long b lines let base mostly no effusion no consolidation   impression:mild covid pneumonitis   "
"Mild"


Start report 
"indicated) point of care lung ultrasound  indication: rule out infiltrate/fluid  machine:  mindray   archived:  local   probe: 10.5 mhz   image quality: good   narrative: posterior  and axillary and anterior acoustic windows interrogated bilaterally.increased, mostly short b-lines left axillary  window.     impression: cw mild covid     medical decision ma"
"Mild"


Start report 
"ultrasound  indication: rule out infiltrate/fluid  machine:  mindray   archived:  local   probe: 10.5 mhz  image quality: ***  narrative: posterior , axillary and anterior acoustic windows interrogated bilaterally. normal a lines throughout. no excess b-lines, air bronchograms or effusion. no consolidation  impression:normal lung us   
"Normal"


Start report 
"ultrasound  indication: rule out infiltrate/fluid  machine:  mindray   archived:  local   probe: 10.5 mhz  image quality: good  narrative: posterior , axillary and anterior acoustic windows interrogated bilaterally. normal a lines throughout. no excess b-lines, air bronchograms or effusion. no consolidation  impression:normal lung us  " 
"Normal"


Start report  
"ultrasound   indication: rule out infiltrate/fluid  machine:  mindray   archived:  local   probe: 9-3 mhz  image quality: good   narrative: posterior , axillary and anterior acoustic windows interrogated bilaterally.  the left posterior acoustic windows show increased air bronchograms diffusely.  no excess b-lines.  left axilla is normal.  the right posterior acoustic window shows areas of excess long b-lines obliterating the a lines.  in addition to this there was some hepatization and irregular air bronchograms.  the consolidation is greater than 1 centimetre in depth.  there is no effusion.  impression: abnormal lung us consistent with right lower lobe pneumonia    
"Mild to Moderate"


Start report 
"point of care lung ultrasound  indication: rule out infiltrate/fluid  machine:  mindray   archived:  local   probe: 10.5 mhz  image quality: excellent  narrative: posterior , axillary and anterior acoustic windows interrogated bilaterally.  excess b  lines diffusely in both right and left intrascapular regions.   impression: moderate pneumonitis consistent with covid              "
"Moderate"


Start report 
" limited bedside poc ultrasound radiology report  sutter medical center emergency department - sacramento    exam: poc lung ultrasound    indications: irritability    views: 2-3 views (as below)    probe: linear probe    image quality: good    procedure: using the linear array transducer i evaluated the bilateral posterolateral thorax in a systematic fashion revealing the presence of lung sliding with pleural thickening and b-lines in multiple lung fields.      impression: positive findings for covid-19.   these images were archived and i independently interpreted the images at the bedside.  
"Moderate"


Start report 
"ultrasound radiology report  sutter medical center emergency department - sacramento    exam: poc lung ultrasound    indications: cough    views: 2-3 views (as below)    probe: linear probe    image quality: good    procedure: using the linear array transducer i evaluated the bilateral anterolateral thorax in a systematic fashion revealing the presence of lung sliding and at the interrogated interspaces. m-mode shows normal anatomy and function. there are multiple mild b-lines in various lung fields bilaterally consistent with mild coronavirus pneumonitis     impression: positive limited lung ultrasound with no pneumothorax identified. exam consistent with mild covid pneumonitis. 
"Mild"


Start report 
" point of care lung ultrasound  indication: rule out infiltrate/fluid  machine:  mindray   archived:  local   probe: 10.5 mhz  image quality: good   narrative: posterior , axillar and anterior acoustic windows interrogated bilaterally. b lines on right axillar   impression:concernign for pulmonary edema vs pneumonia,  "
"Very mild"

Start report 
"ed procedures:   point of care lung ultrasound  indication: rule out infiltrate/fluid  machine:  mindray   archived:  local   probe: 10.5 mhz  image quality: good   narrative: posterior , axillar and anterior acoustic windows interrogated bilaterally.  exces b lines on right axillary window   impression:concernign for pulmonary edema vs pneumonia,
"Mild to Moderate"


Start report 
"   point of care lung ultrasound  indication: rule out infiltrate/fluid  machine:  mindray   archived:  local   probe: 10.5 mhz  image quality: very good  narrative: posterior , axillar and anterior acoustic windows interrogated bilaterally. diffuse excess b-line and pleural thickening bilaterally in all lung windows.  impression:consistent with covid.          "
"Moderate"


Start report 
"  point of care lung ultrasound  indication: rule out infiltrate/fluid  machine:  mindray   archived:  local   probe: 4.1mhz  image quality:   narrative: posterior , axillar and anterior acoustic windows interrogated bilaterally. she has excess long and short b lines and moth eaten pleura bilaterally   impression: viral pneumonitis c/w covid          "
"Mild to Moderate"


Start report 
"  point of care lung ultrasound  indication: rule out infiltrate/fluid  machine:  mindray   archived:  local   probe: 10.5 mhz  image quality: good  narrative: posterior , axillar and anterior acoustic windows interrogated bilaterally. normal a lines, no excess b lines, no effusion or consolidation   impression:normal  "
"Normal"

Start report 
" point of care lung ultrasound  indication: rule out infiltrate/fluid  machine:  mindray   archived:  local   probe: 10.5 mhz  image quality: ***  narrative: posterior , axillar and anterior acoustic windows interrogated bilaterally. ***  impression: lung upper limit has short b - lines in the left axilla. no effusion. no consolidation.      "
"Normal"


Start report 
"ultrasound  indication: rule out infiltrate/fluid  machine:  mindray   archived:  local   probe: 10.5 mhz  image quality: very good  narrative: posterior , axillar and anterior acoustic windows interrogated bilaterally. lung upper limit has short b - lines in the left axilla. no effusion. no consolidation.   impression: normal   "
"Normal"


Start report 
"    point of care lung ultrasound  indication: rule out infiltrate/fluid  machine:  mindray   archived:  local   probe: 10.5 mhz  image quality: fair  narrative: posterior , axillar and anterior acoustic windows interrogated bilaterally.   impression: normal   "
"Normal"


Start report 
"  point of care lung ultrasound  indication: rule out infiltrate/fluid  machine:  mindray   archived:  local   probe: 10.5 mhz  image quality: ***  narrative: posterior, axillary and anterior acoustic windows interrogated bilaterally.   impression:excess b-lines in two intercostal spaces, mild bronchiolitis or pneumonitis. no consolidation, no effusion.      "
"Mild"


Start report 
"point of care lung ultrasound  indication: rule out infiltrate/fluid  machine:  mindray   archived:  local   probe: 10.5 mhz  image quality: excellent  narrative: posterior , axillary and anterior acoustic windows interrogated bilaterally.   impression:excess b lines mostly in the left. b lines slightly to the right. no effusion. no consolidation.                 "
"Mild to Moderate"


Start report 
"point of care lung ultrasound  indication: rule out infiltrate/fluid  machine:  mindray   archived:  local   probe: 10.5 mhz  image quality: good  narrative: posterior , axillar and anterior acoustic windows interrogated bilaterally.  impression: normal us "      "
"Normal"


Start report 
" point of care lung ultrasound  indication: rule out infiltrate/fluid  machine:  mindray   archived:  local   probe: 10.5 mhz  image quality: good  narrative: posterior , axillar and anterior acoustic windows interrogated bilaterally.   impression:no b-lines to suggest pulmonary edema or covid-19 pna.      "
"Normal"

Start report 
"ultrasound   indication: rule out infiltrate/fluid  machine:  mindray   archived:  local   probe: 10-3mhz  image quality: good   narrative: posterior , axillary and anterior acoustic windows interrogated bilaterally. normal a lines throughout. no excess b-lines, or effusion. no consolidation.  she does have some linear air bronchograms bilaterally consistent with atelectasis or early bronchiolitis.    impression:abnormal lung us however there are insufficient other findings to label bronchiolitis at this point and for now we will take a watchful waiting approach. "
"Very mild"


Start report 
"ultrasound    indication: rule out infiltrate/fluid  machine:  mindray   archived:  local   probe: 9-3 mhz  image quality: good  narrative: posterior , axillary and anterior acoustic windows interrogated bilaterally. excess b lines in right axilla. moth-eaten pleura bilaterally in the intrascapular windows.   impression: abnormal lung us consistent with mild covid pneumonitis  "
"Mild"


Normal;heading 1;heading 2;heading 3;heading 4;heading 5;heading 6;heading 7;heading 8;heading 9;caption;Title;Subtitle;Strong;Emphasis;Placeholder Text;No Spacing;Light Shading;Light List;Light Grid;Medium Shading 1;Medium Shading 2;Medium List 1;Medium List 2;Medium Grid 1;Medium Grid 2;Medium Grid 3;Dark List;Colorful Shading;Colorful List;Colorful Grid;Light Shading Accent 1;Light List Accent 1;Light Grid Accent 1;Medium Shading 1 Accent 1;Medium Shading 2 Accent 1;Medium List 1 Accent 1;Revision;List Paragraph;Quote;Intense Quote;Medium List 2 Accent 1;Medium Grid 1 Accent 1;Medium Grid 2 Accent 1;Medium Grid 3 Accent 1;Dark List Accent 1;Colorful Shading Accent 1;Colorful List Accent 1;Colorful Grid Accent 1;Light Shading Accent 2;Light List Accent 2;Light Grid Accent 2;Medium Shading 1 Accent 2;Medium Shading 2 Accent 2;Medium List 1 Accent 2;Medium List 2 Accent 2;Medium Grid 1 Accent 2;Medium Grid 2 Accent 2;Medium Grid 3 Accent 2;Dark List Accent 2;Colorful Shading Accent 2;Colorful List Accent 2;Colorful Grid Accent 2;Light Shading Accent 3;Light List Accent 3;Light Grid Accent 3;Medium Shading 1 Accent 3;Medium Shading 2 Accent 3;Medium List 1 Accent 3;Medium List 2 Accent 3;Medium Grid 1 Accent 3;Medium Grid 2 Accent 3;Medium Grid 3 Accent 3;Dark List Accent 3;Colorful Shading Accent 3;Colorful List Accent 3;Colorful Grid Accent 3;Light Shading Accent 4;Light List Accent 4;Light Grid Accent 4;Medium Shading 1 Accent 4;Medium Shading 2 Accent 4;Medium List 1 Accent 4;Medium List 2 Accent 4;Medium Grid 1 Accent 4;Medium Grid 2 Accent 4;Medium Grid 3 Accent 4;Dark List Accent 4;Colorful Shading Accent 4;Colorful List Accent 4;Colorful Grid Accent 4;Light Shading Accent 5;Light List Accent 5;Light Grid Accent 5;Medium Shading 1 Accent 5;Medium Shading 2 Accent 5;Medium List 1 Accent 5;Medium List 2 Accent 5;Medium Grid 1 Accent 5;Medium Grid 2 Accent 5;Medium Grid 3 Accent 5;Dark List Accent 5;Colorful Shading Accent 5;Colorful List Accent 5;Colorful Grid Accent 5;Light Shading Accent 6;Light List Accent 6;Light Grid Accent 6;Medium Shading 1 Accent 6;Medium Shading 2 Accent 6;Medium List 1 Accent 6;Medium List 2 Accent 6;Medium Grid 1 Accent 6;Medium Grid 2 Accent 6;Medium Grid 3 Accent 6;Dark List Accent 6;Colorful Shading Accent 6;Colorful List Accent 6;Colorful Grid Accent 6;Subtle Emphasis;Intense Emphasis;Subtle Reference;Intense Reference;Book Title;Bibliography;TOC Heading;
