# Supplementary material for: Point-of-care lung ultrasound predicts hyperferritinemia and hospitalization, but not elevated troponin in SARS-CoV-2 viral pneumonitis in children
Source: Sci Rep. 2024 Mar 11;14:5899. doi: 10.1038/s41598-024-55590-9 (PMC10928070; doi:10.1038/s41598-024-55590-9)
Supplement: Supplementary file 10 — Supplementary Information 10. [file 41598_2024_55590_MOESM10_ESM.pdf]

```
//
// ologit the effect of blood tests on outcomes
//
/*
**# ologit discharge/ward/picu  versus variable
foreach var in l_abs_lymphocyte l_abs_neutrophil l_alb  l_bnp l_bun l_cbc_wbc l_creatinine l_crp elev_dimer elev_troponin l_esr l_ferritin l_hematocrit l_ldh l_lft_alt l_lft_ast l_platelet l_procalcitonin l_sodium l_troponin   l_ua_rbc l_ua_sg l_ua_wbc {

 ologit ord_admit `var' triage_imp    //bicarb dropped for non-convergence

}
*/


// ologit discharge/ward/picu   substantially ourtperfomred logit v admission/discharge


**# Significant variables form above carried forward (manual) after finding this model in general led to the best fit

foreach var in   l_alb l_bun l_cbc_wbc l_hematocrit l_crp elev_dimer elev_troponin  l_ferritin l_lft_alt l_lft_ast   {

 ologit ord_admit `var'  triage_imp

  linktest

}
//Next set of diagnostics  collinearity as measured by variance inflation factor
  collin l_alb triage_imp
  collin l_bun triage_imp
  collin l_cbc_wbc triage_imp
  collin l_hematocrit triage_imp
  collin l_crp triage_imp
  collin elev_dimer  triage_imp
  collin elev_troponin  triage_imp
  collin l_ferritin triage_imp
  collin l_lft_alt triage_imp
  collin l_lft_ast  triage_imp

 //
  // Spc/fit tests work  despite ologit actually being multi-eqtn




 foreach var in   l_alb l_bun l_cbc_wbc l_hematocrit l_crp elev_dimer elev_troponin  l_ferritin l_lft_alt l_lft_ast   {

 omodel logit ord_admit `var'  triage_imp

 // manually checked and ok

}

 //The following blood tests failed the screening process to be included in this part of the analysis: ESR, CRP, Procalcitonin, Albumin, Hematocrit, WBC, Pro-BNP, and Troponin-


 lab def  outcome 0 "Discharged"   1 "Admit to Ward" 2 "Admit to PICU"
 lab val ord_admit outcome

```

```
 //  Graph individually   l_alb l_bun l_cbc_wbc l_hematocrit l_crp elev_dimer elev_troponin l_ferritin    l_lft_alt l_lft_ast   {


    ologit ord_admit l_alb  triage_imp

    margins ,at( l_alb = (0(0.5)5)  triage_imp=(3))
    marginsplot ,noci
     graph save "Graph" "F:\2023_11_November\Graph_l_alb.gph" ,replace


//

     ologit ord_admit l_bun  triage_imp

    margins ,at(l_bun=(0(5)40)  triage_imp=(3))
    marginsplot ,noci
     graph save "Graph" "F:\2023_11_November\Graph_l_bun.gph" ,replace

//
 ologit ord_admit l_cbc_wbc  triage_imp

    margins ,at(l_cbc_wbc=(3.5(5)38) triage_imp=(3))
    marginsplot ,noci
     graph save "Graph" "F:\2023_11_November\Graph_l_cbc_wbc.gph", replace

//

ologit ord_admit l_hematocrit triage_imp

    margins ,at(l_hematocrit=(27(3)51) triage_imp=(3))
    marginsplot ,noci
graph save "Graph" "F:\2023_11_November\Graph_l_hematocrit.gph",replace

//

ologit ord_admit l_crp triage_imp

    margins ,at(l_crp=(0(10)350) triage_imp=(3))
    marginsplot ,noci
graph save "Graph" "F:\2023_11_November\Graph_l_crp.gph",replace

//



ologit ord_admit l_ferritin triage_imp

    margins ,at(l_ferritin=(0(100)1500) triage_imp=(3))
    marginsplot ,noci
graph save "Graph" "F:\2023_11_November\Graph_l_ferritin.gph",replace

//

ologit ord_admit l_lft_alt triage_imp

    margins ,at(l_lft_alt=(5(10)200) triage_imp=(3))
    marginsplot ,noci
graph save "Graph" "F:\2023_11_November\Graph_l_lft_alt.gph",replace

//

ologit ord_admit l_lft_ast triage_imp
```

```
120
121         margins ,at(l_lft_ast=(5(10)200) triage_imp=(3))
122         marginsplot ,noci
123     graph save "Graph" "F:\2023_11_November\Graph_l_lft_ast.gph",replace
124
125     //
126
127
128
129     ologit ord_admit elev_dimer triage_imp
130
131         margins ,at(elev_dimer=(0/1) triage_imp=(3))
132         marginsplot ,noci
133     graph save "Graph" "F:\2023_11_November\Graph_elev_dimer.gph",replace
134
135     //
136      ologit ord_admit elev_troponin triage_imp
137
138         margins ,at(elev_troponin=(0/1) triage_imp=(3))
139         marginsplot ,noci
140     graph save "Graph" "F:\2023_11_November\Graph_elev_troponin.gph",replace
141
142
143
144     //
145     ologit ord_admit l_bnp triage_imp
146
147         margins ,at(l_ldh=(0(20)500) triage_imp=(3))
148         marginsplot ,noci
149     graph save "Graph" "F:\2023_11_November\Graph_l_dh.gph",replace
150
151     //
152
153     grc1leg  "F:\2023_11_November\Graph_elev_troponin.gph" "F:\2023_11_November\Graph_elev_dimer.gph"
        "F:\2023_11_November\Graph_l_cbc_wbc.gph"  "F:\2023_11_November\Graph_l_lft_ast.gph"
        "F:\2023_11_November\Graph_l_ferritin.gph" "F:\2023_11_November\Graph_l_crp.gph"
        "F:\2023_11_November\Graph_l_hematocrit.gph"  "F:\2023_11_November\Graph_l_lft_alt.gph"
        "F:\2023_11_November\Graph_l_bun.gph" "F:\2023_11_November\Graph_l_alb.gph"
154
```
