# Supplementary material for: Point-of-care lung ultrasound predicts hyperferritinemia and hospitalization, but not elevated troponin in SARS-CoV-2 viral pneumonitis in children
Source: Sci Rep. 2024 Mar 11;14:5899. doi: 10.1038/s41598-024-55590-9 (PMC10928070; doi:10.1038/s41598-024-55590-9)

S10 Comparative reading performance by different ultrasonographers

raw Stata output follows

Kruskal–Wallis equality-of-populations rank test

us_tech Obs Rank sum

-

PEM-1 277 58912.50

PEM-2 8 1763.00

Resident 112 23209.50

Gen EM 30 7493.00

chi2(3) = 2.910

Prob = 0.4057

chi2(3) with ties = 3.278

Prob = 0.3507

We also performed ordinal logistic regression with ultrasound classification as the dependent and the ultrasonographer and triage category as the independent variables. This addresses the concern that some physicians (particularly the general emergency physicians) would reserve lung ultrasound for sicker appearing patients.

[Annotated raw Stata output follows]

ologit f_score i.us_tech triage_imp

Iteration 0: Log likelihood = -637.76787

Iteration 1: Log likelihood = -620.29653

Iteration 2: Log likelihood = -620.15952

Iteration 3: Log likelihood = -620.15947

Ordered logistic regression Number of obs = 427

LR chi2(4) = 35.22

Prob > chi2 = 0.0000 [Overall model is significant versus an empty model]

Log likelihood = -620.15947 Pseudo R2 = 0.0276

f_score Coefficient Std. err. z P>z [95% conf. interval]

[Ultrasound categorization]

us_tech [Who did the ultrasound]

PEM-2 -.5575707 .7146467 -0.78 0.435 -1.958252 .8431112 [PEM-1 is referent (=1) the others are compared to him]

Resident -.1410172 .2094994 -0.67 0.501 -.5516286 .2695941

Gen EM .4657877 .377945 1.23 0.218 -.2749709 1.206546

triage_imp -.7354571 .1320448 -5.57 0.000 -.9942603 -.476654 [triage category as a numeric variable, 1 most severe, 5 least severe]

This shows the severity of illness, not who did the scan, to significantly predict the ultrasound classification. An ordinal model without triage category showed similar results.


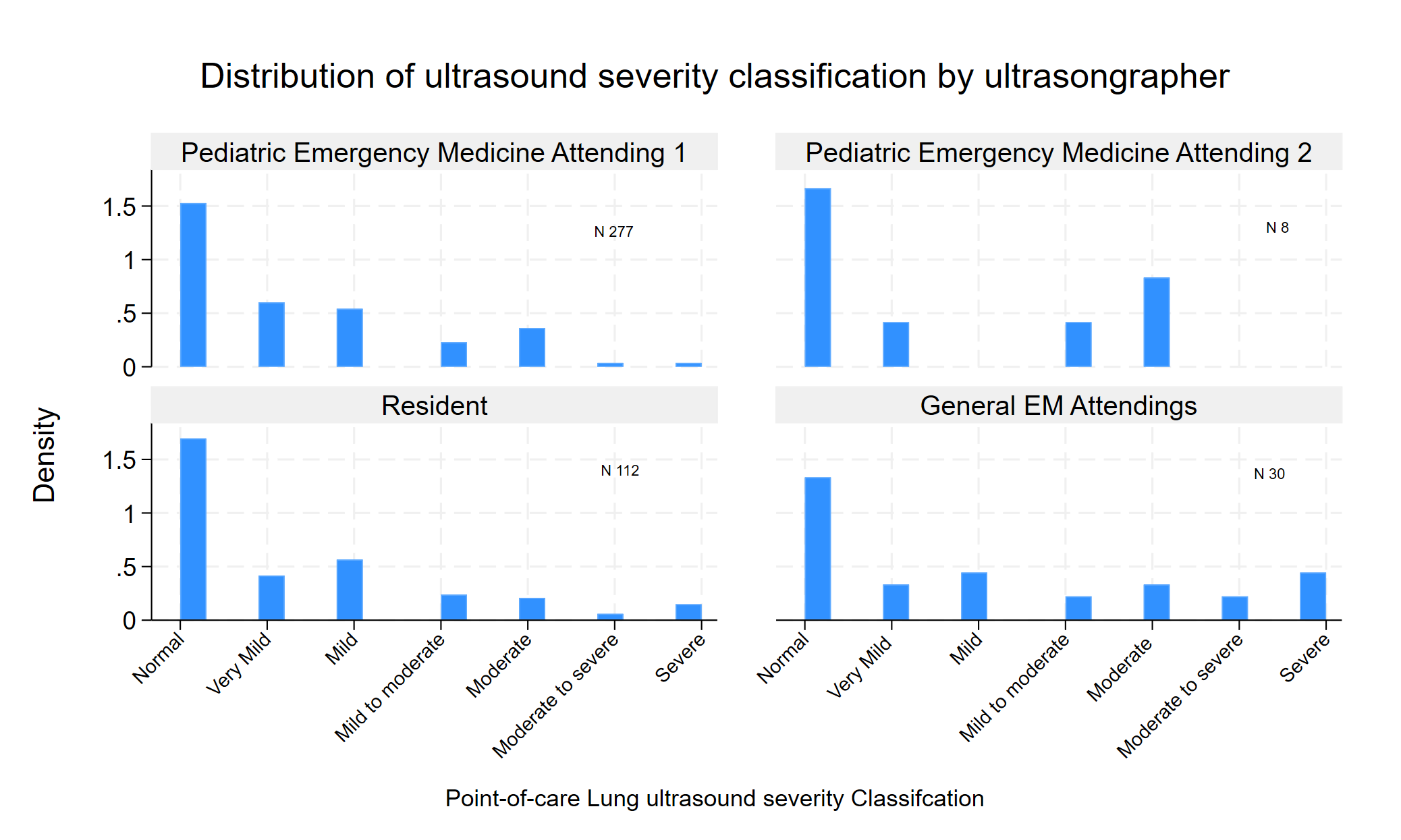

Supplement: Supplementary file 11 — Supplementary Information 11. [file 41598_2024_55590_MOESM11_ESM.docx]
