# Supplementary material for: Point-of-care lung ultrasound predicts hyperferritinemia and hospitalization, but not elevated troponin in SARS-CoV-2 viral pneumonitis in children
Source: Sci Rep. 2024 Mar 11;14:5899. doi: 10.1038/s41598-024-55590-9 (PMC10928070; doi:10.1038/s41598-024-55590-9)
Supplement: Supplementary file 10 [file 41598_2024_55590_MOESM4_ESM.odt]

Extended Table 3 

| Part A SARS-CoV-2 positive | Point of care lung ultrasound severity | Trend test p-value |
| --- | --- | --- |
| Blood test | Normal | Very  Mild | Mild | Mild to moderate | Moderate | Moderate to severe & severe |
| N | 51 | 19 | 24 | 3 | 14 | 7 |  |
| CRP μg/mL | 43.2 (23, 63.3) | 11.3 (4.01, 28.7) | 6.1 (6.1, 6.1) | 16.0 (15.1,16.8) | 3.2 (1.6, 7.7) | 25.5 (19.2, 51.3) | 0.74 |
| ESR mm/hour | 93 (93, 93) | 12  (8, 15) | 24 (14, 33) | 16  (16, 16) | 17 (3, 30) | 52 (42, 62) | 0.52 |
| Procalcitonin ng/mL | 0.14 (0.14, 0.14) | 0.09 (0.08, 0.09) | 0.02  (0.02, 0.02) | 0.09 (0.09,0.0) | 0.14 (0.04, 0.16) | 0.18  (0.10, 0.27) | 0.17 |
| Ferritin  ng/mL | 214  (214, 214) | 86 (77, 169) | 124  (83, 165) | 121  (116,125) | 336  (40, 881) | 585 (558, 675) | 0.01 |
| LDH iu/L | 298 (298, 298) | 238  (202, 273) | 314 (203, 424) | . (., .) | 388 (357, 508) | 500  (382, 549) | 0.03 |
| ALT iu/L | 31 (31, 31) | 29 (12, 46) | 19  (16, 29) | 50  (30, 70) | 41  (37, 56) | 44 (42, 84) | 0.02 |
| AST iu/L | 31  (31, 31) | 37 (11, 63) | 18 (12, 37) | 45 (33, 57) | 45  (17, 94) | 67 (63, 71) | 0.03 |
| Albumin  mg/ml | 3.9 (3.9, 3.9) | 4.05 (4, 4.1) | 3.95 (3.4, 4.15) | 4  (3.9, 4.1) | 3.65 (3.5, 3.7) | 3.2 (3, 3.5) | 0.01 |
| Hematocrit % | 37  (34.8, 39) | 40  (36, 44) | 38 (35, 43) | 41  (40, 42) | 40 (36, 45) | 46  (41, 46) | 0.10 |
| WBC K/uL | 8.9 (3.6, 14.1) | 9.8 (8.6, 10.6) | 5.6  (5.1, 6.6) | 7.1  (3.8,10.4) | 8.6 (4.4, 11.2) | 8.9 (5.1, 11.4) | 0.96 |
| Pro-BNP   pg/ml | .  (., .) | . (., .) | .  (., .) | .  (., .) | 771  (232, 1,276) | 2,394 (2,394, 2,394) | 0.18 |
| Creatinine mg/dL | 0.33  (0.33, 0.33) | 0.62 (0.37, 0.86) | 0.72 (0.37, 0.86) | 0.79 (0.75,0.83) | 0.26 (0.19, 0.94) | 0.96  (0.58, 1.16) | 0.27 |
| Troponin I (elevated) | 0  (0%) | 0 (0%) | 0 (0%) | 0 (0%) | 4 (50%) | 0 (0%) | 0.35 |
| D-dimer (elevated) | 1 (100%) | 2 (100%) | 1 (50%) | 1 (50%) | 4 (67%) | 5  (100%) | 0.83 |
|  |  |  |  |  |  |  |  |
| Part  B SARS-CoV-2 Negative | Point of care lung ultrasound severity |
| Blood test | Normal | Very  Mild | Mild | Mild to moderate | Moderate | Moderate-severe & Severe | Trend test p-value |
| N | 15 | 11 | 11 | 39 | 19 | 3 |  |
| LCRP μg/mL | 21  (4.8, 76.2) | 12 (0.3, 56.6) | 12.2 (7.4, 36.7) | 19.7 (3.5, 36.5) | 5.75  (4.0, 7.5) | .  (., .) | 0.75 |
| ESR mm/hour | 29  (11, 46) | .  (., .) | 15  (9, 21) | . (., .) | .  (., .) | .  (., .) | 0.43 |
| Procalcitonin  ng/mL | 0.98  (.08, 1.87) | 0.06 (0.04, 3.6) | 0.22  (0.04, 0.41) | 0.07 (0.07, .47) | 0.07 (0.07, 0.07) | . (., .) | 0.72 |
| Ferritin  ng/mL | 106 (106, 106) | 164  (77, 174) | 38 (28, 48) | 40  (40, 40) | . (., .) | .  (., .) | 0.94 |
| LDH  iu/L | 306 (306, 306) | . (., .) | 221 (174, 268) | . (., .) | . (., .) | .  (., .) | 0.31 |
| ALT  iu/L | 31 (31, 31) | .  (., .) | 17 (17, 17) | 26  (20.5, 29.5) | . (., .) | .  (., .) | 0.27 |
| AST    iu/L | 50  (50, 50) | . (., .) | 14  (14, 14) | 23  (16, 30) | .  (., .) | .  (., .) | 0.63 |
| Albumin  mg/ml | 3.9 (3.9, 3.9) | 3.1  (3.1, 3.1) | 3.4  (3.4, 3.4) | 4.1  (3.9, 4.3) | . (., .) | .  (., .) | 0.06 |
| Hematocrit  % | 44  (38, 49) | 40 (37, 46) | 36 (35, 38) | 39  (37, 40) | 36  (34, 39) | . (., .) | 0.55 |
| WBC  K/uL | 9.9 (8.5, 11.3) | 13.5 (9.4, 17.1) | 12.9  (6.6, 17.6) | 13.8  (8.9, 14.9) | 15.7  (10.1, 16.8) | . (., .) | 0.70 |
| Pro-BNP   pg/ml | 551 (551, 551) | 354 (58, 1515) | 36 (36, 36) | . (., .) | .  (., .) | .  (., .) | 0.67 |
| Creatinine mg/dL | 0.48  (0.48, 0.48) | . (., .) | 0.3 (0.3, 0.3) | 0.71 (0.5, 0.72) | .  (., .) | .  (., .) | 0.18 |
| Troponin-I (elevated) | 0  (0%) | 1  (33%) | 0  (0%) | 0 (0%) | .  (., .) | .  (., .) | 1.00 |
| D-dimer (elevated) | 1 (100%) | 0  (0%) | 1 (33%) | .  (., .) | .  (., .) | .  (., .) | 0.52 |
|  |
| Part  C All comers | Point of care lung ultrasound severity |
| Blood test | Normal | Very  Mild | Mild | Mild to moderate | Moderate | Moderate-severe & Severe | Trend test p-value |
| N | 200 | 68 | 68 | 30 | 42 | 19 |
| CRP  μg/mL | 13.6 (5.2, 29.8) | 19.65 (4.5, 51.65) | 7.5 (6.1, 7.8) | 13.6 (6.4, 36.7) | 6.8  (3, 12.2) | 22.1 (7.7, 28.9) | 0.68 |
| ESR  mm/hour | 49 (42, 72.5) | 14  (11, 19) | 33 (14, 62) | 24 (11, 51.5) | 9 (4, 30) | 52 (42, 62) | 0.31 |
| Procalcitonin  ng/mL | 0.06 (0.06, 0.07) | 0.08 (0.05, 0.19) | 0.06 (0.02, 0.11) | 0.08  (0.04, 2.06) | 0.14  (0.04, 0.27) | 0.14  (0.11, 0.23) | 0.12 |
| Ferritin  ng/mL | 141  (40, 214) | 91  (73, 126) | 165  (83, 192) | 125  (77, 164) | 67  (28, 545) | 572 (123,,050) | 0.12 |
| LDH  iu/L | 306 (253 ,315) | 239  (203, 289) | 228  (203, 306) | 247  (209, 269) | 306  (245, 357) | 441  (311, 549) | 0.05 |
| AST   iu/L | 26  (17, 33) | 25  (16, 46) | 18  (16, 35) | 23  (16, 30) | 21  (19, 56) | 56  (42, 65) | 0.01 |
| ALT  iu/L | 26  (17, 31) | 43  (18, 55) | 17  (8, 25) | 21  (15, 57) | 32 (16, 54) | 64 (41, 71) | 0.01 |
| Albumin  mg/ml | 3.8  (3.6, 4.1) | 4.0  (3.6, 4.0) | 3.8  (3, 4.1) | 4.0  (31, 4.5) | 3.7  (3.4, 3.9) | 3.6  (3.2, 3.8) | 0.14 |
| Hematocrit  % | 38  (37,  40) | 38  (37, 45) | 38  (34, 40) | 40  (38, 44) | 37  (35 ,40 ) | 41  (37, 46) | 0.79 |
| WBC  K/uL | 9.7  (6.9, 13.8) | 8.5 (7.4 ,11.2) | 9.05 (5.6, 14.1) | 10.4  (6.6, 15.6) | 9.15  (45.0 ,11.6) | 11.1  (6.3, 11.4) | 0.89 |
| Lymphocyte  K/uL | 2.6 (2.6, 2.6) | 1.4 (1.3, 1.5) | 0.9 (0.9,0 .9) | 4.7 (2.4, 6.9) | 1.9 (1.5, 2.6) | 1.6  (1.3, 3.8) | 0.95 |
| Neutrophil  K/uL | 4.4  (4.4, 4.4) | 3.0  (2.2, 3.7) | 10.8 (10.8, 10.8) | 3.0 (2.5, 3.5) | 4.4  (2.7, 21.6) | 6.2  (4.35, 7.2) | 0.32 |
| Pro-BNP  pg/ml | 170 (40, 2918) | 346.5 (142, 551) | 1,400  (1400, 1400) | 78 (58, 354) | 232  (64, 1276) | 2,394 (200, 9,319) | 0.32 |
| Creatinine mg/dL | 0.71  (0.38, 0.88) | 0.52 (0.48, 0.58) | 0.77 (0.62, 0.86) | 0.68 (0.45, 0.83) | 0.34 (0.26, 0.81) | 0.62 (0.39, 0.96) | 0.54 |
| Troponin-I (elevated) | 3 (38%) | 1 (17%) | 0 (0%) | 1 (13%) | 4 (29%) | 0 (0%) | 0.29 |
| D-dimer (elevated) | 3 (43%) | 4 (80%) | 2 (40%) | 2 (29%) | 8 (62%) | 8 (100%) | 0.05 |

Table 3. Laboratory values observed by the severity of lung ultrasound. Median (Interquartile range)  Part A –SARS-CoV-2 positive only, Part B –Covid Negative, Part C ­-All-comers. Covid  unknown data were all non-significant (data not shown) . LDH lactate dehydrogenase, AST aspartate aminotransferase, ALT alanine transaminase, ESR erythrocyte sedimentation rate, CRP c-reactive protein, WBC white blood cell count, ProBNP pro-b-natriuretic peptide, OR odds ratio, NC not calculable, LUS lung ultrasound, iu/l international units per liter, K/uL thousands per cubic millimeter, NAAT Nucleic acid amplification test. Rows removed for all missing data. No data is represented by ‘.(.,.)’.
